# Extracellular Microenvironment Alterations in Ductal Carcinoma In Situ and Invasive Breast Cancer Pathologies by Multiplexed Spatial Proteomics

**DOI:** 10.3390/ijms25126748

**Published:** 2024-06-19

**Authors:** Taylor S. Hulahan, Laura Spruill, Elizabeth N. Wallace, Yeonhee Park, Robert B. West, Jeffrey R. Marks, E. Shelley Hwang, Richard R. Drake, Peggi M. Angel

**Affiliations:** 1 Department of Cell and Molecular Pharmacology and Experimental Therapeutics, Medical University of South Carolina, Charleston, SC 29425, USA; hulahan@musc.edu (T.S.H.); wallaeli@musc.edu (E.N.W.); draker@musc.edu (R.R.D.); 2 Department of Pathology and Laboratory Medicine, Medical University of South Carolina, Charleston, SC 29425, USA; spruill@musc.edu; 3 Department of Biostatistics and Medical Informatics, University of Wisconsin-Madison, Madison, WI 53726, USA; ypark56@wisc.edu; 4 Department of Pathology Clinical, Stanford University, Stanford, CA 94305, USA; rbwest@stanford.edu; 5 Department of Surgery, Duke University, Durham, NC 27710, USA; jeffrey.marks@duke.edu (J.R.M.); shelley.hwang@duke.edu (E.S.H.)

**Keywords:** extracellular matrix (ECM), tumor microenvironment, ductal carcinoma in situ (DCIS), invasive ductal carcinoma (IDC), invasive breast cancer (IBC), collagen, matrix-assisted laser desorption/ionization–mass spectrometry imaging (MALDI-MSI)

## Abstract

Ductal carcinoma in situ (DCIS) is a heterogeneous breast disease that remains challenging to treat due to its unpredictable progression to invasive breast cancer (IBC). Contemporary literature has become increasingly focused on extracellular matrix (ECM) alterations with breast cancer progression. However, the spatial regulation of the ECM proteome in DCIS has yet to be investigated in relation to IBC. We hypothesized that DCIS and IBC present distinct ECM proteomes that could discriminate between these pathologies. Tissue sections of pure DCIS, mixed DCIS-IBC, or pure IBC (*n* = 22) with detailed pathological annotations were investigated by multiplexed spatial proteomics. Across tissues, 1,005 ECM peptides were detected in pathologically annotated regions and their surrounding extracellular microenvironments. A comparison of DCIS to IBC pathologies demonstrated 43 significantly altered ECM peptides. Notably, eight fibrillar collagen peptides could distinguish with high specificity and sensitivity between DCIS and IBC. Lesion-targeted proteomic imaging revealed heterogeneity of the ECM proteome surrounding individual DCIS lesions. Multiplexed spatial proteomics reported an invasive cancer field effect, in which DCIS lesions in closer proximity to IBC shared a more similar ECM profile to IBC than distal counterparts. Defining the ECM proteomic microenvironment provides novel molecular insights relating to DCIS and IBC.

## 1. Introduction

Ductal carcinoma in situ (DCIS) represents approximately 20% of breast cancers currently diagnosed in US women and is considered a non-obligatory pathway to invasive breast cancer (IBC). The incidence rate of DCIS has seen a relatively recent rise, which is attributed to increased mammographic screening efforts [1,2]. While the expansion in screening has undoubtedly had many positive effects, it has often led to overtreatment of DCIS, since DCIS patients who do not undergo treatment may have between a 14 and 53% risk of developing IBC [1]. Despite the variable risk of disease progression, the standard of care remains local excision for all patients, often combined with radiation therapy and hormone therapy dependent on receptor status. This therapeutic plan is not without risks, including the development of secondary cancers, coronary events, and pulmonary dysfunction [1]. To avoid overtreatment and its complications, improved biological markers are needed to better stratify patients into low- and high-risk categories.

While there has been a continued effort to discover reliable prognosticators, very few have been clinically integrated. Oncotype DX is a 21-gene assay with demonstrated predictive value in IBC [3] that has been adapted into a 12-gene DCIS recurrence score. This recurrence score has some predictive value with low DCIS scores correlating with a lower risk of IBC recurrence [3]. However, this modality necessitates further validation to be integrated widely into clinical practice [4]. Challenges to the development of effective prognosticators in DCIS have included, but are not limited to, the similar copy number alterations and gene expression patterns between DCIS and invasive ductal carcinoma (IDC), an invasive breast cancer type arising within the mammary ducts [5]. Other risk stratification models have focused on clinical characteristics. Namely, a clinical risk score consisting of ER status (a clinically used biomarker) [6], presence of comedo necrosis, and age at diagnosis was found to be associated with increased ipsilateral recurrence risk [7] yet has not been widely adopted due to controversy regarding the prognostic accuracy of recurrence. While histopathological evaluation for features such as high nuclear grade and the architectural pattern of comedo necrosis has demonstrated prognostic potential, consistent assessment of these features has been clinically difficult to achieve [1]. Thus, contemporary efforts have begun to focus on the tumor microenvironment to identify predictive markers in DCIS [8,9].

To expand upon recent work on the DCIS microenvironment, we focused our investigation on the extracellular matrix (ECM) proteome of DCIS, mixed IBC-DCIS, and IBC. It is well-documented that alterations to the ECM occur throughout breast cancer progression, including stiffening and increases in density [10]. Compared to normal breast tissue, invasive and noninvasive breast cancers have increased deposition, thickening, and linearization of collagen fibers with malignant transformation [10]. Various collagen types have been investigated and demonstrated to have increased expression in DCIS by immunohistochemistry [11,12]. In addition to these global alterations in collagen expression, post-translational dysregulation of collagen fibers drives the cellular–matrix interface to influence cell signaling. Prolyl-4-hydroxylases, which hydroxylate proline residues within collagens, have been shown to have increased expression in breast cancer invasion and metastases [13,14], yet sites of proline hydroxylation remain largely unmapped. Peptide-level alterations including differences in post-translational modifications can be spatially explored with high-resolution mass spectrometry imaging [15,16,17,18,19,20]. While collagen is known to have potential as a prognosticator in DCIS [11], our approach is novel in its ability to spatially define multiple peptide alterations to specific pathological regions. Within this study, we used ECM-targeted MALDI-QTOF imaging to further our understanding of the spatial regulation of the collagen proteome in DCIS and IBC.

## 2. Results

### 2.1. Study Overview

The primary aim of this study was to evaluate the spatial regulation of the fibrillar collagen proteome between high nuclear grade DCIS and IBC pathologies. Twenty-two specimens from seventeen patients were annotated by pathologists as DCIS (*n* = 9), IBC (*n* = 4), or containing both DCIS and IBC lesions (*n* = 8) (Table 1, Figure S1, Tables S1–S3). Of the patients with relevant medical history documentation, the median age of the patients was 58.4 years.

**Figure 1:**
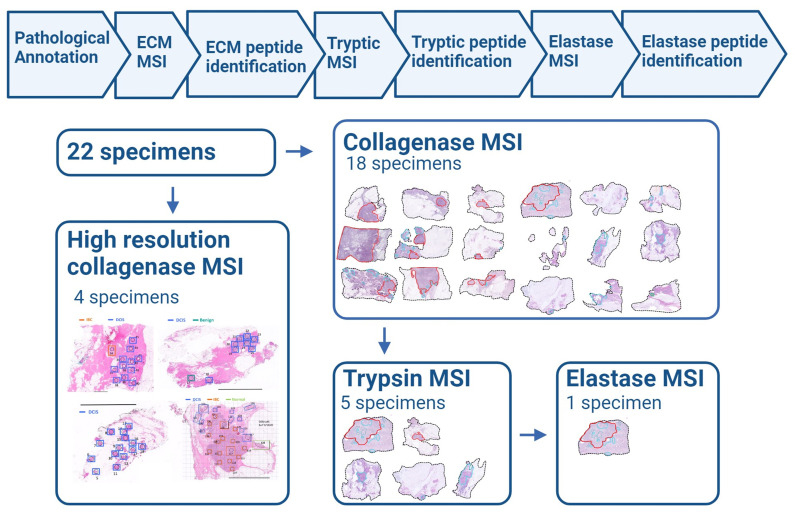
Study workflow. H&E slides were annotated by a breast pathologist with regions defined as DCIS (blue) or IBC (red or orange). On a subsequent tissue section, slides were prepared for collagenase digestion to target the extracellular matrix (ECM). Mass spectrometry imaging (MSI) was performed with matrix-assisted laser desorption/ionization–quadrupole time-of-flight (MALDI-QTOF) imaging. Four samples were annotated per pathological lesion type and underwent the collagenase MSI workflow with high-resolution imaging at an individual lesion level. From the remaining eighteen-sample subset, specific slides were selected for further proteomic analysis multiplexing either tryptic or elastase digestion followed by mass spectrometry imaging. This schema was created in Biorender.com.

A majority of samples were from lumpectomies, with a smaller proportion from mastectomies (Table 1). Tissues were mapped for translational and post-translational collagen regulation using ECM-targeted mass spectrometry imaging across tissue sections to encompass all histopathologic features. A four-sample subset was imaged at high spatial resolutions and used for a detailed investigation of the ECM proteomic profiles of individual DCIS lesions. Proteomic sequencing demonstrated complex variations in the post-translational regulation of fibrillar collagens that could be spatially defined. Additional studies were performed to explore other proteomic features of DCIS pathology using targeted enzymatic approaches coupled to sequencing proteomics (Figure 1). The main finding is that there exists proteomic modulation of fibrillar collagen between DCIS and IBC pathologies, providing strong evidence for larger studies defining the extracellular pathologies related to DCIS.

### 2.2. Spatial Mapping of the Extracellular Proteome Defines DCIS Histopathology

**Figure 2:**
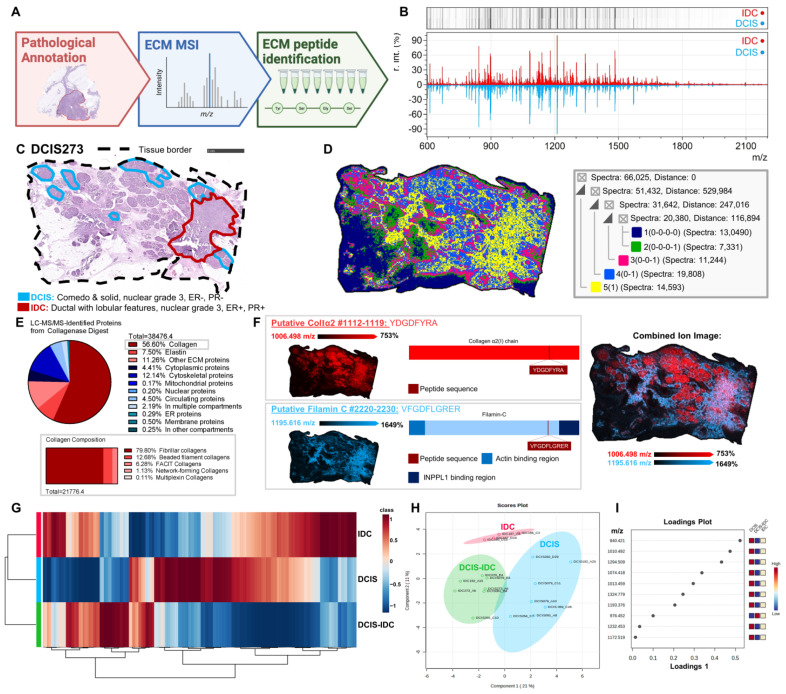
Spatial mapping of the extracellular proteome defines DCIS histopathology. (**A**) The eighteen-sample cohort underwent the workflow depicted, beginning with pathological annotation followed by extracellular matrix (ECM)-targeted mass spectrometry imaging and ECM peptide identification. (**B**) Spectra from pathologist-defined lesions with DCIS shown in blue and IDC shown in red demonstrate different relative peak intensity profiles. R. int. denotes the normalized relative intensity of peaks computed in mMass^®^. (**C**) Hematoxylin and eosin-stained image of a mixed DCIS-IDC specimen demonstrates DCIS (blue) and IDC pathology (red). (**D**) Spatial segmentation analysis was used to define five main proteomic clusters. Cluster 1 (dark blue) annotates to adipocyte regions; Cluster 2 (green) defines borders between adipocyte and stroma; Cluster 3 (pink) localizes to stroma that includes DCIS lesions; Cluster 4 (blue) is localized to stroma and adipocytes primarily between tumor and adjacent tissue; Cluster 5 (yellow) annotates to the cancer region with diminishing detection distant from the tumor. (**E**) Pie chart depicting the proportion of peptide sequences identified from select protein classifications. Collagen fraction is further divided into collagen structural categories. (**F**) Spatial heat maps of a ColIα2 peptide depicted in red show distinct localization to DCIS lesions and surrounding ductal regions compared to the filamin-C peptide, which borders ductal regions and localizes to IDC. INPPL1 denotes inositol polyphosphate phosphatase like 1. Images were normalized to an internal peptide standard. Putative identifications were made by matching imaging data to an ECM database. Numbers following identification indicate the amino acid positions within the entire protein sequence. (**G**) Extracellular matrix peptides distinguished between DCIS, IDC, and DCIS-IDC. Heatmap is the average peptide expression detected across tissue images. (**H**) Sparse Partial Least Squares Discriminant Analysis (sPLS-DA) of pathological regions depicts distinct clustering of regions by specimen classifications of DCIS (*n* = 9), mixed DCIS-IDC (*n* = 6), and IDC (*n* = 4). (**I**) Loadings plot from sPLS-DA depicts the top ten peptide peaks that discriminate between specimen types. Ppm calculations between MALDI-QTOF imaging and LC-MS/MS were within 5 mass accuracy. sPLS-DA and heat map analyses were performed with MetaboAnalyst 5.0.

DCIS pathologies are linked to alterations in collagen organization, which are associated with patient outcomes [9,21,22,23]. However, the discrete regulation of the collagen proteome within its heterogeneous pathologies remains unexplored. To understand the spatial proteomic modulation of collagen domains and associated extracellular proteins in DCIS, targeted spatial proteomics was completed on an eighteen-sample cohort. Collagenase was used to digest extracellular matrix proteins into peptides that were detected for their spatial relationship to tissue pathologies using mass spectrometry imaging (Figure 2A). This targeted approach has the capability to report triple helical collagen domain regulation, which modulates crucial cell and protein interactions that span cellular responses to clinical outcomes [22,24,25,26,27]. Within this eighteen-sample cohort, invasive breast cancer regions were specifically defined as IDC by a pathologist. Spectral comparison of DCIS to IDC regions revealed complex peptide signatures with significantly different intensity profiles (Figure 2B). Heuristic spatial segmentation of 843,210 pixels and 1,005 putatively identified peptide peaks showed 14 primary clusters uniquely localized to histopathological features across the cohort. Largely, clusters defined IDC and DCIS regions, mapped to adjacent stromal tissues, or surrounded a subset of the invasive lesions (Figure S2). An example mixed DCIS-IDC specimen with spatially distinct DCIS and IDC pathological regions had five unique proteomic clusters represented (Figure 2C,D). Notably, proteomic cluster 5 was over-represented in IDC and surrounding stromal regions. To identify the proteomic composition, liquid chromatography–tandem mass spectrometry (LC-MS/MS) was performed. Approximately 56% of peptides identified were collagens with over 79% mapping to fibrillar collagens (Figure 2E, Table S4). Distinct localization of specific peptides to pathological regions was noted. A putatively identified collagen α2(I) sequence showed high-intensity patterns localized within ductal regions, including those containing DCIS lesions. In contrast, another putatively identified peptide from filamin C [28,29] circumscribed stromal regions surrounding ducts and localized within the IDC region (Figure 2F). A comparison of averaged intensity patterns of the putatively identified ECM peptides demonstrated a unique ECM proteomic signature between DCIS, mixed DCIS-IDC, and IDC specimens (Figure 2G). To discern if putatively identified peptide peaks could separate samples by pathology present, a Sparse Partial Least Squares Discriminant Analysis (sPLS-DA) was performed on annotated regions of DCIS or IDC and defined by their specimen classification of DCIS, mixed DCIS-IDC, or IDC. Distinct clustering patterns between the three specimen classifications seemed to suggest pathology-dependent proteomic variations between DCIS, mixed DCIS-IDC, and IDC samples (Figure 2H). A subset of peptide peaks was selected as most predictive in driving these specimen classifications (Figure 2I). This might suggest a distinct extracellular matrix proteome across specimen classifications. In summary, a complex spatially mapped extracellular proteome was defined within DCIS and IDC pathologies. The spatial extracellular proteome was defined predominantly as fibrillar collagens that included post-translationally modified domains.

### 2.3. Fibrillar Collagen Domains Define Pathological Regions of DCIS and IDC

**Figure 3:**
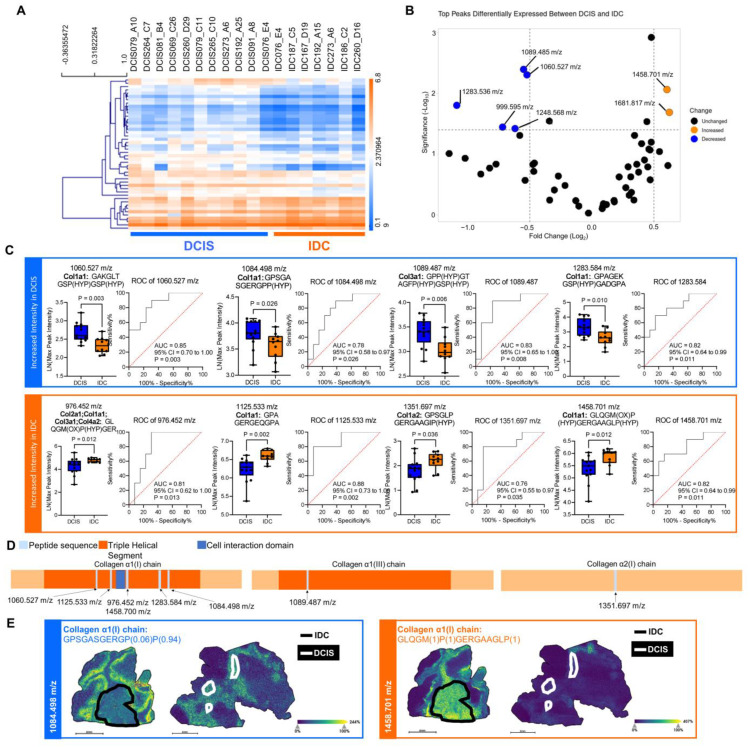
DCIS specimens report distinct fibrillar collagen profiles to pathological regions. (**A**) A total of 43 extracellular matrix peptides were identified across tissue images distinguished between DCIS and IDC by an unpaired, two-tailed *t*-test (*p* < 0.01). (**B**) A volcano plot of peaks identified via LC-MS/MS reports the most significantly differentially expressed peaks between DCIS and IDC pathologies. An absolute value fold change greater than 0.5 between DCIS and IDC with −log(*p*-value) greater than or equal to 1.5 is shown in orange if increased expression was found in IDC and blue if decreased expression was found in IDC. The volcano plot was created with VolcaNoseR. (**C**) Box-and-whiskers plots of fibrillar collagen sequences that are differentially expressed between DCIS (*n* = 13) and IDC (*n* = 10) lesions in eighteen samples by the Mann–Whitney test (*p* < 0.05). ROC analyses of peaks adjacent to box-and-whiskers plots (AUROC > 0.75 and *p* < 0.05 by the Wilson/Brown *t*-test) are shown. Ox denotes oxidation, and HYP denotes hydroxylation of proline residues. (**D**) Location of the identified peptide within the protein sequence found to be differentially expressed by the Mann–Whitney test (*p* < 0.05). (**E**) Spatial heatmaps of MALDI-QTOF imaging of 1084.498 m/z and 1458.700 m/z from two representative samples. Black annotations encircle IDC regions, while white annotations delineate DCIS regions. Ppm calculations between MALDI-QTOF imaging and LC-MS/MS were within 5 mass accuracy.

To investigate specific ECM peptides that might be differentially represented between DCIS and IDC pathologies, the relative intensities of the 1,005 putatively identified peptide peaks between DCIS and IDC lesions were compared. Forty-three putatively identified ECM peptides were found to have significantly different intensity patterns between DCIS and IDC pathologies (Figure 3A). Seven peptide peaks were found to have altered fold changes between DCIS and IDC pathologies (Figure 3B). Of the LC-MS/MS-identified sequences from the 1,005-peptide list, eight collagen peptides were discovered to have significantly different peak intensities comparing DCIS and IDC lesions. Notably, these peptides could discriminate between lesion types (Figure 3C, Table S5) and were identified sequences within fibrillar collagens, specifically collagen α1(I), collagen α2(I), collagen α1(II), collagen α1(III), and collagen α2(V) chains. Given the importance of hydroxylation of proline residues for triple helical stability and its influence on cell function, it was not surprising that many of the differentially expressed collagen sequences contained hydroxylated proline residues and were within the annotated triple helical segment [30] (Figure 3D). Importantly, not all prolines were hydroxylated, and the probability of the modification at each site is shown as a numerical value in parentheses. Certain peptides such as the collagen α1(I) peptide (m/z 1084.498 GPSGASGERGP(0.06)P(0.94)) demonstrated high intensities within DCIS regions, while others including a different collagen α1(I) peptide (m/z 1458.701 GLQGM(1)P(1)GERGAAGLP(1)) exhibited high intensities within IDC regions and adjacent stroma (Figure 3E). Altogether, this suggests that distinct post-translationally modified collagen sequences contained within the triple helical segment can discriminate between DCIS and IDC pathologies.

### 2.4. Extracellular Microenvironment Contributes to Intra-Tumoral Heterogeneity

**Figure 4:**
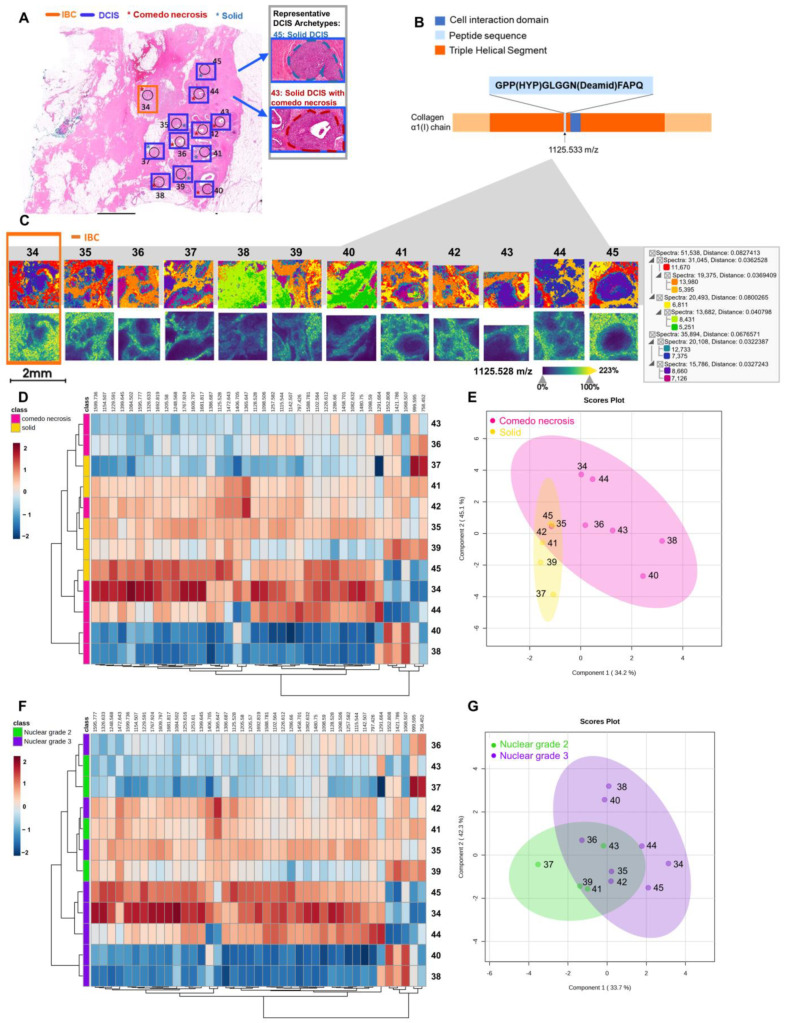
Extracellular microenvironment contributes to intra-tumoral heterogeneity. (**A**) Pathologist-defined lesions are circled in black. Orange delineates an IBC lesion while blue indicates the DCIS lesions. The red asterisk demarcates comedo necrosis and the blue asterisk delineates solid archetypes. For additional information on pathological evaluation, see Supplemental Table S1. (**B**) Peptide sequences are shown within the protein schema. (**C**) The top row represents a segmentation analysis demonstrating proteomic clustering across architectural patterns derived from extracellular matrix-targeted proteomics. Clusters are altered by spatial distance from a discrete invasive cancer site (region 34). The bottom row shows a representative collagen α-1(I) chain peptide detected almost uniformly across the invasive cancer region (34) compared to expression patterns surrounding the DCIS lesions (for example, regions 41 and 45). (**D**) Heatmap of LC-MS/MS identified peptides using Euclidean distance reports differences and similarities across architectural patterns. Pink delineates solid with comedo necrosis and yellow demarcates solid lesions. (**E**) DCIS architectures of comedo necrosis and solid separate based on expression patterns from multiple identified collagen peptides. Multivariate analysis uses sPSL-DA [33]. (**F**) Heatmap of LC-MS/MS identified peptides clustered by Euclidean distance reports hierarchical clustering across nuclear grades. Green denotes nuclear grade 2, and purple defines nuclear grade 3 lesions. (**G**) DCIS lesions characterized by nuclear grade show separation based on the extracellular matrix peptides from the surrounding stroma.

Intra-tumoral heterogeneity is a well-known characteristic of DCIS that contributes to clinical challenges in pathological evaluation for risk assessment of later breast cancer events [31,32]. An initial investigation of proteomic intra-tumoral heterogeneity examined four samples with 59 individual lesions defined by architectural pattern and nuclear grade (Figure 4A and Figures S3–S5, Table S1). Between individual lesions, 41 sequenced peptides linked to the high spatial resolution imaging data were compared for differences in proteomic expression. A spatial segmentation analysis of individual regions of interest demonstrated unique proteomic clusters between regions of interest. Regions that were closer together appeared to share more similar protein clusters than those further away (Figure 4C and Figures S3–S5). An example peptide identified from the collagen α1(I) chain near its cell interaction domain reported differential intensity patterns between regions of interest (Figure 4B,C). Hierarchical clustering of the 41 peptides per lesion demonstrated diversity in ECM proteomic patterns across architecture types (Figure 4D). The sPSL-DA analysis revealed both portions of overlap and distinction between architectural patterns (Figure 4E). Taken together, this seemed to suggest the ECM proteome surrounding individual lesions varied and could not be entirely explained by different archetypes. When stratifying each lesion by nuclear grade, the ECM proteomic profile exhibited some similarity within the same nuclear grade classification (Figure 4F). Moreover, multivariate analysis demonstrated a region of overlap and an area of distinction between nuclear grades 2 and 3 in this specimen (Figure 4G). Altogether, this case demonstrated some variability in expression patterns between lesions of the same nuclear grade and archetype. Analysis of individual DCIS lesions supports the contribution of the extracellular microenvironment to intra-tumoral heterogeneity in DCIS.

### 2.5. Distinct Tryptic Peptide Profiles Define Pathological Regions

**Figure 5:**
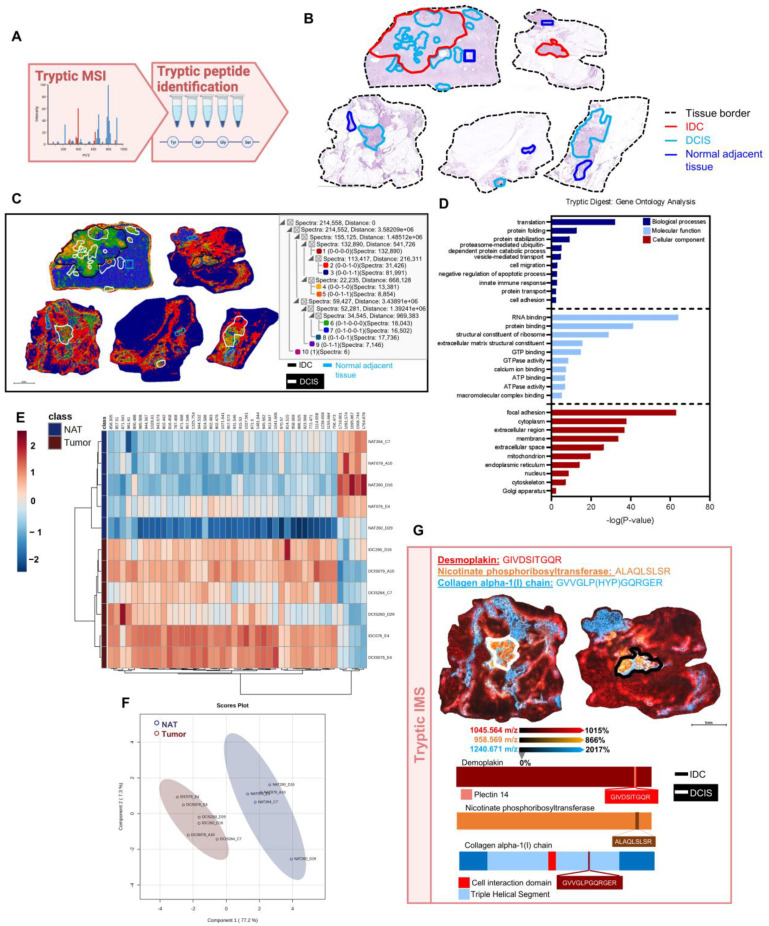
Distinct tryptic peptide profile defines pathological regions. (**A**) Workflow for tryptic digestion depicted. This schema was created in Biorender.com. (**B**) Hematoxylin and eosin-stained images showing normal adjacent tissue and pathological annotations. (**C**) A segmentation analysis of tryptic peptides from 5 specimens of 214,558 pixels and 1104 peaks demonstrates 10 uniquely localized proteomic clusters. (**D**) Top ten significant GO terms associated with differentially expressed peptides associated with cellular components (red), molecular functions (light blue), and biological processes (dark blue). (**E**) Differential expression patterns of tryptic peptides among normal adjacent tissue (NAT; *n* = 5) and tumors (*n* = 6) by a two-tailed *t*-test (*p* < 0.01). Tryptic digest targets both cellular and extracellular components. (**F**) Normal adjacent tissue and tumor separate based on sPLS-DA analysis of tryptic peptides. (**G**) Spatial heatmaps of 3 tryptic peptide peaks depict discrete localization to IDC, DCIS, and surrounding normal adjacent tissue.

To further test the potential for multi-omic studies in assessing DCIS, we increased our investigation of the proteomic niche of DCIS and IDC using trypsin, which provides untargeted, primarily cellular proteomic information. After the collagenase data collection described previously, a tryptic digest was performed on five samples within the eighteen-sample cohort (Figure 5A,B). A segmentation analysis of 214,558 spectra and 1,104 LC-MS/MS-identified tryptic peaks revealed distinctly localized proteomic clusters. DCIS076 was found to be the most distinct specimen containing proteomic clusters not represented within the other samples. Additionally, discrete proteomic groups spatially overlaid ductal compartments within pathologically annotated regions and localized to the adjacent stroma (Figure 5C). To further understand the tryptic proteomic profiles of these samples, a gene ontology (GO) analysis was performed on our LC-MS/MS-identified proteins (Figure 5D, Table S6). Notably, many extracellular matrix terms were identified within top-ranking categories, highlighting the importance of extracellular matrix alterations in DCIS and IBC previously described in the literature [8,9,21]. To assess for pathology-specific proteomic variations, a comparison of tryptic peptide intensity profiles between pathological regions and normal adjacent ductal regions was performed and reported 47 peptides with significantly different intensities (Figure 5E, Table S7). An sPLS-DA analysis demonstrated a distinct clustering of tumor lesions and normal adjacent ductal regions (Figure 5F). Specific peaks such as m/z 1045.564 identified as a desmoplakin peptide were spatially distributed outside the pathological annotations and within the adjacent microenvironment. Others such as m/z 958.566, a peptide from nicotinate phosphoribosyltransferase, primarily localized to the cellular compartments, whereas m/z 1240.671, a collagen α-1(I) chain peptide from the triple helical segment, surrounded cellularly dense regions and localized to adjacent stromal tissues (Figure 5G and Figure S6). It is interesting to note that both desmoplakin, an important constituent of desmosomes [34], and nicotinate phosphoribosyltransferase, involved in NAD^+^ biosynthesis [35], have been linked to breast cancer. Overall, the data demonstrate pathology-dependent proteomic alterations within the surrounding ECM and cellular compartments.

### 2.6. Serial Enzymatic Digest Reveals Pathology-Specific Proteomes and Proteomic Field Cancerization

**Figure 6:**
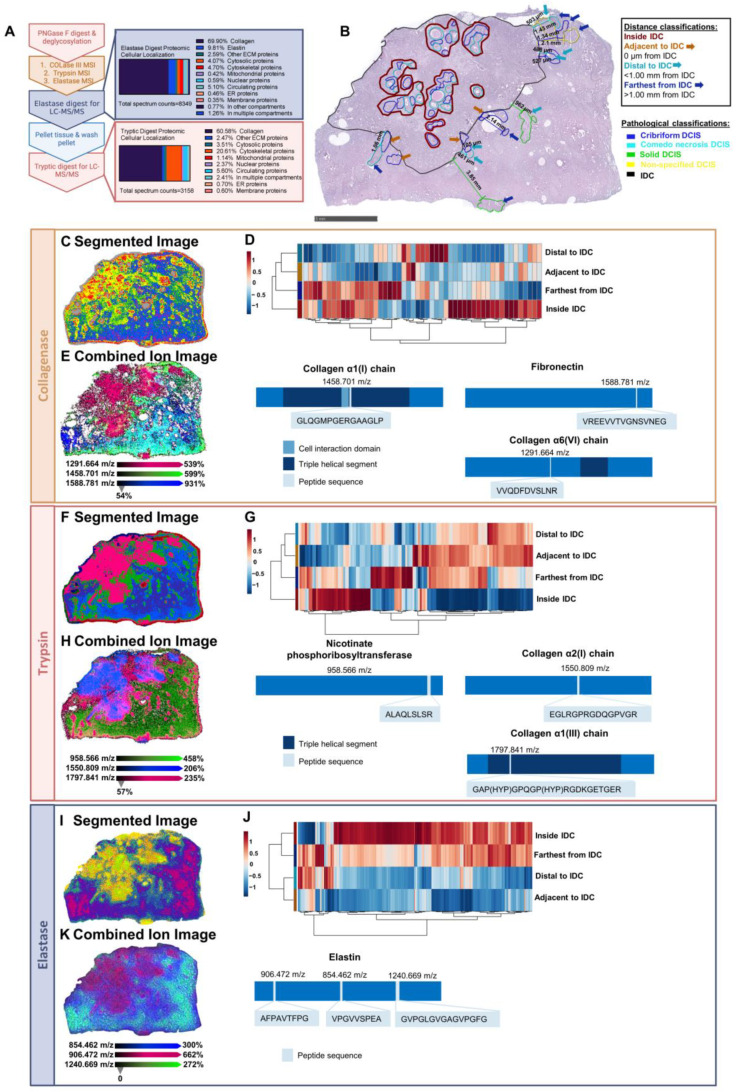
Serial enzymatic digest reveals pathology-specific proteomes and proteomic field cancerization. (**A**) Workflow for serial enzymatic digestion depicted with cellular localization from LC-MS/MS proteomic hits from each enzymatic digestion shown. Tissue was digested by collagenase to define stroma composition, trypsin to capture cellular features and additional extracellular composition, and elastase to target elastin. (**B**) Pathologist-defined lesions annotated by architectural pattern and distance to the invasive cancer site. (**C**) Segmentation analysis from 53 peptides derived from stroma. The tumor (yellow) and adjacent tissue (blue) represent distinct clusters with stromal composition extruding from the tumor (green). (**D**) DCIS lesions show distinct stromal signatures dependent on distance from IDC. (**E**) Spatial heatmaps of 3 collagenase peptide peaks (1291.664 m/z, 1458.701 m/z, 1588.781 m/z) depict discrete localization to IDC, DCIS, and surrounding normal adjacent tissue. Peptide sequences depicted within protein schemas. (**F**) Segmentation from serial tryptic digestion highlights the invasive cancer field (pink) and normal adjacent tissue (blue). Potential margins and punctate extensions form a unique cluster (green). (**G**) Differential expression detected by tryptic peptides based on location relative to IDC. (**H**) Spatial heatmaps of 3 tryptic peptide peaks (958.566 m/z, 1797.841 m/z, 1550.809 m/z) depict discrete localization to IDC, DCIS, and surrounding normal adjacent tissue. Peptide sequences depicted within protein schemas. (**I**) Segmentation analysis of peptides derived from elastase digestion. The tumor field (yellow and green) extends further into the normal adjacent tissue (blue and purple) compared to tryptic segmentation profiles. (**J**) DCIS lesions show differential signatures derived from 393 peptides produced by elastase digestion dependent on distance from IDC. (**K**) Spatial heatmaps of 3 elastase peptide peaks (906.472 m/z, 854.462 m/z, 1240.669 m/z) identified from an elastase-digested peptide library depict discrete localization to IDC, DCIS, and surrounding normal adjacent tissue. Peptide sequences depicted within protein schemas. Ppm calculations between MALDI-QTOF imaging and LC-MS/MS were within 5 mass accuracy.

Field cancerization is defined as a similarity of molecular alterations between carcinomas and their surrounding tissues. In both DCIS and IBC, cancer field effects are thought to contribute to local recurrence following surgical resection [36]. While field cancerization at the epigenetic and genetic levels has been well-studied [37,38,39], little is understood about proteomic modulation of the IBC field on adjacent DCIS regions. Given its lesion heterogeneity, DCIS076 was selected as a case study to explore architectural patterns and distance from IDC’s influence on the DCIS proteomic niche. Serial enzymatic digestion coupled with MALDI-QTOF imaging was performed to capture an expanded spatial view of the DCIS-IDC proteome (Figure 6A, Tables S6 and S8). DCIS lesions were divided into categories based on distance from the IDC region: DCIS regions within IDC, DCIS regions adjacent to IDC (0 µm from the invasive border), DCIS regions distal to the IDC (over 0 µm but within 1.0 mm of the invasive region), and DCIS regions farthest from IDC (greater than 1.0 mm from the invasive region) (Figure 6B). Individual lesions were defined as solid, cribriform, or comedo necrosis architectural patterns. A segmentation analysis of collagenase-digested peptides reported proteomic clusters localized to IDC- and DCIS-annotated regions (Figure 6C and Figure S7). Average intensity patterns of 53 identified ECM peptides from the collagenase digest revealed differences between distance classifications with DCIS lesions in the IDC region reporting the most distinct ECM signature by hierarchical clustering (Figure 6D). The sPLS-DA plot demonstrated a similar trend with DCIS lesions inside the IDC region displaying the least amount of overlap with lesions of other distance classifications (Figure S7). Spatial localization demonstrated increased intensities of certain peaks within the IDC region, such as m/z 1291.664, which corresponded to a collagen α6(VI) chain peptide located within the non-triple helical region [30]. Interestingly, gene expression of the collagen α6(VI) chain has been reported to be upregulated in the triple-negative primary tumors compared to the axillary lymph node metastases, which could suggest its importance within the primary tumor microenvironment [40]. Other peaks showed increased intensities outside the IDC region such as m/z 1588.781, a fibronectin peptide within domain 17 [30], and m/z 1458.701, corresponding to a collagen α1(I) chain peptide near the cell interaction domain [24] (Figure 6E, Figure S8).

To capture the cellular proteomic niche, a tryptic digest was then performed on DCIS076. A segmentation analysis of 128 putatively identified tryptic peptide peaks similarly reported distinct proteomic clusters localized to DCIS and IDC regions (Figure 6F and Figure S6). As with the collagenase proteomic profiles, average intensity patterns of tryptic peptides demonstrated that the DCIS lesions within the IDC region had a unique proteome when compared to the DCIS lesions of other distance classifications (Figure 6G). Specific tryptic peptides such as m/z 955.566, identified from nicotinate phosphoribosyltransferase, exhibited high-intensity profiles within the IDC and DCIS regions. While other tryptic peptides such as m/z 1550.809 from the collagen α2(I) chain and m/z 1797.841 from the collagen α1(III) chain were localized primarily to regions outside the DCIS and IDC lesions (Figure 6H and Figure S9). In contemporary literature, a transcriptional signature including *COL1A2* was associated with reduced overall survival in breast cancer [41]. Similarly, *COL3A1* has been reported to have an important role in breast cancer progression as knockdown studies in triple-negative breast cancer cell lines have been linked to reduced invasion and proliferation [42]. Taken together, these data support a proteomic field effect from the invasive breast cancer region.

Given the significance of the extracellular matrix and associated proteins within our tryptic digest, a subsequent elastase digest was performed to target elastin, which has been associated with breast cancer invasion [43]. The elastase digest of DCIS076 primarily targeted ECM-associated proteins with collagens and elastin reported as the top proteomic hits. A segmentation analysis of LC-MS/MS-identified elastin and elastin-associated peptides reported distinct clusters within pathologically annotated regions and others in surrounding adjacent regions (Figure 6I and Figure S6). Average intensity patterns between distance classifications reported proteomic variations between DCIS lesions of varying distances from the IDC region (Figure 6J and Figure S1). A spatial investigation of elastin peptides demonstrated distinct peaks with increased intensity profiles within the IDC region and DCIS lesions such as m/z 906.472, while peaks such as m/z 1240.669 and m/z 854.462 reported increased relative intensity patterns outside IDC and DCIS lesions (Figure 6K). Furthermore, these data highlight the utility of serial enzymatic digestion to expand upon the number of peptides with distinct intensity patterns between pathologies and enhance the characterization of the DCIS proteomic niche. Importantly, these proteomic findings are supported by contemporary literature in the breast cancer field. DCIS lesions within the invasive cancer field have a distinct proteomic signature, which suggests that the spatial regulation of the DCIS proteome is influenced by the invasive breast cancer field.

## 3. Discussion

Transitions from benign to invasive breast cancer are earmarked with progressive changes in the structure and composition of stroma, with limited reports of how proteomic alteration contributes to the pathology of the breast microenvironment. This study establishes that dynamic collagen proteomic regulation occurs throughout the breast tissue microenvironment. The use of a collagen-targeting proteomic imaging method applicable to clinically archived tissue provided novel insight into the preinvasive breast microenvironment and transitions to invasive cancer. A major finding was that distinct collagen proteomic profiles could distinguish DCIS, DCIS-IDC, and IDC. Intriguingly, multiplexing both cellular and extracellular proteomic approaches reported a field cancerization effect that demarked tumor site localization with gradients extending to select DCIS lesions outside of the primary tumor site. The field effect included a contrasting proteome that differentiated adjacent normal ductal tissue. Spatially powered proteomic analysis further reported that heterogeneity exists within the regional microenvironment of DCIS lesions, defining the boundaries of lesion pathology. While lesion heterogeneity at the genomic level has been demonstrated [44], the current study offers new insights into the proteomic composition of the local ECM microenvironment at an individual lesion level. The spatially driven multiplexed approach supported that phenotypic heterogeneity in nuclear grade and archetype [45] extends to the localized proteome and may provide a molecular differentiator for noninvasive to invasive cancer pathologies.

The current literature depicts collagen fiber regulation as a distinguishing signature within the breast microenvironment predictive of recurrent DCIS [46], prognostic of early breast cancer [21,47], and altering with progressive breast cancer [48]. This study found that specific fibrillar collagen domains that included post-translational modifications showed altered intensity distribution between DCIS and IDC. These collagen domains presented as strong single classifiers that differentiated DCIS from IDC. This supports previous work by antibody staining that fibrillar collagens differentiate the tumor microenvironment in DCIS and IDC [8,9]. The current study advanced this concept to report amino acid sequences of the regulated domains distinguishing DCIS from IDC. Notably, many of the collagen sequences that differentiated DCIS and IDC included the post-translational modification of proline hydroxylation. Proline hydroxylation was reported at specific residues within the collagen sequences, highlighting that the breast microenvironment is marked by dynamic, yet site-specific post-translational regulation of collagen structure. Collagen hydroxylated proline residues constitute cell binding domains [24,25], previously linked to the regulation of tumor dormancy [49] and controlling immune cell localization [50]. Prolyl-4-hydroxylase, the enzyme that primarily hydroxylates proline residues within collagen, modifies tumor progression [14,51], is essential for metastasis [52], and is linked to poor survival outcomes in breast cancer [53]. We pose that site-specific collagen hydroxylation of the DCIS microenvironment is an important priming component for the evolvement of IBC and represents a potential breast cancer prognosticator. Further incorporation of ECM peptides into a classification algorithm may lead to a novel model for DCIS risk stratification or could improve existing classification systems. Integration with clinically utilized biomarkers such as estrogen receptor status or pathological grading could strengthen the predictive value of these identified ECM peptides.

Cancer field effects were observed in the breast collagen proteome. Cancer field effects or cancerization is a paradigm by which a normal cell can acquire pro-tumorigenic features and influence surrounding areas, or fields, to promote cancer [54], a concept that inherently involves the extracellular microenvironment. Field cancerization is considered relative to cancer evolution, whereby cancer cells acquire mutations that allow them to adapt to the microenvironment [55]. In the current study, collagen proteome gradients were observed surrounding the tumor and extending to certain DCIS lesion sites with some distance dependence. Thus, the surrounding proteomic microenvironment appears to have a significant role that at minimum could form a connective pathway of chemical biology between invasive cancer sites and DCIS. Further, the lesion-specific investigation showed common proteomic signatures between cancer sites and only certain DCIS lesions, suggestive of differing cellular origins for field effects. This is supported by literature showing that up to 75% of DCIS lesions are true invasive cancer precursors and up to 18% of invasive cancers arise from independent lineages [56]. Although much of the focus in cancer field effects and cancer evolution is on cellular morphology and genetic mutations, it is unknown how the extracellular microenvironment contributes to the promotion and emergence of breast cancer. It is likely that maladaptation of the extracellular microenvironment results in aberrant chemical gradients producing cancerous field effects with mismatched cell interfaces that stabilize mutational adaption, allowing cancer evolution. This is hypothesized to be a compounding feed-forward effect, under current investigation by the described proteomic approaches in larger cohorts.

This foundational study supports that DCIS lesion pathologies are marked by heterogeneity that includes unique collagen proteomic variation. In a patient-specific tissue with highly localized cancer sites, it was expected that comedo necrosis lesions, associated with invasive cancer risk [57], would show similar patterns to the invasive cancer site. However, only certain comedo necrosis lesions clustered with collagen signatures from the invasive cancer site. Solid pathologies in the same patient often clustered with nearby comedo necrosis, further implicating an underlying proteomic field effect. Additionally, primarily high nuclear grade DCIS pathologies, considered at increased risk for progression to IDC [58], were investigated. Within high nuclear grade pathologies, a significant heterogeneity of the collagen proteome was also observed. While lesion heterogeneity is increasingly being viewed as a pathological feature of DCIS [45], it remains unclear how signatures contribute to emergent invasive cancer. Further investigations multiplexing the spatial proteome as shown by this study and expanding on intra- and inter-patient DCIS lesions are expected to provide insight into the origins of heterogeneity.

There were limitations of this study. Sample size was limited. This study sought to build foundational examples to understand the potential of multiplexed spatial omics, and larger, highly annotated cohorts must be analyzed with these approaches to build a comprehensive portrait of proteomic changes in DCIS and IDC. It is also important to note that genetic ancestry plays a role in disparities in progression to IDC [59] that have yet to be investigated. To develop predictive biomarkers for recurrence, DCIS pathologies must be studied at primary diagnosis and linked to outcomes as well as ancestry data.

## 4. Materials and Methods

### 4.1. Materials

Xylenes and HPLC grade water were purchased from Fisher Scientific (Hampton, NH, USA). Trifluoroacetic acid, ethanol, acetonitrile, and α-cyano-4-hydroxycinnamic acid were purchased from Sigma Aldrich (St. Louis, MO, USA). Collagenase type III (COLase3) was procured from Worthington Biochemical (Lakewood, NJ, USA) and PNGase F PRIME™ was obtained from N-Zyme Scientifics (Doylestown, PA, USA). Trypsin was ordered from Sigma Aldrich (St. Louis, MO, USA) and elastase was purchased from Alfa Aesar (Haverhill, MA, USA).

### 4.2. Patient Cohort

Eighteen breast tissue samples were obtained from the Department of Surgery at Duke University. Samples were scored as nuclear grade 2 or 3 by a pathologist. The average age of diagnosis of patients within the cohort was 58.4 years old (SD = 13.6). Samples were annotated by a pathologist as DCIS only, DCIS and IDC, and IDC only according to the College of American Pathologists “Protocol for the Examination of Specimens From Patients With Ductal Carcinoma In Situ (DCIS) of the Breast” [60]. Architectural patterns of specimens were classified as solid, cribriform, or comedo necrosis (Table S1). An additional four specimens were acquired from the Department of Surgery at Duke University for higher-resolution imaging of individual pathological lesions. Architectural patterns of DCIS lesions were defined as solid, cribriform, comedo necrosis, or a combination of patterns. Nuclear grade was assigned to each region of interest (Table S1). The type of invasive breast cancer was not specified in this four-sample subset and is referred to as invasive breast cancer (IBC).

### 4.3. Histological Staining

Formalin-fixed paraffin-embedded slides were obtained directly from collaborators and stained with hematoxylin (Gill 2) and eosin-y (Fisher Scientific, Hampton, NH, USA) using the manufacturer’s instructions. Each slide was then imaged on a high-resolution scanner (Nanozoomer, Hamamatsu, Japan) to obtain a whole tissue image.

### 4.4. Matrix-Associated Laser Desorption/Ionization–Mass Spectrometry Imaging (MALDI-MSI) FFPE Tissue Preparation

Prior to use, samples were de-glycosylated with PNGase F PRIME enzyme using methods previously established [61,62,63]. To improve COLase3 access, N-glycans were then removed prior to its application [15]. Samples were processed using a previously established protocol [18].

Antigen retrieval was performed in 10 mM Tris HCL at pH 9 for 20 min in a Decloaker chamber for samples at 95 °C. COLase3, elastase, or trypsin was applied to slides using a M3 or M5 TM-Sprayer Tissue MALDI Sample Preparation System (HTX Technologies, LLC, Chapel Hill, NC, USA) with the following settings: 40 °C, 10 psi, 25 µL/min, 1200 velocity, and 15 passes. Following a 5 h incubation at 37 °C at ≥80% humidity, tissues were sprayed with a MALDI matrix consisting of 7 mg/mL α-cyano-4-hydroxycinnamic acid dissolved in 50% acetonitrile/1% trifluoracetic acid with 0.15 picomoles Glu-1-Fibrinopeptide-1 as an internal standard. Matrix was sprayed at 79 °C, 10 psi, 70 µL/min, and 1300 velocity for a total of 14 passes. Following matrix application, slides were quickly immersed in cold 5 mM ammonium phosphate monobasic and allowed to dry in a desiccator prior to imaging.

### 4.5. MALDI-MSI

A timsTOF fleX imaging mass spectrometer (Bruker, Bremen, Germany) with matrix-assisted laser desorption/ionization (MALDI) capabilities was used to analyze tissue sections. Images were acquired in positive ion mode within an m/z range of 700–2500. The laser was set to fire 300 shots per pixel with 60–80 µM between each pixel for the eighteen-sample cohort and with 20–40 µM between each pixel for the higher resolution studies. Transfer time was 75.0 µs and pre-pulse storage was 20.0 µs.

FlexImaging v. 7 and SCiLs Lab software 2023c Pro (Bruker Scientific, LLC, Bremen, Germany) were utilized to visualize and analyze the data. Collagenase peptide spectral data were normalized to the root square mean for the eighteen-sample cohort unless otherwise specified and to an internal peptide standard for the higher-resolution imaging of the four-sample cohort. Tryptic and elastase peptide spectral data were normalized to the peptide internal standard unless otherwise specified. Spectral data were manually analyzed to putatively identify ECM peptide peaks especially those that were spatially expressed in regions of pathology. We focused our analysis on the mean spectrum statistics of maximum peak intensity with the internal processing mode of peak maximum with the peak interval width set to ±20 ppm. Segmentation analysis was performed in SCiLs software 2023c Pro using the k-bisecting method with the Manhattan metric. Prior to analysis, peak intensities were transformed using the natural logarithm.

### 4.6. Sample Preparation for LC-MS/MS Proteomics

Following MALDI-TOF MSI, samples were stained with hematoxylin and eosin to confirm the localization of pathological regions from annotations completed on another tissue section. Tissue sections were de-stained with a series of xylene and ethanol washes with Carnoy’s solutions interspersed [20]. A razor blade was used to perform a macro-dissection of a selected subset of the slides to obtain four samples with primarily DCIS lesions and four with primarily IBC. For the COLase3 workflow, samples were placed in Eppendorf tubes and underwent COLase3 digestion overnight at 38 °C and 450 rpm [15]. The following day, samples were sonicated and underwent a second COLase3 digestion for 5 h to increase the abundance of peptides. For tryptic and elastase workflows, samples only underwent a 5 h enzymatic digestion. With regards to DCIS076, which underwent an elastase digest followed by a tryptic digest, the sample was pelleted and washed extensively prior to the tryptic digest. To remove undigested proteins, enzyme, and salts, a C18 StageTip (Thermo Fisher Scientific, Waltham, MA, USA) was used, followed by a ZipTip (Millipore Sigma, Burlington, MA, USA) before loading samples on the column.

### 4.7. LC-MS/MS Peptide Sequencing

Peptide sequencing information was acquired using an EASY nanoLC 1200 system (Thermo Fisher Scientific, Waltham, MA, USA) coupled to an Orbitrap Exploris 480 mass spectrometer (Thermo Fisher Scientific, Waltham, MA, USA). Prior to chromatographic separation, two µg of peptide was resuspended in solvent A (5% acetonitrile, 0.1% formic acid). Peptides were loaded onto a C18 reversed-phase column (Acclaim™ PepMap™ RSLC, 75 µm × 25 cm (2 µm, 100 Å)) with increasing solvent B (80% acetonitrile, 0.1% formic acid) from 0 to 35% over a 180 min gradient for collagenase digests and 170 min gradient for tryptic/elastase digests. Samples were run at a flow rate of 300 nL/min. The Orbitrap was used to acquire MS1 data (60,000 resolution; maximum injection time: 25 ms; normalized AGC target: 300%). For collagenase MS2 data, charge states between 2 and 7 with a dynamic exclusion window of 20 s were analyzed. For tryptic and elastase MS2 data, charge states between 1 and 7 with a dynamic exclusion window of 20 s and cycle time of 3 s were analyzed. The ion trap with HCD fragmentation (isolation window: 1.4–2 m/z; collision energy: 33%; maximum injection time: 40 ms; normalized AGC target: 100%) was used for MS2 scans. Thermo Scientific Xcalibur 4.5 software was utilized for data recording. MaxQuant was used for database searching for peptide identifications. Peptides were filtered out if they had scores less than 70 assigned in MaxQuant. Probabilities of a post-translational modification at a site are denoted by a numerical value between zero and one surrounded by parentheses. For putative peptide identifications, mass spectrometry images were matched to peaks within 5 ppm mass accuracy of previously identified extracellular matrix peptides from previously published databases [18,20].

### 4.8. Proteomic Analysis

MSFragger v. 20.0 was used for peptide identification for protein-level classifications with a false discovery rate of 0.01. Database search results from MSFragger were uploaded into Scaffold v. 5.3.0 for quantification and total spectrum counts. For collagenase digests, results were filtered by a protein threshold of 99.9% [64], a minimum of 3 peptides, and a peptide threshold of 98% [65]. For tryptic and elastase digests of DCIS076, the protein threshold was set to 99.0% [64], a minimum of 2 peptides, and a peptide threshold of 99.0% [65].

### 4.9. Statistics

Data are summarized graphically and numerically for exploratory data analysis using descriptive statistics (e.g., mean, standard deviation, frequency, and relative frequency). Heatmaps and clustering analyses were performed through MetaboAnalyst 5.0, ClustVis, or Multiple Experiment Viewer. The natural logarithms of each peak intensity were imported into MetaboAnalyst 5.0 [66]. Multiple Experiment Viewer was used to create a Pearson correlation heatmap distinguishing between DCIS and IDC pathologies. Otherwise, Euclidean distance was used to produce heatmaps with clustering by the Ward method performed in ClusVis or MetaboAnalyst 5.0. A Sparse Partial Least Squares Discriminant Analysis (sPLS-DA) was performed to discern if peptide peaks could classify specimens based on different pathological features including lesion architectural types [33]. The VolcaNoseR web tool was utilized to generate the volcano plot comparing the relative intensities of peptide peaks between DCIS and IDC pathologies [67]. Box plots and Receiver-Operator Curves (ROCs) were generated using GraphPad Prism 10.0.2. Mann–Whitney tests (*p* < 0.05) were utilized to assess the significance of box plots while Wilson/Brown *t*-tests (*p* < 0.05) were used for ROC analysis.

### 4.10. Gene Ontology (GO) Analysis

The Database for Annotation, Visualization, and Integrated Discovery (DAVID) was utilized for functional analysis of tryptic proteomic data. Proteomic hits were imported into DAVID. Then, cellular component, molecular function, and biological processes analyses were completed. Pathways were assessed for redundancy of proteomic hits and the top ten descriptive terms with the least redundancy were reported.

## 5. Conclusions

DCIS is a noninvasive breast disease with the potential to progress to invasive cancer, resulting in a significant amount of overtreatment. New biomarkers are needed that can report lesions that are likely to progress to invasive cancer; identification of such markers will greatly improve patient management. Breast stroma forms the basis for clinical care throughout breast health. The current study reports that signatures from multiplexed proteomic imaging approaches can differentiate breast pathologies. This study highlights the potential for the collagen proteome to distinguish between DCIS and IDC. The data support that field cancerization is observed in the underlying extracellular proteome within the breast microenvironment and provide novel insight into breast heterogeneity. Overall, spatial, multiplexed proteomic analysis of the breast stroma microenvironment presents significant utility in understanding breast biology throughout breast health. The collagen proteome presents a high potential for clinical utility in differentiating breast pathologies and may be a novel avenue for markers that improve patient care.

## Figures and Tables

**Table 1:** Summary of patient characteristics. A total of 13 patients were used for the first part of the study for ECM-targeted mass spectrometry imaging. Five patients had two specimens used. For a full list of the samples used, see Supplemental Figure S1. Within this table, patients were stratified according to pathology. Architecture patterns were determined by at least one pathologist. SD denotes standard deviation. NA delineates not applicable. * indicates that some DCIS tumor sizes were characterized as percentages or qualitatively (see Supplemental Tables for more information).

Age of Dx, Mean (SD)	*n* = 13 Patients	
	58.4 (SD = 13.6)	
Pathology, *n* (%)	*n* = 18 specimens	
DCIS only	7 (38.9%)	
Mixed DCIS-IDC	6 (33.3%)	
IDC	4 (22.2%)	
Inflammatory foci	1 (5.6%)	
Surgical Treatment, *n* (%)	*n* = 11 patients	
Lumpectomy	5 (45.5%)	
Partial mastectomy	3 (27.3%)	
Mastectomy	3 (27.3%)	
Race, *n* (%)	*n* = 6 patients	
African American	1 (16.7%)	
White	5 (83.3%)	
	DCIS	IDC
Nuclear Grade, *n* (%)		
1	0 (0%)	0 (0%)
2	3 (23.1%)	2 (20%)
3	10 (76.9%)	8 (80%)
Architecture, *n* (%)	Note some DCIS samples have mixed pathology	
Solid	11 (84.6%)	NA
Cribriform	3 (23.1%)	NA
Comedo necrosis	6 (46.2%)	NA
Micropapillary	1 (7.7%)	NA
Pathological tumor size (cm), Mean (SD)	*n* = 4 *	*n* = 13
	5.4 (SD = 2.9)	3.3 (SD = 2.6)
Marker Status, *n* (%)	*n* = 8	*n* = 13
ER(+)	3 (37.5%)	7 (53.8%)
PR(+)	2 (25.0%)	7 (53.8%)
HER2(+)	3 (37.5%)	7 (53.8%)

## Data Availability

The original data presented in the study are openly available in ProteomeXchange by logging into the MassIVE FTP server with this URL: ftp://MSV000094690@massive.ucsd.edu and the username: MSV000094690.

## Supplementary Materials

The following supporting information can be downloaded at: https://www.mdpi.com/article/10.3390/ijms25126748/s1.

## Abbreviations

DCIS	Ductal carcinoma in situ
HYP	Hydroxylated proline
IBC	Invasive breast cancer
IDC	Invasive ductal carcinoma
LC-MS/MS	Liquid chromatography tandem mass spectrometry
MALDI-MSI	Matrix-Assisted Laser Desorption/Ionization–Mass Spectrometry Imaging
MALDI-QTOF	Matrix-Assisted Laser Desorption/Ionization–Quadruple Time-of-Flight
OX	Oxidation
PPM	Parts per million
sPLS-DA	Sparse Partial Least Squares Discriminant Analysis
