# Supplementary material for: Extracellular Microenvironment Alterations in Ductal Carcinoma In Situ and Invasive Breast Cancer Pathologies by Multiplexed Spatial Proteomics
Source: Int J Mol Sci. 2024 Jun 19;25(12):6748. doi: 10.3390/ijms25126748 (PMC11203487; doi:10.3390/ijms25126748)
Supplement: Supplementary file 1 [file ijms-25-06748-s001.zip › ijms-3024210-supplementary.pdf]

Supplemental Figures for "Extracellular Microenvironment Alterations in Ductal Carcinoma In Situ and Invasive Breast Cancer Pathologies by Multiplexed Spatial Proteomics"

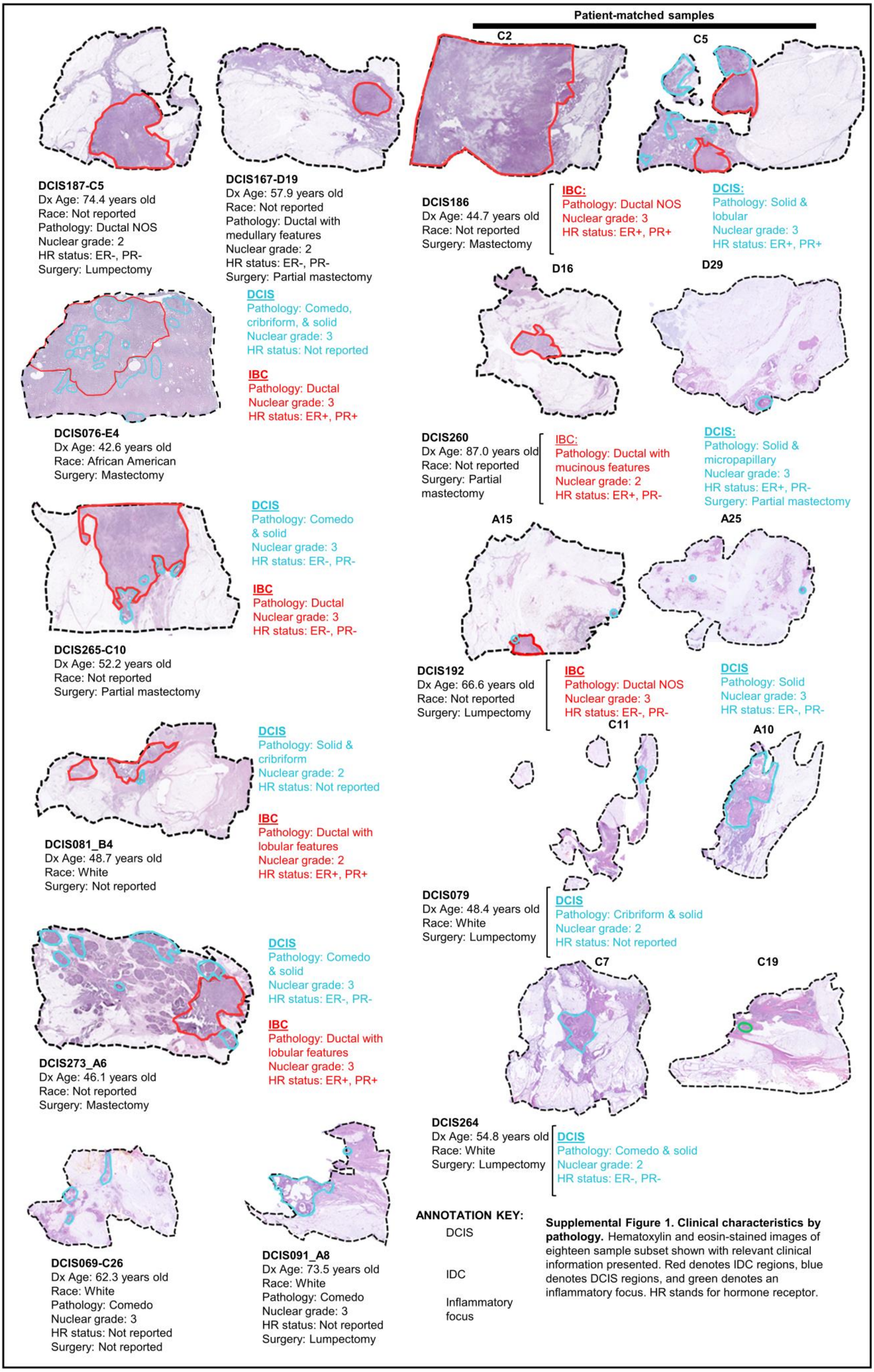

Supplemental Figure 1. Clinical characteristics by pathology. Hematoxylin and eosin-stained images of eighteen sample subset shown with relevant clinical information presented. Red denotes IDC regions, blue denotes DCIS regions, and green denotes an inflammatory focus. HR stands for hormone receptor.

Supplemental Table S1. High Resolution Cohort. Regions of interests (ROIs) with nuclear grade and architectural pattern summarized. NA denotes not applicable.

ROI #	Histology	Slide	DCIS Grade	DCIS Type	DCIS Necrosis	Invasive Nuclear Grade	Invasive Architectural Grade
5	DCIS	DCIS003_B5	2	solid	no	NA	NA
6	DCIS	DCIS003_B5	2	solid	no	NA	NA
7	DCIS	DCIS003_B5	3	solid	no	NA	NA
8	DCIS	DCIS003_B5	3	solid, comedo	comedo	NA	NA
9	DCIS	DCIS003_B5	3	solid	no	NA	NA
10	DCIS	DCIS003_B5	2	solid	comedo	NA	NA
11	DCIS	DCIS003_B5	3	solid, cribriform	focal	NA	NA
12	DCIS	DCIS003_B5	2	solid	no	NA	NA
13	DCIS	DCIS003_B5	2	solid	comedo	NA	NA
14	DCIS	DCIS003_B5	2	solid	focal	NA	NA
15	DCIS	DCIS003_B5	3	solid	focal	NA	NA
16	DCIS	DCIS003_B5	3	solid	focal	NA	NA
17	DCIS	DCIS003_B5	3	solid	no	NA	NA
18	DCIS	DCIS003_B5	2	solid	focal	NA	NA
17	Non-Atypical	DCIS024_A29	NA	NA	NA	NA	NA
19	DCIS	DCIS024_A29	2	cribriform	no	NA	NA
20	DCIS	DCIS024_A29	2	cribriform	no	NA	NA
21	DCIS	DCIS024_A29	2	cribriform	focal	NA	NA
22	DCIS	DCIS024_A29	1	cribriform	focal	NA	NA
23	DCIS	DCIS024_A29	2	cribriform	focal	NA	NA
24	DCIS	DCIS024_A29	2	cribriform	focal	NA	NA
25	DCIS	DCIS024_A29	2	cribriform	focal	NA	NA
101	DCIS	DCIS065_A5	3	comedo, solid	comedo-like	NA	NA
102	DCIS	DCIS065_A5	3	solid	focal	NA	NA
103	DCIS	DCIS065_A5	3	solid	focal	NA	NA
104	Invasive	DCIS065_A5	NA	NA	NA	3	3
105	DCIS	DCIS065_A5	3	solid	focal	NA	NA
106	Invasive	DCIS065_A5	NA	NA	NA	3	3
108	Invasive	DCIS065_A5	NA	NA	NA	3	3
109	Invasive	DCIS065_A5	NA	NA	NA	3	3
110	Invasive	DCIS065_A5	NA	NA	NA	3	3

111	Invasive	DCIS065_A5	NA	NA	NA	3	3
112	Invasive	DCIS065_A5	NA	NA	NA	3	3
114	Invasive	DCIS065_A5	NA	NA	NA	3	3
115	Invasive	DCIS065_A5	NA	NA	NA	3	3
116	Invasive	DCIS065_A5	NA	NA	NA	3	3
117	Invasive	DCIS065_A5	NA	NA	NA	3	3
119	Invasive	DCIS065_A5	NA	NA	NA	3	3
120	Invasive	DCIS065_A5	NA	NA	NA	3	3
121	DCIS	DCIS065_A5	3	solid	focal	NA	NA
124	DCIS	DCIS065_A5	3	comedo, solid	comedo-like	3	3
125	Invasive	DCIS065_A5	3	NA	NA	NA	NA
127	DCIS	DCIS065_A5	3	solid	focal	NA	NA
128	Normal	DCIS065_A5	NA	NA	NA	NA	NA
34	Invasive	A7	3	comedo, solid	comedo-like	2	3
35	DCIS	A7	3	solid	no	NA	NA
36	DCIS	A7	3	comedo	comedo-like	NA	NA
37	DCIS	A7	2	solid	focal	NA	NA
38	DCIS	A7	3	comedo	comedo-like	NA	NA
39	DCIS	A7	2	solid	no	NA	NA
40	DCIS	A7	3	solid, comedo	comedo-like	NA	NA
41	DCIS	A7	2	solid	comedo-like	NA	NA
42	DCIS	A7	3	solid, comedo	comedo-like	NA	NA
43	DCIS	A7	2	solid, comedo	comedo-like	NA	NA
44	DCIS	A7	3	solid, comedo	comedo-like	NA	NA
45	DCIS	A7	3	solid	no	NA	NA

Supplemental Table S2. Clinical Characteristics of Eighteen-Sample Cohort. Within the race column, A denotes African American while W denotes White. R indicates right and L indicates left in the laterality column. – indicates absence of clinical information for that patient.

DCIS code	Age Dx	Race	Laterality	Surgery Type	Positive Lymph Nodes	Nodes # sampled	Distant Mets	Mets location	Lymphatic/ Vascular
DCIS-079	48.4	W	R	Lumpectomy	0	2	No	-	Absent
DCIS-081	48.7	W	R	-	1	13	No	Chest wall extension	Suspicious
DCIS-186	44.7	-	R	Mastectomy	4	17	No	-	Present
DCIS-069	62.3	W	R	-	2	22	-	-	-
DCIS-076	42.6	A	L	Mastectomy	0	4	No	-	Present, extensive
DCIS-091	73.5	W	L	Lumpectomy	1	2	No	-	Absent
DCIS-167	57.9	-	R	Partial mastectomy	0	3	-	-	Present
DCIS-187	74.4	-	L	Lumpectomy	0	2	-	-	Equivocal
DCIS-192	66.6	-	R	Lumpectomy	0	3	-	-	Absent
DCIS-260	87	-	L	Partial Mastectomy	0	3	-	-	Present
DCIS-264	54.8	W	L	Lumpectomy	0	2	-	-	Present
DCIS265	52.2	-	L	Partial mastectomy	0	2	-	-	Suspicious
DCIS273	46.1	-	R	Mastectomy	2	26	-	-	Suspicious

Supplemental Table S3. Pathological Characteristics of Eighteen-Sample Cohort. – indicates absence of clinical information for that patient. ND denotes not detected for that patient while NP denotes not present.

DCIS code	Nuclear Grade of DCIS	DCIS Histology	ER(D)	PR (D)	HER2 (D)	Invasive Histology	Invasive Size (CM)	ER Comp	ER Total	PR Comp	PR Total	HER2 IHC	Prolif Index (%)	FISH
DCIS-079	2	Ductal, cribriform, and solid	-	-	-	Ductal Nos	1.5	5,3	8	5,3	8	2+	13	Not amplified
DCIS-081	2	Solid, cribiform	-	-	-	Ductal with lobular features	2.0,1.0	5,3	8	5,3	8	2	ND	Not amplified
DCIS-186	2	Solid, lobular	(6-7)	8	2+	Ductal nos.	9.5	-	6-7	5,3	8	2+	-	Not amplified
DCIS-069	3	Comedo	-	-	-		3,1.1	-	0	-	(+)	(3+)	high	-
DCIS-076	3	Comedo, cribriform, and solid	-	-	-	Ductal	1.8	5,3	8	5, 2-3	7-8	2-3+	ND	Amplified
DCIS-091	3	Comedo	-	-	-	Ductal with lobular features	1.7	5,3	8	4,2	6	1+	ND	ND
DCIS-167	3	Solid	0	0	1+	Ductal with medullary features	2.7	-	0	-	0	1+	-	-
DCIS-187	3	Solid	0	0	0	Ductal	1.3	0	0	0	0	0	-	-
DCIS-192	3	Solid	0	0	2+	Ductal NOS	0.8	0	0	0	0	2+	-	Not amplified
DCIS-260	3	Solid and micropapillar y	6	2	0	Ductal w/ mucinous	4.2	-	6	-	2	0	-	-
DCIS-264	3	Comedo and solid	0	0	1+	Ductal NOS	1.6	0	0	0	0	1+	71	NP
DCIS265	3	Comedo and solid	2	2	0	Ductal	4.2	-	2	-	2	0	-	-

DCIS273	3	Comedo and solid	8	8	2+	Ductal with lobular	7	-	8	-	8	2+	-	-

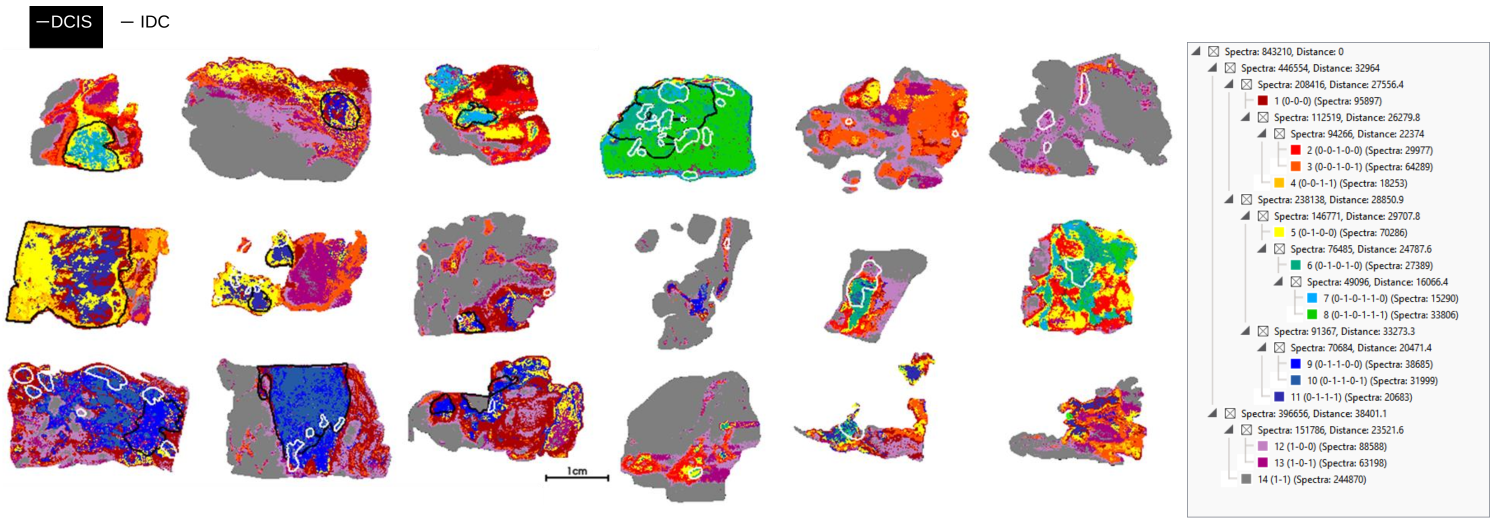

Supplemental Figure S2. Eighteen-sample Cohort Reports Uniquely Localized Proteomic Clusters. Segmentation analysis of 843,210 pixels from eighteen lumpectomies demonstrates uniquely localized proteomic groups to different pathological regions. Black annotation demarcates IDC lesions while white defines DCIS lesions.

Supplemental Table S4. Collagenase Proteomic Hits. Table summarizing collagenase digested identified proteins with spectrum counts and corresponding protein classifications derived from gene ontology terms.

Collagenase Digest Identified Proteins (208)	Quantitative value	GO Term 1	GO Term 2	GO Term 3	Protein Classification
Collagen alpha-2(I) chain	8755.64				Collagen
Collagen alpha-1(I) chain	4913.06				Collagen
Collagen alpha-1(III) chain	2487.72				Collagen
Collagen alpha-3(VI) chain	1791.29				Collagen
Collagen alpha-1(XIV) chain	891.68				Collagen
Collagen alpha-1(V) chain	895.75				Collagen
Collagen alpha-1(XII) chain	475.45				Collagen
Collagen alpha-1(VI) chain	514.06				Collagen
Collagen alpha-2(VI) chain	452.17		Organized by protein group		Collagen
Collagen alpha-2(V) chain	183.81				Collagen
Collagen alpha-1(XI) chain	141.54				Collagen
Collagen alpha-1(IV) chain	133.65				Collagen
Collagen alpha-2(IV) chain	98.5				Collagen
Collagen alpha-1(XVIII) chain	23.7				Collagen
Collagen alpha-1(VIII) chain	12.22				Collagen
Collagen alpha-6(VI) chain	3.49				Collagen
Collagen alpha-1(X) chain	2.7				Collagen
Elastin	2884		Organized by protein group		Elastin
Fibronectin	617.52	GO:0016324 Apical plasma membrane	GO:0005604 Basement membrane (part of Collagen-containing extracellular matrix)	GO:0072562 Blood microparticle	Extracellular matrix
Periostin	677.18	GO:0062023 Collagen-containing extracellular matrix	GO:0031012 Extracellular matrix	GO:0005615 Extracellular space	Extracellular matrix
Decorin	462.55<br>323.24	GO:0062023 Collagen-containing extracellular matrix	GO:0005576 Extracellular region	GO:0005615 Extracellular space	Extracellular matrix
Lumican	205.67	GO:0062023 Collagen-containing extracellular matrix	GO:0070062 Extracellular exosome	GO:0031012 Extracellular matrix	Extracellular matrix
Transforming growth factor-beta-ind	186.77	GO:0005604 Basement membrane	GO:0062023 Collagen-containing extracellular matrix	GO:0070062 Extracellular exosome	Extracellular matrix
Biglycan	218.46	GO:0009986 Cell surface	GO:0062023 Collagen-containing extracellular matrix	GO:0070062 Extracellular exosome	Extracellular matrix
Mimecan	155.21<br>144.56	GO:0062023 Collagen-containing extracellular matrix	GO:0070062 Extracellular exosome	GO:0031012 Extracellular matrix	Extracellular matrix
Alpha-1-antitrypsin	132.14	GO:0062023 Collagen-containing extracellular matrix	GO:0030134 COPII-coated ER to Golgi transport vesicle	GO:0005783 Endoplasmic reticulum	Extracellular matrix
Basement membrane-specific hepar	109.45	GO:0005604 Basement membrane (part of Collagen-containing extracellular matrix)	GO:0062023 Collagen-containing extracellular matrix	GO:0070062 Extracellular exosome	Extracellular matrix
Prolargin	44.42	GO:0062023 Collagen-containing extracellular matrix	GO:0005615 Extracellular space	GO:0031012 Extracellular matrix	Extracellular matrix

Tenascin	33.25	GO:0005604 Basement membrane (part of Collagen-containing extracellular matrix)	GO:0062023 Collagen-containing extracellular matrix	GO:0005788 endoplasmic reticulum lumen	Extracellular matrix
Microfibril-associated glycoprotein 4	23.11	GO:0062023 Collagen-containing extracellular matrix	GO:0071953 Elastic fiber	GO:0005576 Extracellular region	Extracellular matrix
Cathepsin D		GO:0062023 Collagen-containing extracellular matrix	GO:0031904 endosome lumen	GO:0010008 endosome membrane	Extracellular matrix
Pigment epithelium-derived factor		GO:0043203 axon hillock	GO:0005604 Basement containing extracellular Collagen-containing matrix extracellular matrix)	GO:0062023 Collagenmembrane (part of Extracellular matrix	

Versican core protein	13.77	GO:0062023 Collagen-containing extracellular matrix	GO:0005788 endoplasmic reticulum lumen	GO:0031012 Extracellular matrix	Extracellular matrix
Statherin	385.44	GO:0005576 extracellular region	GO:0019814 immunoglobulin complex		Extracellular matrix
Immunoglobulin gamma-1 heavy ch	127.65	GO:0005576 extracellular region		GO:0005886 plasma membrane	Extracellular matrix
Emilin-1	105.27	GO:0005581 Collagen trimer	GO:0062023 Collagen-containing extracellular matrix	GO:1990971 Emilin Complex	Extracellular matrix
Asporin	55.34 88	GO:0042995 Cell projection GO:0005576 Extracellular region	GO:0062023 Collagen- extracellular matrix	GO:0031012 Extracellular containing Extracellular matrix matrix	
Basic salivary proline-rich protein 1	46.45 32.38	GO:0062023 Collagen-containing extracellular matrix			Extracellular matrix
Tenascin-X	18.21		GO:0062023 Collagen-containing extracellular matrix		Extracellular matrix
Nidogen-1	14.51	GO:0062023 Collagen-containing extracellular matrix			Extracellular matrix
Cartilage intermediate layer protein	36.96 4.05	GO:0071013 catalytic step 2 spliceosome			Extracellular matrix
Heterogeneous nuclear ribonucleop	9.98	GO:0005576 Extracellular region GO:0070062 Extracellular exosome	GO:0062023 Collagen-containing extracellular matrix	GO:0070062 Extracellular exosome	Extracellular matrix
Basic salivary proline-rich protein 4	9.23		exosome space	GO:0005615 Extracellular	Extracellular matrix
Acid ceramidase	5.99	GO:0062023 Collagen-containing extracellular matrix	GO:0005576 Extracellular region	GO:0070062 Extracellular exosome	Extracellular matrix
Fibulin-5	7.22				Extracellular matrix
Thrombospondin-2	14.08			GO:0005615 Extracellular space	Extracellular matrix
Nidogen-2	15.84	GO:0005576 Extracellular region GO:0005615 Extracellular	GO:0031012 Extracellular exosome	GO:0070062 Extracellular matrix	Extracellular matrix

Calmodulin-like protein 5		3.49	space		GO:0062023 Collagen-containing extracellular matrix	Extracellular matrix
Salivary acidic proline-rich phospho			GO:0005576 Extracellular region			Extracellular matrix
Proline-rich protein 4		3.33			GO:0062023 Collagen-containing extracellular matrix	Extracellular matrix
			GO:0062023 Collagen-containing extracellular matrix			
			GO:0062023 Collagen-containing extracellular matrix		GO:0005576 Extracellular region	
					GO:0070062 Extracellular exosome	
Zinc-alpha-2-glycoprotein				GO:1904813 ficolin-1-rich granule lumen		Extracellular matrix
Target of Nesh-SH3						Extracellular matrix
				GO:0009897 external side of plasma membrane	GO:0070062 Extracellular exosome	
				GO:0005576 Extracellular region	GO:0005615 Extracellular space	

Fatty acid synthase	r	311.75	GO:0005737 Cytoplasm	GO:0005829 Cytosol	GO:0070062 Extracellular exosome	Cytosol
Glyceraldehyde-3-phosphate dehyd			GO:190411 axon cytoplasm	GO:0005829 Cytosol	GO:0070062 Extracellular exosome	Cytosol
Alpha-enolase		206.44	GO:0005938 Cell cortex (part of cytoplasm)	GO:0009986 Cell surface	GO:0005737 Cytoplasm	Cytosol
Heat shock protein beta-1		169.92	GO:190411 axon cytoplasm	GO:0001533 Cornified envelope	GO:0005737 Cytoplasm	Cytosol
Phosphoglycerate kinase 1		115.97	GO:0005737 Cytoplasm	GO:0070062 Extracellular exosome	GO:0005615 Extracellular space	Cytosol
Myosin regulatory light chain 12A		52.91	GO:0005737 Cytoplasm	GO:0005829 Cytosol	GO:0070062 Extracellular exosome	Cytosol
		51.86				
Endoplasmic reticulum chaperone B		54.12	GO:0009986 Cell surface	GO:0005737 Cytoplasm	GO:0005829 Cytosol	Cytosol
		24.18				
Heat shock protein HSP 90-alpha		39.95	GO:0005829 Cytosol	GO:0070062 Extracellular exosome	GO:0048471 Perinuclear region of cytoplasm	Cytosol
Peroxiredoxin-1		8.09	GO:0005737 Cytoplasm	GO:0005829 Cytosol	GO:0070062 Extracellular exosome	Cytosol
Glucose-6-phosphate isomerase		29.06	GO:0060170 Ciliary membrane	GO:0005829 Cytosol	GO:0070062 Extracellular exosome	Cytosol
Ubiquitin-like modifier-activating enz		21.66	GO:0005829 Cytosol	GO:0005829 Cytosol	GO:0070062 Extracellular exosome	Cytosol
4-trimethylaminobutyraldehyde dehy			GO:0005737 Cytoplasm	GO:0005829 Cytosol	GO:0070062 Extracellular exosome	Cytosol

Elongation factor 2	A	16.69	GO:0016235 aggresome	GO:0005737 Cytoplasm	GO:0005829 Cytosol	Cytosol
Peroxiredoxin-2		16.23	GO:0005737 Cytoplasm	GO:0005829 Cytosol		Cytosol
Eukaryotic initiation factor 4A-I		15.13	GO:0005737 Cytoplasm	GO:0005829 Cytosol	GO:0016281 eukaryotic translation initiation factor 4F complex	Cytosol
Myosin light polypeptide 6		6.03	GO:0005903 Brush border	GO:0005829 Cytosol	GO:0070062 Extracellular exosome	Cytosol
Tubulin beta-2A chain		69.46	GO:0005737 Cytoplasm	GO:0070062 Extracellular exosome	GO:1903561 Extracellular vesicle	Cytosol

Macrophage migration inhibitory fac	236.09 101.64	GO:0009986 Cell surface	GO:0005737 Cytoplasm	GO:0005829 Cytosol	Cytosol
Peptidyl-prolyl cis-trans isomerase	12.81	GO:0005737 Cytoplasm	GO:0005829 Cytosol	GO:0070062 Extracellular exosome	Cytosol
Gamma-enolase	12.49	GO:0005829 Cytosol	GO:0070062 Extracellular exosome	GO:0005615 Extracellular space	Cytosol
Desmin	14.35 14.43	GO:0097512 Cardiac myofibril	GO:0005911 cell-cell junction	GO:0005829 Cytosol	Cytosol
Poly(rC)-binding protein 1	9.82	GO:0005737 Cytoplasm	GO:0036464 cytoplasmic ribonucleoprotein granule	GO:0005829 Cytosol	Cytosol
Glutathione S-transferase P	11.66	GO:0005737 Cytoplasm	GO:0005829 Cytosol	GO:0070062 Extracellular exosome	Cytosol
14-3-3 protein theta	16.73	GO:0005737 Cytoplasm	GO:0005829 Cytosol	GO:0070062 Extracellular exosome	Cytosol
Galectin-3	17.6 7.59		GO:0062023 Collagen-containing extracellular matrix	GO:0005737 Cytoplasm	Cytosol
Ferritin light chain	10.83 4.05	GO:0009986 Cell surface GO:0044754 autolysosome	GO:0035578 azurophil granule lumen	GO:0005737 Cytoplasm	Cytosol
Apolipoprotein D	7.53 3.16	GO:0005737 Cytoplasm	GO:0022626 cytosolic ribosome	GO:0030425 dendrite	Cytosol
14-3-3 protein beta/alpha	2.02	GO:0005737 Cytoplasm	GO:0005829 Cytosol	GO:0070062 Extracellular exosome	Cytosol
Myosin-11	2.65	GO:0005737 Cytoplasm	GO:0005829 Cytosol	GO:0070062 Extracellular exosome	Cytosol
Phosphoglycerate mutase 2	3.54	GO:0005829 Cytosol	GO:0070062 Extracellular exosome		Cytosol
Angiotensinogen		GO:0072562 Blood microparticle	GO:0062023 Collagen-containing extracellular matrix	GO:0005829 Cytosol	Cytosol
Transaldolase		GO:0005737 Cytoplasm	GO:0005829 Cytosol	GO:0070062 Extracellular exosome	Cytosol
D-dopachrome decarboxylase-like p		GO:0005737 Cytoplasm	GO:0070062 Extracellular exosome	GO:0005615 Extracellular space	Cytosol
Polyadenylate-binding protein 1		GO:0005737 Cytoplasm	GO:0005829 Cytosol	GO:0106002 mCRD-mediated mRNA stability complex	Cytosol
Cytosol aminopeptidase		GO:0005737 Cytoplasm	GO:0005829 Cytosol	GO:0070062 Extracellular exosome	Cytosol
Actin, cytoplasmic 1	769.63	GO:0015629 Actin GO:0005884 Actin filament cytoskeleton (in cytoskeletal GO term)		GO:0005912 Adherens junction	Cytoskeleton
Vimentin	603.65 545.17	GO:0030424 axon	GO:0031252 cell leading edge	GO:0005737 Cytoplasm	Cytoskeleton (next GO term)
Filamin-A	488.19	GO:0015629 Actin GO:0005884 Actin filament cytoskeleton (in cytoskeletal GO term)		GO:0032432 actin filament bundle	Cytoskeleton
Actin, alpha cardiac muscle 1	482.6	GO:0005884 Actin filament (in cytoskeletal GO term)	GO:0072562 Blood microparticle	GO:0005737 Cytoplasm	Cytoskeleton
Tubulin beta chain	339.29	GO:0005879 Axonemal microtubule (within cytoskeletal GO term)	GO:0035578 Azurophil granule lumen	GO:0005737 Cytoplasm	Cytoskeleton
Tubulin beta-4B chain	327.5	GO:0005879 Axonemal microtubule (within cytoskeletal GO term)	GO:0035578 Azurophil granule lumen	GO:0005737 Cytoplasm	Cytoskeleton
Tubulin alpha-1A chain		GO:0005879 Axonemal	GO:0000793 Condensed chromosome	GO:0005737 Cytoplasm	Cytoskeleton

	199.1	microtubule (within cytoskeletal GO term)			
	150.85				
	83.43				
Elongation factor 1-alpha 1		GO:0030864 Cortical actin cytoskeleton		GO:0098574 cytoplasmic side of lysosomal membrane	Cytoskeleton
Gelsolin		GO:0030478 Actin cap (in GO:0015629 Actin actin cytoskeleton term) cytoskeleton		GO:0072562 Blood microparticle	Cytoskeleton
Filamin-B		GO:0015629 Actin cytoskeleton	GO:0005903 Brush border	GO:0005938 Cell cortex (part of cytoplasm)	Cytoskeleton

Fructose-bisphosphate aldolase A	92.02	GO:0015629 Actin cytoskeleton	GO:0005829 Cytosol	GO:0070062 Extracellular exosome	Cytoskeleton
	74.77			GO:0042995 Cell projection	
Alpha-actinin-4		GO:0015629 Actin cytoskeleton	GO:0030054 Cell junction		Cytoskeleton
	76.87				
		GO:0005884 Actin filament	GO:0030863 cortical		
	20.9			GO:0005856 Cytoskeleton	Cytoskeleton
Tropomyosin alpha-4 chain					
	37.13	(in cytoskeletal GO term)	cytoskeleton		
Tropomyosin alpha-3 chain	51.35	GO:0015629 Actin	GO:0005884 Actin filament		
	50.4			GO:0005856 Cytoskeleton	Cytoskeleton
		cytoskeleton (in cytoskeletal GO term)			
	31.03	GO:0015629 Actin cytoskeleton		GO:0005826 actomyosin	Cytoskeleton
Myosin-9					
	29.74		GO:0042641 Actomyosin contractile ring		
	32.39	GO:0072562 Blood microparticle	GO:0005938 Cell cortex		Cytoskeleton (keyterm)
Profilin-1				GO:0005737 Cytoplasm	
	12.05				
			(part of cytoplasm)		
	13.82				
Dihydropyrimidinase-related protein		GO:0005856 Cytoskeleton	GO:0005829 Cytosol	GO:0070062 Extracellular exosome	Cytoskeleton
	5.35	GO:0015629 Actin cytoskeleton	GO:0005829 Cytosol	GO:0070062 Extracellular exosome	Cytoskeleton
Transgelin-2	11.87				
Ras GTPase-activating-like protein	5	GO:0005884 Actin filament (in cytoskeletal GO term)	GO:0016324 apical plasma membrane	GO:0030424 axon	Cytoskeleton
Clusterin		GO:0005813 centrosome (part of cytoskeleton)	GO:0097546 ciliary base	GO:0097542 ciliary tip	Cytoskeleton
	78.73				
Neuroblast differentiation-associate		GO:0015629 Actin cytoskeleton	GO:0044291 Cell to cell contact zone	GO:0043034 costamere	Cytoskeleton
	17.47				
Major vault protein		GO:0005737 Cytoplasm	GO:0005856 Cytoskeleton	GO:0005829 Cytosol	Cytoskeleton
	13.87				
Cofilin-1		GO:0015629 Actin cytoskeleton	GO:0005737 Cytoplasm	GO:0005829 Cytosol	Cytoskeleton
	5.83				
Plastin-2	12.6	GO:0015629 Actin cytoskeleton	GO:0005829 Cytosol	GO:0005886 Plasma membrane	Cytoskeleton
	3.54			GO:0005911 cell-cell junction	
Coronin-1A		GO:0005884 Actin filament (in cytoskeletal GO term)	GO:0030424 axon		Cytoskeleton
	2.71				
	3.37				
Beta-actin-like protein 2		GO:0005884 Actin filament (in cytoskeletal GO term)	GO:0030424 axon	GO:0005737 Cytoplasm	Cytoskeleton

Rho GDP-dissociation inhibitor 1		GO:0005856 Cytoskeleton	GO:0005829 Cytosol	GO:0070062 Extracellular exosome	Cytoskeleton
Calmodulin-1		GO:0034704 Calcium channel complex	GO:1902494 catalytic complex	GO:0005813 centrosome (part of cytoskeleton term)	Cytoskeleton
Rho GDP-dissociation inhibitor 2		GO:0005856 Cytoskeleton	GO:0005829 Cytosol	GO:0070062 Extracellular exosome	Cytoskeleton
Dihydropyrimidinase-related protein		GO:0005813 centrosome (part of cytoskeleton)	GO:0005829 Cytosol	GO:0030426 growth cone	Cytoskeleton
Chloride intracellular channel protei		GO:0015629 Actin cytoskeleton		GO:0097512 cardiac myofibril	Cytoskeleton
Alpha-crystallin B chain		GO:0032432 actin filament bundle	GO:0030424 axon		Cytoskeleton
Calponin-2		GO:0015629 Actin cytoskeleton	GO:0005911 cell-cell junction	GO:0005856 Cytoskeleton	Cytoskeleton
ATP synthase subunit beta, mitocho		GO:0005743 Mitochondrial inner membrane	GO:0005759 Mitochondrial matrix	GO:0005753 Mitochondrial proton-transporting ATP synthase complex	Mitochondria
60 kDa heat shock protein, mitochon	28.16<br>26.36	GO:0009986 Cell surface	GO:0005905 Clathrin-coated vesicle	GO:0030135 Coated vesicle	Mitochondria (annotated as mitochondrial)
10 kDa heat shock protein, mitochon	4.05<br>7.33	GO:0070062 Extracellular exosome	GO:0016020 Membrane	GO:0005759 Mitochondrial matrix	Mitochondria
ATP synthase subunit alpha, mitoch		GO:0005743 Mitochondrial inner membrane	GO:0005759 Mitochondrial matrix	GO:0005753 Mitochondrial proton-transporting ATP synthase complex	Mitochondria
Heterogeneous nuclear ribonucleop	7.59	GO:0005829 Cytosol	GO:0016020 membrane	GO:0005654 Nucleoplasm	Nucleus (keyword)
Histone H2B type 1-K	70.84	GO:0005829 Cytosol	GO:0005615 Extracellular space	GO:0005654 Nucleoplasm	Nucleus (keyword)
Hemoglobin subunit alpha	323.53	GO:0072562 Blood microparticle	GO:0005829 Cytosol	GO:0031838 haptoglobinhemoglobin complex	Circulating
Albumin	238.11	GO:0072562 Blood microparticle	GO:0005737 Cytoplasm	GO:0005783 Endoplasmic reticulum	Circulating
Apolipoprotein A-I	214.02	GO:0072562 Blood microparticle	GO:0005829 Cytosol	GO:007168 Endocytic vesicle lumen	Circulating
Hemoglobin subunit beta	140.73	GO:0072562 Blood microparticle	GO:0005829 Cytosol	GO:007168 Endocytic vesicle lumen	Circulating

Fibrinogen gamma chain	119.52<br>108.01	GO:0072562 Blood microparticle	GO:0009986 Cell surface	GO:0062023 Collagen-containing extracellular matrix	Circulating
Immunoglobulin heavy constant alph	71.57	GO:0072562 Blood microparticle	GO:0070062 Extracellular exosome	GO:0005576 Extracellular region	Circulating
Complement C4-A	65.94	GO:0030424 Axon	GO:0072562 Blood microparticle	GO:0005601 Classicalcomplement-pathway C3/C5 convertase complex	Circulating
Inter-alpha-trypsin inhibitor heavy ch	70.79	GO:0072562 Blood microparticle	GO:0070062 Extracellular exosome	GO:0005576 Extracellular region	Circulating

Complement C3	65.35	GO:0035578 Azurophil granule lumen	GO:0072562 Blood microparticle	GO:0009986 Cell surface	Circulating
Alpha-1-antichymotrypsin	37.17<br>24.98	GO:0035578 Azurophil granule lumen	GO:0072562 Blood microparticle	GO:0062023 Collagen-containing extracellular matrix	Circulating
Immunoglobulin kappa constant	19.24	GO:0072562 Blood microparticle	GO:0070062 Extracellular exosome	GO:0005576 Extracellular region	Circulating
Ceruloplasmin	3.37	GO:0072562 Blood microparticle	GO:0005788 Endoplasmic reticulum lumen	GO:0070062 Extracellular exosome	Circulating
Fibrinogen beta chain	7.85	GO:0072562 Blood microparticle	GO:0005938 Cell cortex (part of cytoplasm)	GO:0009986 Cell surface	Circulating
Serotransferrin	129.84	GO:0016324 apical plasma membrane	GO:0045178 basal part of cell	GO:0016323 Basolateral plasma membrane<br>GO:0031410 Cytoplasmic vesicle	Circulating (secreted GO keyword)
Alpha-2-macroglobulin	57.17<br>3.37	GO:0072562 Blood microparticle	GO:0062023 Collagen-containing extracellular matrix		Circulating
Fibrinogen alpha chain	6.74	GO:0072562 Blood microparticle	GO:0009986 Cell surface	GO:0062023 Collagen-containing extracellular matrix	Circulating
14-3-3 protein zeta/delta	11.03	GO:0072562 Blood microparticle	GO:0005737 Cytoplasm	GO:0005829 Cytosol	Circulating
Alpha-2-antiplasmin	5<br>2.02	GO:0072562 Blood microparticle	GO:0009986 Cell surface	GO:0062023 Collagen-containing extracellular matrix	Circulating
Inter-alpha-trypsin inhibitor heavy ch	5	GO:0072562 Blood microparticle	GO:0062023 Collagen-containing extracellular matrix	GO:0070062 Extracellular exosome	Circulating
Immunoglobulin heavy constant mu		GO:0072562 Blood microparticle	GO:0009986 Cell surface	GO:0070062 Extracellular exosome<br>GO:0034707 chloride channel complex	Circulating
Laminin subunit alpha-4		GO:0072562 Blood microparticle	GO:0005903 Brush border	GO:0071682 endocytic vesicle lumen	Circulating
Hemopexin		GO:0072562 Blood microparticle	GO:0062023 Collagen-containing extracellular matrix	GO:0070062 Extracellular exosome	Circulating
Serum amyloid P-component		GO:0072562 Blood microparticle	GO:0062023 Collagen-containing extracellular matrix		Circulating
Heat shock cognate 71 kDa protein			GO:0072562 Blood microparticle	GO:0061202 clathrinsculpted gammaaminobutyric acid transport vesicle membrane	Multiple compartments
Heat shock 70 kDa protein 1A	143.56	GO:0005776 autophagosome	GO:0072562 Blood microparticle	GO:0005814 Centriole	Multiple compartments
Pyruvate kinase PKM	121.81<br>121.51	GO:0016235 aggresome<br>GO:0005929 Cilium	GO:0062023 Collagen-containing extracellular matrix	GO:0005737 Cytoplasm	Multiple compartments
Heat shock protein HSP 90-beta	102.34	GO:0034751 aryl hydrocarbon receptor complex	GO:0044295 axonal growth cone	GO:0009986 Cell surface	Multiple compartments
Annexin A5	29.06	GO:0062023 Collagen-containing extracellular matrix	GO:0005737 Cytoplasm	GO:0005829 Cytosol	Multiple compartments
Adipocyte enhancer-binding protein	28.93	GO:0062023 Collagen-containing extracellular matrix	GO:0005737 Cytoplasm	GO:0070062 Extracellular exosome	Multiple compartments
Transitional endoplasmic reticulum	23.87	GO:1904949 ATPase complex	GO:0035578 Azurophil granule lumen	GO:0005737 Cytoplasm	Multiple compartments

Transketolase	20.68	GO:0005829 Cytosol	GO:0005789 endoplasmic reticulum membrane	GO:0070062 Extracellular exosome	Multiple compartments
Polyubiquitin-B	21.32	GO:0005829 Cytosol	GO:0030666 endocytic vesicle membrane	GO:0005789 endoplasmic reticulum membrane	Multiple compartments

ADP-ribosylation factor 3	13.36	GO:0070062 Extracellular exosome	GO:0000139 Golgi membrane	GO:0048471 perinuclear region of cytoplasm	Multiple compartments
Fumarate hydratase, mitochondrial		GO:0005694 Chromosome	GO:0005737 Cytoplasm	GO:0005829 Cytosol	Multiple compartments
UDP-glucose 6-dehydrogenase		GO:0005829 Cytosol	GO:0070062 Extracellular exosome	GO:0005654 Nucleoplasm	Multiple compartments
Proteasome activator complex subu		GO:0005829 Cytosol	GO:0016607 nuclear speck	GO:0005654 Nucleoplasm	Multiple compartments
Triosephosphate isomerase	3.37	GO:0005829 Cytosol	GO:0070062 Extracellular exosome	GO:0005615 Extracellular space	Multiple compartments
Thrombospondin-1	5.27<br>2.65	GO:0009986 Cell surface	GO:0062023 Collagen-containing extracellular matrix	GO:0005783 endoplasmic reticulum	Multiple compartments
Alpha-2-HS-glycoprotein	43.75<br>34.35	GO:0072562 Blood microparticle	GO:0062023 Collagen-containing extracellular matrix	GO:0005788 endoplasmic reticulum lumen	Multiple compartments
Cytoskeleton-associated protein 4	29.77	GO:0035577 azurophil granule membrane	GO:0005856 Cytoskeleton	GO:0005783 endoplasmic reticulum	Multiple compartments
Neurogenic locus notch homolog pr	18.71	GO:0009986 Cell surface	GO:0005829 Cytosol	GO:0005789 endoplasmic reticulum membrane	Multiple compartments
Inter-alpha-trypsin inhibitor heavy ch	12.03	GO:0072562 Blood microparticle	GO:0062023 Collagen-containing extracellular matrix	GO:0005788 endoplasmic reticulum lumen	Multiple compartments
Laminin subunit gamma-1	10.92<br>14.42	GO:0005604 Basement membrane	GO:0062023 Collagen-containing extracellular matrix	GO:0005788 endoplasmic reticulum lumen	Multiple compartments
Apolipoprotein A-IV	3.37<br>3.54	GO:0072562 Blood microparticle	GO:0042627 chylomicron	GO:0062023 Collagen-containing extracellular matrix	Multiple compartments
Malate dehydrogenase, mitochondri	10.24	GO:0005737 Cytoplasm	GO:0070062 Extracellular exosome	GO:0016020 Membrane	Multiple compartments
Cytosolic non-specific dipeptidase	2.7	GO:0005829 Cytosol	GO:0070062 Extracellular exosome	GO:0005654 Nucleoplasm	Multiple compartments
Kininogen-1	4.05	GO:0072562 Blood microparticle	GO:0062023 Collagen-containing extracellular matrix	GO:0005788 endoplasmic reticulum lumen	Multiple compartments
Stress-70 protein, mitochondrial	4.05<br>2.02	GO:0005737 Cytoplasm	GO:0070062 Extracellular exosome	GO:0005925 focal adhesion (part of cell junction)	Multiple compartments
Isocitrate dehydrogenase [NADP], m	5	GO:0005737 Cytoplasm	GO:0070062 Extracellular exosome	GO:0005759 Mitochondrial matrix	Multiple compartments
Annexin A7	4.28	GO:0062023 Collagen-containing extracellular matrix	GO:0005737 Cytoplasm	GO:0005789 endoplasmic reticulum membrane	Multiple compartments
Phosphatidylethanolamine-binding p		GO:0005829 Cytosol	GO:0070062 Extracellular exosome	GO:0005634 Nucleus	Multiple compartments
Filaggrin-2		GO:0001533 Cornified envelope	GO:0005737 Cytoplasm	GO:0005576 Extracellular region	Multiple compartments

Protein disulfide-isomerase A3	45.66	GO:0009986 Cell surface	GO:0005783 endoplasmic reticulum	GO:0005788 endoplasmic reticulum lumen	Keyword ER
Calreticulin	18.7	GO:0005788 endoplasmic reticulum lumen	GO:0005789 endoplasmic reticulum membrane	GO:0005789 Nuclear envelope	ER
Neutral alpha-glucosidase AB	6.19	GO:0005783 endoplasmic reticulum	GO:0070062 Extracellular exosome	GO:0017177 glucosidase II complex (part of ER term)	ER
Serpin H1	10.31	GO:0062023 Collagen-containing extracellular matrix	GO:0005783 endoplasmic reticulum	GO:0005788 endoplasmic reticulum lumen	ER
Endoplasmin	3.54	GO:0062023 Collagen-containing extracellular matrix	GO:0005829 Cytosol	GO:0071682 endocytic vesicle lumen	Keyword ER
Protein disulfide-isomerase	20.59	GO:0005783 endoplasmic reticulum	GO:0005788 endoplasmic reticulum lumen	GO:0005789 endoplasmic reticulum membrane	ER
Hypoxia up-regulated protein 1	6.91	GO:0071682 endocytic vesicle lumen	GO:0005783 endoplasmic reticulum	GO:0034663 endoplasmic reticulum chaperone complex	ER
Vinculin	63.78	GO:0005912 Adherens junction	GO:0005903 Brush border	GO:0042995 Cell projection	Membrane
Annexin A1	8.47	GO:0005912 Adherens junction	GO:0016324 apical plasma membrane	GO:0016323 Basolateral plasma membrane	Membrane
Chloride intracellular channel protein	6.07	GO:0034707 chloride channel complex (part of membrane term)	GO:0005737 Cytoplasm	GO:0070062 Extracellular exosome	Membrane
Alpha-actinin-1	62.05	GO:0005903 Brush border	GO:0030054 Cell junction	GO:0042995 Cell projection	Membrane
Talin-1	39.2	GO:0005912 Adherens junction	GO:0009986 Cell surface	GO:0005856 Cytoskeleton	Membrane
Protein NDRG1	4.72	GO:0005912 Adherens junction	GO:0005813 centrosome (part of cytoskeleton)	GO:0005737 Cytoplasm	Membrane
Spectrin alpha chain, non-erythrocyt	2.65	GO:0030054 Cell junction	GO:0042995 Cell projection	GO:0030864 Cortical actin cytoskeleton	Membrane
Aldo-keto reductase family 1 membe	3.54	GO:0016324 apical plasma membrane	GO:0005829 Cytosol	GO:0070062 Extracellular exosome	Membrane
Plectin	39.71	GO:0030424 axon	GO:0005903 Brush border	GO:0043034 costamere	Other compartments
Clathrin heavy chain 1	12.05	GO:0030118 clathrin coat	GO:0030132 clathrin coat of coated pit	GO:0030130 clathrin coat of trans-Golgi network vesicle	Other compartments
Polymeric immunoglobulin receptor					Other compartments
Tripeptidyl-peptidase 1					Other compartments
Lysosomal alpha-glucosidase	28.16	GO:0035578 Azurophil granule lumen	GO:0070062 Extracellular exosome	GO:0005615 Extracellular space	Other compartments
Suprabasin	2.65	GO:0005764 Lysosome			Other compartments
		GO:0120282	GO:0035577 azurophil	GO:0070062 Extracellular	

	6.98 autolysosome lumen	granule membrane	exosome	
	GO:0070062 Extracellular			
	7.04 exosome			

Supplemental Table S5. Collagenase Peptide Peak Identifications. Mass spectrometry imaging peaks were matched to LC-MS/MS peaks within 5 ppm. HYP denotes hydroxylated proline sequences while Deamid delineates deamidated residues. Numerical values following proline residues indicate probability of hydroxylation at the site. If two sequences were within 5 ppm of imaging peak, both peptide sequences and their associated proteins are provided with "or" between. If a semicolon separates the gene names, then peptide sequence mapped to multiple proteins.

**Gene Name of Protein**	**MSI m/z**	**Leading razor protein**	**Hydroxyproline Probabilities**	**MS/MS m/z**	**Charge**	**Score**	**Mass Error [ppm]**	**Delta Score**	**No. scans**	**MS/MS count**	**MS/MS scan number**	**Intensity**	**Best MS/MS**
COL3A1	**758.452**	P02461	IAGITGAR	380.249	2	143.02	0.94483	73.63	63	4	17389	5218000	11188
COL2A1; COL1A1	**797.426**	P02452	GAAGRVGP(0.5)P(0.5)	399.217	2	100.07	0.11456	41.085	114	1	4337	4906100	1733
COL2A1; COL1A1; COL3A1; COL5A2	**976.452**	P02452	GLQGM(Ox)P(1)GER	488.737	2	85.212	1.4826	14.944	19	1	3414	635090	3667
COL3A1	**999.595**	P02461	IAGITGARGLA	500.304	2	126.41	1.1621	39.579	62	2	33195	211000000	11208
COL1A1	**1060.527**	P02452	GAKGLTGSP(1)GSP(1)	530.770	2	98.055	-1.0774	130.66	6	1	4262	3580900	1832
COL1A1	**1068.507**	P02452	GPAGERGSP(1)GPA	534.760	2	76.679	1.0432	7.0807	49	1	3506	1572100	4099
COL3A1; COL1A1	**1082.632**	P02461, P02452	GPLGIAGITGAR or PLGIAGITGARG or IAGQRGVVGLP(1)	1082.632, 541.823, 1082.632	1, 2,1	200.56, 193.1, 113.5	-3.4706, 2.8085, -3.2046	138.02, 128.06	43, 48, 25	4, 2, 2	44949, 40621, 29808	67726000, 47234000, 15039000	4879, 15099, 11227
COL1A1	**1084.502**	P02452	GPSGASGERGP(0.06)P(0.94)	542.758	2	94.692	-0.62069	45.898	25	1	3970	969910	9486
COL3A1	**1089.485**	P02461	GP(0.01)P(0.999)GTAGFP(1)GSP(1)	545.248	2	187.55	-0.62013	187.55	56	3	27931	19992000	6749
COL2A1	**1098.506**	P02458-1	GPSGEP(0.033)GKQ(Deamid)GAP(0.967)	549.757	2	94.85	1.0115	7.5918	20	1	4253	790030	9494
COL1A2; COL1A1	**1098.590**	P08123	GPVGRTGEVGAV or GARGLP(HYP)GTAGLP(HYP)	549.800	2	135.43	-3.4226	79.171	93	6	22287	528420000	9817
COL1A1	**1102.564**	P02452	GIAGAP(1)GFP(1)GAR	551.787	2	127.02	0.052665	127.02	84	5	27692	108440000	2673
COL1A1	**1115.544**	P02452	GAKGDRGETGPA	558.276	2	112.89		64.858		1	22209		1830
COL1A2	**1125.528**	P08123	GP(0.076)P(0.924)GVSGGGYDF	1125.497	1	135.83	-0.76261	66.071	25	2	48133	5977800	6863
COL1A2 or COL1A1	**1128.528**	P02452	GSP(1)GERGEVGPA or GP(0.001)P(0.0999)GLGGN(Deamid)FAPQ	564.757, 564.772	2, 2	140.98, 107.9	0.6905, 1.0685	94.558, 48.915	41, 44	2, 2	82830, 44966	16918000	9000, 12863000
COL1A1	**1142.507**	P02452	GP(0.003)P(0.997)GESGREGAP(1)	571.760	2	108.15	0.28993	108.15	59	2	3251	3049300	5250
COL1A1	**1154.507**	P02452	GAP(0.333)GDRGEP(0.333)GP(0.026)P(0.308)	577.759	2	98.044	-1.7544	98.044	32	1	13418000	1880	
TNC	**1166.532**	P24821-4	FTTDLDSPRD	583.770	2	84.615		45.362		1	27152		1683
COL2A1;COL1A1	**1175.547**	P02452	GLQGM(Ox)P(1)GERGAA	588.281	2	96.331	-0.30787	21.222	145	1	8336	1941100	3671
COL4A2	**1205.580**	P08572	GPN(Deamid)GIPSDTLHP	603.296	2	115.82	-0.11725	21.133	52	2	34924	20351000	5096

COL1A1	**1212.622**	P02452	TGAKGEP(0.967)GP(0.033)VGVQ	606.818	2	81.799	0.10823	33.003	86	1	13615	1266300	15552
COL1A1	**1226.612**	P08123	GPAGEVGKP(1)GERG	613.813	2	89.567	1.3936	89.567	37	1	2995	1970800	4112
COL6A3 or COL1A1	**1229.591**	P12111-2, P02452	NNLFTSSAGYR or GFP(0.5)GERGVQGP(0.045)P(0.455)	615.301, 615.299	2, 2	115.86,11 3.1	-0.76859	53.899	24	1	34089	8285400	14815
COL3A1	**1248.568**	P02461	GP(0.044)RGQ(Deamid)P(0.456)GVM(Ox)GFP(0.5)	624.790	2	93.429	-0.27186	93.429	42	1	25320	38977000	9484
FGA	**1253.601**	P02671-2	LGEFVSETESR	627.805	2	140.16	-2.237	76.124	28	2	30062	8499200	13102
COL1A2	**1257.582**	P08123	GPAGATGDRGEAGAA	629.299	2	77.062	0.72815	33.755	143	1	7890	696420	4057
COL12A1	**1273.675**	Q99715	VGPQTTTLSVRD	637.343	2			45.093		1	27412		15916
COL1A2	**1280.685**	P08123	GVAGAVGEP(0.999)GP(0.001)LGIA	640.850	2	101.43	1.0684	68.59	105	1	59111	836620000	10406
COL1A1	**1283.586**	P02452	GPAGEKGSP(1)GADGPA	642.301	2	205.13	1.5672	118.76	195	3	4916	2692700	4066
COL2A1;COL1A1	**1286.660**	P02452	GQRGERGFPGLP(HYP)	643.838	2	121.6	-0.40952	66.054	91	3	28860	15656000	9960
COL6A6 or FN1	**1291.664**	A6NMZ7, P02751-3	VVQDFDVSLNR or LRNLQPASEYT	646.839, 646.341	2, 2	134.25, 95.815	-1.3191, 0.92361	89.528	19, 37	1, 1	40902, 29289	8665700, 10395000	16776, 13917
COL1A1	**1299.581**	P02452	GPAGAP(1)GDKGESGPS or GP(1)AGEKGSP(1)GADGPA	650.296	2	175.74	-2.827, 2.1723	67.273, 124.67	31, 25	1, 1	4128, 4299	13778000, 2549700	4023, 4077
COL1A2	**1326.633**	P08123	GPIGSAGP(0.5)P(0.5)GFP(1)GAP(1)	663.824	2	114.89	0.10315	114.89	51	1	46633	20263000	4722
COL4A4	**1349.644**	P53420	GFSGIDGARGP(1)KGN(Deamid)	675.327	2	89.029	-3.4386	24.608	25	1	31232	14827000	2306
COL1A2	**1351.697**	P08123	GPSGLPGERGAAGIP(1)	676.357	2	133.89	-0.54158	48.051	60	5	32223	457240000	9552
COL18A1	**1365.647**	P39060-2	GP(0.2)P(0.8)GQFPFDFLQ	683.329	2	96.668	-2.6028	59.477	91	3	94178	11380000	6680
COL1A2	**1379.717**	P08123	GLP(1)GVAGAVGEP(1)GPLG or GPAGKEGPVGLP(1)GID	690.365, 690.364	2, 2	85.67, 103.08	0.021978, -1.7105	85.67, 58.766	62, 30	1,2	59357, 39587	64107000, 10667000	3555, 4161
COL3A1	**1386.687**	P02461	GAAGERGAP(0.982)GFRGP(0.018)A	462.901	3	78.814	-1.9285	52.201	46	2	16853	2532000	1729
COL1A1	**1399.645**	P02452	GSP(1)GEAGRP(1)GEAGLP(1)	700.328	2	124.21	-1.7878	124.21	130	3	16627	19383000	10107
APP, TNXB or COL1A1	**1406.705**	P05067-10, P22105, P02452	ALEVYP(1)ELQ(Deamid)ITN, LSWTIPQGRFDS or FLPQPPQEKAHD	703.860, 703.861	2, 2, 3	96.711, 78.655, 65.179	0.43869, 0.032276, 0.48459	24.163, 43.95, 43.95	29, 22, 66	1, 1, 1	41098, 62833, 24937	89953000, 46385000, 5582500	80, 14181, 1065
COL1A1	**1421.786**	P02452	GPQGIAGQRGVVGLP(1)	711.399	2	153.1	-1.3138	117.39	40	3	38581	366870000	9329
COL1A1	**1458.701**	P02452	GLQGM(Ox)P(1)GERGAAGLP(1)	728.852	2	101.57	-2.4949	101.57	37	5	24369	626190000	3704
COL1A2	**1472.643**	P08123	GPNGDAGRP(1)GEP(1)GLM(HYP)	736.828	2	181.87	-3.5672	181.87	104	3	14683	3903500	5050
COL1A2	**1480.750**	P02461	GPRGERGEAGIP(1)GVP(1)	740.884	2	99.752	-0.32424	99.752	32	2	23589	9069700	9432

COL1A2	**1552.808**	P08123	GPVGAAGATGARGLVGEP(1)	776.917	2	89.959	2.7633	45.641	16	1	32517	4477300	9589
FN1	**1588.781**	P02751-3	VREEVVTVGN(Deamid)SVNEG	795.405	2	85.672	-1.4117	63.679	31	1	30599	9595400	16046
COL1A1	**1593.787**	P02452	GPP(HYP)GSAGAPGKDGLNGLP(HYP) or	797.400	2	145.06	-4.4359	145.06	41	2	30605	18726000	6690
COL3A1	**1595.777**	P02461	GPVGPAGKSGDRGESGPA	532.600	3	106.35	1.0175	70.161	87	1	11200	6515200	9782
COL1A2	**1599.736**	P08123	GAVGSP(1)GVNGAP(1)GEAGRD	800.379	2	111.12	-1.4695	111.12	60	1	14854	1762700	2041
COL1A1	**1609.797**	P02452	GP(0.014P(0.986)GFPGAVGAKGEAGPQ or GPVGPAGKSGDRGETGPA	805.410, 537.269	2, 3	151.74, 104.21	1.1779, -2.5252	85.468, 54.028	29, 125	2, 1	33330, 12371	17294000, 24640000	5663, 9787
FN1	**1681.817**	P02751-3	VTEATITGLEPGTEYT	841.417	2	112.36	-3.3233	78.491	33	2	60804	47572000	16687
COL6A1	**1692.819**	P12109	GREGPVGVP(1)GDP(1)GEAGPI	847.418	2	142.29	0.50733	142.29	56	3	35581	95414000	9990
FN1	**1767.924**	Q15063-5	VSWERSTTPDITGYR	589.984	3	87.669	2.5098	41.805	18	1	28101	11089000	13127

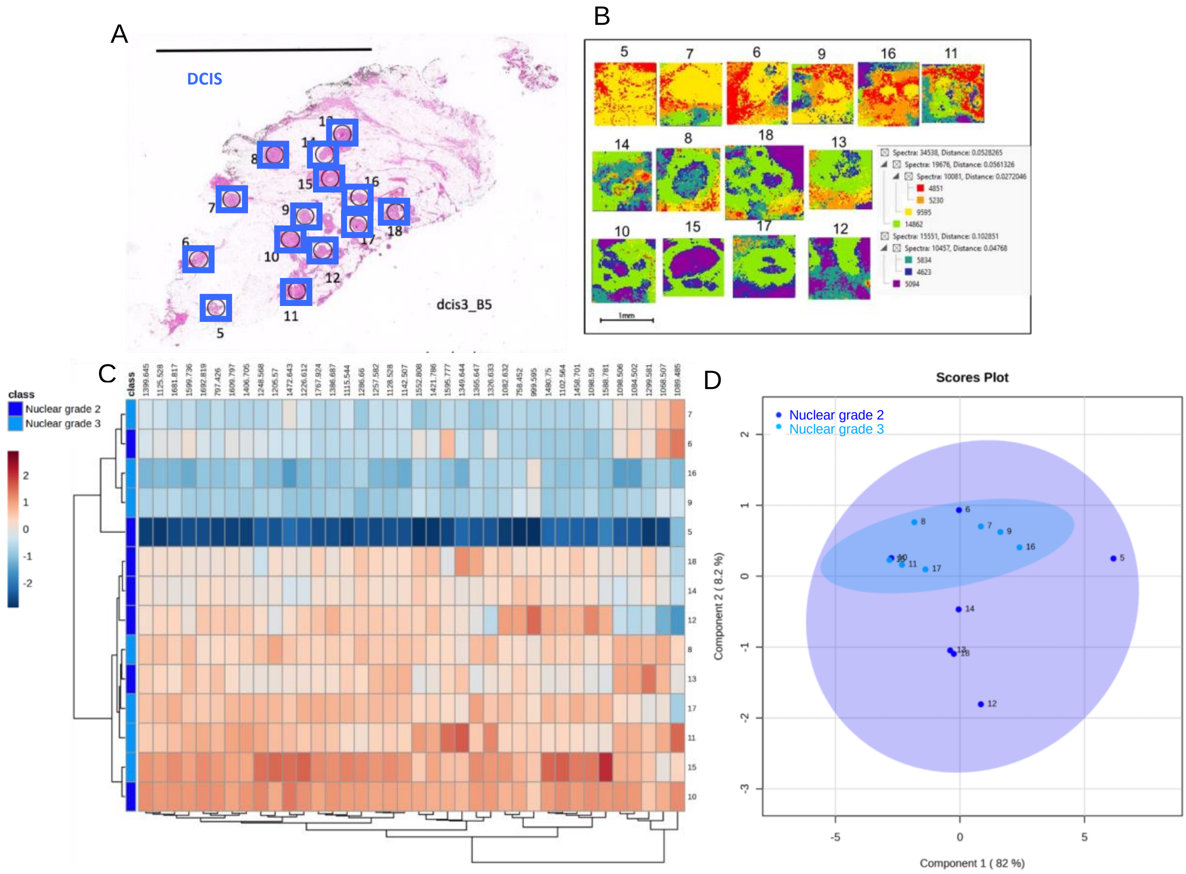

Supplemental Figure S3. Collagen Proteomic Analysis of DCIS003 (A) Pathologist-defined lesions circled in black. Blue delineates DCIS lesions while teal indicates the benign lesions. DCIS lesions were classified as the solid architectural pattern. **(B)** Segmentation analysis of 34,538 pixels reveal 7 spatially localized proteomic clusters within pathologist-defined regions of interest (ROIs). **(C)** Euclidean heatmap of LC-MS/MS identified peptides clustered by Ward method. **(D)** sPSL-DA demonstrates overlapping clusters of differing nuclear grade lesions.

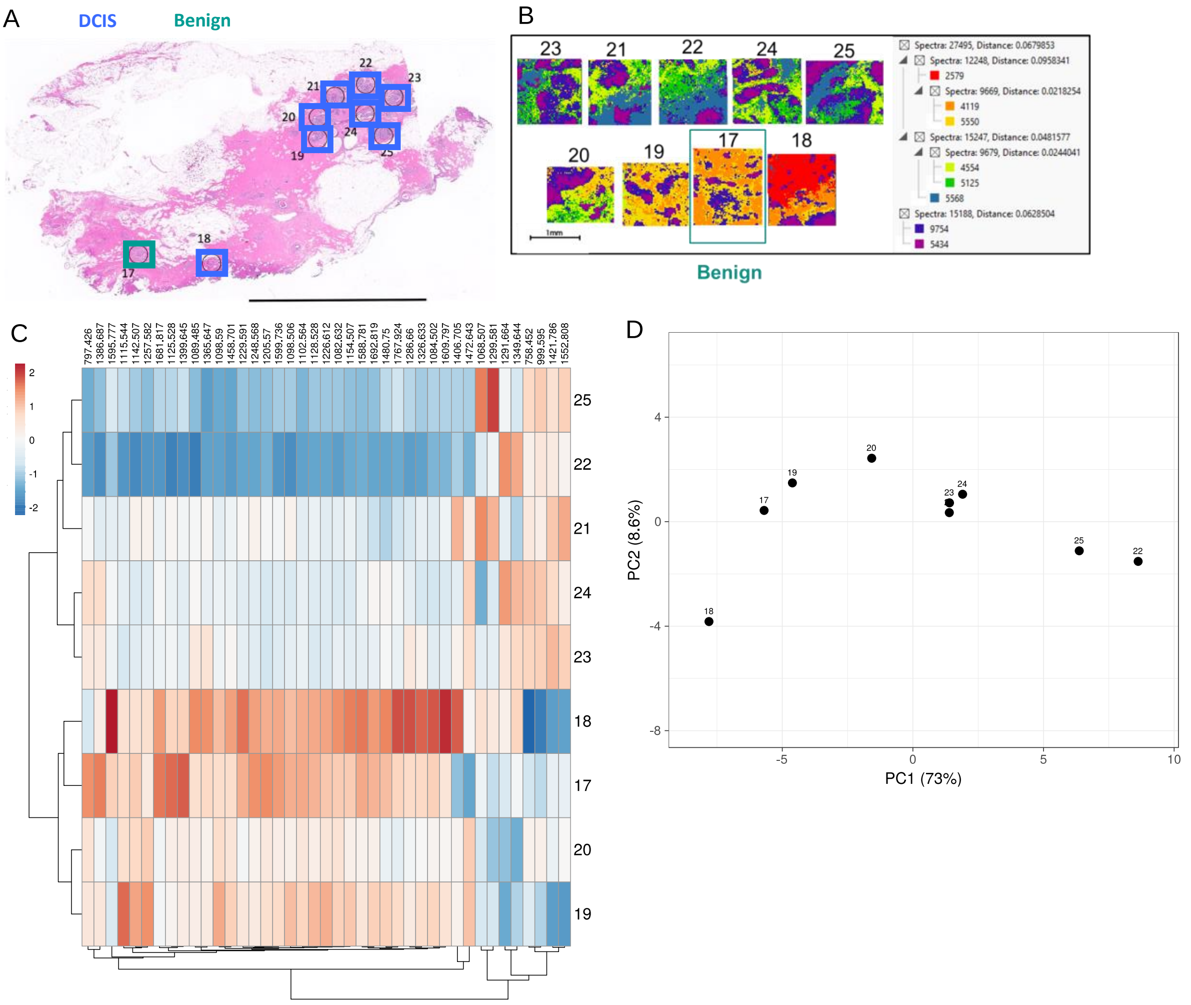

Supplemental Figure S4. Collagen Proteomic Analysis of DCIS024 (A) Pathologist-defined lesions circled in black. Blue delineates DCIS lesions while teal indicates the benign lesions. **(B)** Segmentation analysis of 27,495 pixels reveal 8 spatially localized proteomic clusters within pathologist-defined regions of interest (ROIs). **(C)** Heatmap of LC-MS/MS identified peptides generated in ClustVis. **(D)** PCA plot reports similarity in LC-MS/MS-identified peptide intensity profiles between ROIs generated in ClustVis.

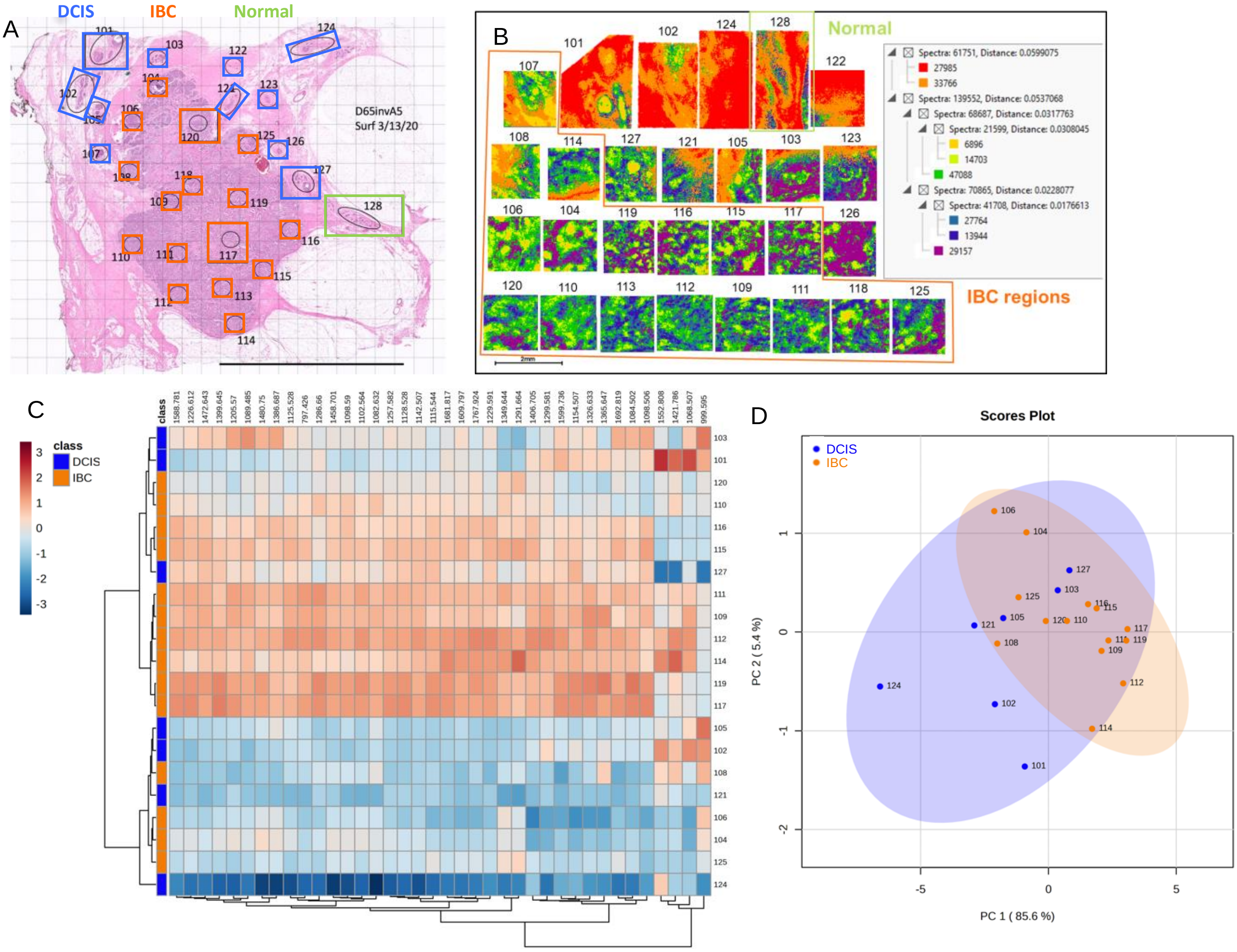

Supplemental Figure S5. Collagen Proteomic Analysis of DCIS065 (A) Pathologist-defined lesions circled in black. Blue delineates DCIS lesions while orange denotes IBC lesions. DCIS and IBC lesions were defined as nuclear grade 3. **(B)** Segmentation analysis of 61,751 pixels reveal 8 spatially localized proteomic clusters within pathologist-defined regions of interest (ROIs). ROIs closer together report more similar representation of proteomic groups. **(C)** Euclidean distance heatmap of LC-MS/MS identified peptides clustered by the Ward method demonstrates heterogeneity between DCIS and IBC proteomic profiles. **(D)** Principal component analysis plot of ROIs shows overlap between proteomic profiles DCIS and IBC pathologies.

Supplemental Table S6. Tryptic Proteomic Hits. Table summarizing tryptic digest identified proteins with spectrum counts and corresponding protein classifications derived from gene ontology terms.

Digest Identified Proteins (1644)	Total Tryptic Spectrum Counts	GO TERM 1	GO TERM 2	GO TERM 3	Protein classification
Collagen alpha-1(I) chain	800				collagen
Collagen alpha-1(III) chain	637				collagen
Collagen alpha-2(I) chain	345				collagen
Collagen alpha-1(VI) chain	40		Organized by protein group		collagen
Collagen alpha-2(V) chain	38				collagen
Collagen alpha-2(VI) chain	22				collagen
Collagen alpha-1(XIV) chain	29				collagen
Collagen alpha-6(VI) chain	2				collagen
Basement membrane-specific heparan sulfate proteoglycan core protein		GO:0005604 Basement membrane (part of Collagen-containing extracellular matrix)	GO:0062023 Collagen-containing extracellular matrix	GO:0070062 Extracellular exosome	Extracellular matrix
Lumican	3	GO:0062023 Collagen-containing extracellular matrix	GO:0070062 Extracellular exosome	GO:0031012 Extracellular matrix	Extracellular matrix
Prolargin	15	GO:0062023 Collagen-containing extracellular matrix	GO:0005615 Extracellular space	GO:0031012 Extracellular matrix	Extracellular matrix
Mimecan	12	GO:0062023 Collagen-containing extracellular matrix	GO:0070062 Extracellular exosome	GO:0031012 Extracellular matrix	
	13		GO:0005576 Extracellular region		Extracellular matrix
Decorin	5	GO:0062023 Collagen-containing extracellular matrix			Extracellular matrix
	4			GO:0005615 Extracellular space	
Biglycan	3	GO:0009986 Cell surface	GO:0062023 Collagen-containing extracellular matrixGO:0062023 Collagencontaining extracellular matrix	GO:0070062 Extracellular exosome	Extracellular matrix
Transforming growth factor-betainduced protein ig-h3	9	GO:0005604 Basement membrane		GO:0070062 Extracellular exosome	Extracellular matrix
Alpha-1-antitrypsin	3	GO:0062023 Collagen-containing extracellular matrix	GO:0030134 COPII-coated ER to Golgi transport vesicle	GO:0005783 Endoplasmic reticulum	Extracellular matrix
Galectin-1	4	GO:0062023 Collagen-containing extracellular matrix	GO:0005737 Cytoplasm	GO:0005788 Endoplasmic reticulum lumen	Extracellular matrix
Cathepsin D	3	GO:0062023 Collagen-containing extracellular matrix	GO:0031904 endosome lumen	GO:0010008 endosome membrane	Extracellular matrix
Fibrillin-1	4	GO:0005604 Basement membrane	GO:0062023 Collagen-containing extracellular matrix	GO:0005788 endoplasmic reticulum lumen	Extracellular matrix
Zinc-alpha-2-glycoprotein		GO:0062023 Collagen-containing extracellular matrix	GO:0009897 external side of plasma membrane	GO:0070062 Extracellular exosome	Extracellular matrix
Fatty acid synthase	21	GO:0005737 Cytoplasm	GO:0005829 Cytosol	GO:0070062 Extracellular exosome	Cytosol
Glyceraldehyde-3-phosphate dehydrogenase	9	GO:190411 axon cytoplasm	GO:0005829 Cytosol	GO:0070062 Extracellular exosome	Cytosol

Cytoplasmic dynein 1 heavy chain 1	2 3	GO:190411 axon cytoplasm	GO:0035578 Azurophil granule lumen	GO:0005938 Cell cortex (part of cytoplasm)	Cytosol
Annexin A6	7	GO:0062023 Collagen-containing extracellular matrix	GO:0005737 Cytoplasm	GO:0070062 Extracellular exosome	Cytosol
Alpha-enolase	10	GO:0005938 Cell cortex (part of cytoplasm)	GO:0009986 Cell surface	GO:0005737 Cytoplasm	Cytosol
Peroxiredoxin-1	7	GO:0005737 Cytoplasm	GO:0005829 Cytosol	GO:0070062 Extracellular exosome	Cytosol
Peptidyl-prolyl cis-trans isomerase A	2 2	GO:0005737 Cytoplasm	GO:0005829 Cytosol	GO:0070062 Extracellular exosome	Cytosol
Rab GDP dissociation inhibitor beta	4	GO:0035578 Azurophil granule lumen	GO:0005737 Cytoplasm	GO:0005829 Cytosol	Cytosol
RuvB-like 2		GO:0005829 Cytosol	GO:0120293 dynein axonemal particle (part of cytoplasm)	GO:0070062 Extracellular exosome	Cytosol
Endoplasmic reticulum chaperone BiP		GO:0009986 Cell surface	GO:0005737 Cytoplasm	GO:0005829 Cytosol	Cytosol
Heat shock protein beta-1	8	GO:190411 axon cytoplasm	GO:0001533 Cornified envelope	GO:0005737 Cytoplasm	Cytosol
Myosin regulatory light chain 12A	5	GO:0005737 Cytoplasm	GO:0005829 Cytosol	GO:0070062 Extracellular exosome	Cytosol
Eukaryotic initiation factor 4A-I	2	GO:0005737 Cytoplasm	GO:0005829 Cytosol	GO:0016281 eukaryotic translation initiation factor 4F complex	Cytosol
60S ribosomal protein L6	3 4	GO:0005737 Cytoplasm	GO:0036464 cytoplasmic ribonucleoprotein granule	GO:0005829 Cytosol	Cytosol
Glycogen phosphorylase, brain form	4	GO:0035578 Azurophil granule lumen	GO:0005737 Cytoplasm	GO:0070062 Extracellular exosome	Cytosol
Prohibitin	3	GO:0009986 Cell surface	GO:0005737 Cytoplasm	GO:0005769 early endosome	Cytosol
40S ribosomal protein S9	2	GO:0005737 Cytoplasm	GO:0005829 Cytosol	GO:0022626 cytosolic ribosome	Cytosol
40S ribosomal protein S3	2	GO:0005737 Cytoplasm	GO:0005829 Cytosol	GO:0022626 cytosolic ribosome	Cytosol
60S ribosomal protein L18	2	GO:0005737 Cytoplasm	GO:0005829 Cytosol	GO:0022625 cytosolic large ribosomal subunit	Cytosol
60S acidic ribosomal protein P0	4	GO:0005737 Cytoplasm	GO:0036464 cytoplasmic ribonucleoprotein granule	GO:0005829 Cytosol	Cytosol
40S ribosomal protein S15a	2	GO:0005737 Cytoplasm	GO:0005829 Cytosol	GO:0022626 cytosolic ribosome	Cytosol
60S ribosomal protein L3	3	GO:0022625 cytosolic large ribosomal subunit	GO:0016020 Membrane	GO:0005840 ribosome	Cytosol
Arylamine N-acetyltransferase 1		GO:0005829 Cytosol			Cytosol
Vimentin	23	GO:0030424 axon	GO:0031252 cell leading edge	GO:0005737 Cytoplasm	Cytoskeleton (next GO term)
Actin, cytoplasmic 1	21	GO:0015629 Actin cytoskeleton	GO:0005884 Actin filament (in cytoskeletal GO term)	GO:0005912 Adherens junction	Cytoskeleton
Neuroblast differentiation-associated protein AHNAK	16	GO:0015629 Actin cytoskeleton	GO:0044291 Cell to cell contact zone	GO:0043034 costamere	Cytoskeleton
Tubulin beta chain	33 21	GO:0005879 Axonemal microtubule (within cytoskeletal GO term)	GO:0035578 Azurophil granule lumen	GO:0005737 Cytoplasm	Cytoskeleton
Prelamin-A/C	6	GO:0005829 Cytosol	GO:0005882 Intermediate filament (in cytoskeletal GO term)	GO:0005638 Lamin filament	Cytoskeleton

Alpha-actinin-4	5	GO:0015629 Actin cytoskeleton	GO:0030054 Cell junction	GO:0042995 Cell projection	Cytoskeleton
Ras GTPase-activating-like protein IQGAP1	11 4	GO:0005884 Actin filament (in cytoskeletal GO term)	GO:0016324 apical plasma membrane	GO:0030424 axon	Cytoskeleton
Profilin-1	3	GO:0072562 Blood microparticle	GO:0005938 Cell cortex (part of cytoplasm)	GO:0005737 Cytoplasm	Cytoskeleton (key term)
Elongation factor 1-alpha 1	4 2	GO:0030864 Cortical actin cytoskeleton		GO:0098574 cytoplasmic side of lysosomal membrane	Cytoskeleton
Dihydropyrimidinase-related protein 2	19	GO:0005856 Cytoskeleton	GO:0005829 Cytosol	GO:0070062 Extracellular exosome	Cytoskeleton
Plastin-2	21	GO:0015629 Actin cytoskeleton	GO:0005829 Cytosol	GO:0005886 Plasma membrane	Cytoskeleton
T-complex protein 1 subunit gamma	30	ChaperoneGO:0044297 Cell body complex	GO:0005832 containing T	GO:0005856 Cytoskeleton	Cytoskeleton
Actin, alpha cardiac muscle 1	14	GO:0005884 Actin filament (in cytoskeletal GO term)	GO:0072562 Blood microparticle	GO:0005737 Cytoplasm	Cytoskeleton
Myosin-9	4 2	GO:0015629 Actin cytoskeleton	GO:0042641 Actomyosin	GO:0005826 actomyosin contractile ring	Cytoskeleton
Tubulin beta-4B chain	5	GO:0005879 Axonemal microtubule (within cytoskeletal GO term)	GO:0035578 Azurophil granule lumen	GO:0005737 Cytoplasm	Cytoskeleton
Tubulin alpha-1B chain		GO:0005737 Cytoplasm	GO:0005881 cytoplasmic microtubule	GO:0015630 microtubule cytoskeleton	Cytoskeleton
Unconventional myosin-Ic		GO:0015629 Actin cytoskeleton	GO:0005912 Adherens junction	GO:0005903 Brush border	Cytoskeleton
Transgelin		GO:0015629 Actin cytoskeleton	GO:0005829 Cytosol	GO:0070062 Extracellular exosome	Cytoskeleton
Peptidyl-prolyl cis-trans isomerase B		GO:0036064 ciliary basal body (part of cytoskeleton term)	GO:0005737 Cytoplasm	GO:0005829 Cytosol	Cytoskeleton

L-xylulose reductase	2	GO:0005903 Brush border	GO:0005881 cytoplasmic microtubule	GO:0005829 Cytosol	Cytoskeleton
ATP synthase subunit beta, mitochondrial	5 11 5 2	GO:0005743 Mitochondrial inner membrane	GO:0005759 Mitochondrial matrix	GO:0005753 Mitochondrial proton-transporting ATP synthase complex	Mitochondria
ATP synthase subunit alpha, mitochondrial	3 2 2 2	GO:0005743 Mitochondrial inner membrane	GO:0005759 Mitochondrial matrix	GO:0005753 Mitochondrial proton-transporting ATP synthase complex	Mitochondria
60 kDa heat shock protein, mitochondrial	4	GO:0009986 Cell surface	GO:0005905 Clathrin-coated vesicle	GO:0030135 Coated vesicle	Mitochondria (annotated as mitochondrial)

Citrate synthase, mitochondrial		GO:0070062 Extracellular exosome	GO:0005759 Mitochondrial matrix	GO:0005739 mitochondrion	Mitochondria
10 kDa heat shock protein, mitochondrial		GO:0070062 Extracellular exosome	GO:0016020 Membrane	GO:0005759 Mitochondrial matrix	Mitochondria
ATP synthase subunit O, mitochondrial		GO:0005743 Mitochondrial inner membrane	GO:0005753 Mitochondrial proton-transporting ATP synthase complex	GO:0000274 mitochondrial proton-transporting ATP synthase, stator stalk	Mitochondria
ADP/ATP translocase 2		GO:0016020 Membrane	GO:0005743 Mitochondrial inner membrane	GO:0042645 mitochondrial nucleoid	Mitochondria
EF-hand domain-containing protein D1		GO:0005743 Mitochondrial inner membrane	GO:0005739 mitochondrion		Mitochondria
Calcium-binding mitochondrial carrier protein SCaMC-1		GO:0016020 Membrane	GO:0005743 Mitochondrial inner membrane	GO:0005739 mitochondrion	Mitochondria
Histone H4	24	GO:0043505 CENP-A containing nucleosome			Nucleus
Heterogeneous nuclear ribonucleoproteins A2/B1		GO:0015030 Cajal body (part of nucleoplasm)	GO:0071013 catalytic step 2 spliceosome	GO:0000781 chromosome, telomeric	Nucleus region
Histone H3.1	10	GO:0000786 Nucleosome	GO:0005634 Nucleus		Nucleus
Histone H2B type 1-K	6	GO:0005829 Cytosol	GO:0005615 Extracellular space	GO:0005654 Nucleoplasm	Nucleus
X-ray repair cross-complementing protein 6	7<br>4	GO:0000785 Chromatin	GO:0000781 chromosome, telomeric region	GO:0070522 ERCC4ERCC1 complex	Nucleus
Histone H2A type 1	7	GO:0070062 Extracellular exosome	GO:0000786 Nucleosome	GO:0005634 Nucleus	Nucleus
Nucleolin	2	GO:0005938 Cell cortex (part of cytoplasm)	GO:0005694 Chromosome	GO:0001533 Cornified envelope	Nucleus
Histone H1.4	4	GO:0000792	GO:0000786 Nucleosome	GO:0005634 Nucleus	Nucleus heterochromatin
Histone H2A.Z	6	GO:0000791 euchromatin	GO:0070062 Extracellular exosome	GO:0000792 heterochromatin	Nucleus
Histone H1.5	3<br>2	GO:0000785 chromatin	GO:0005694 chromosome<br>GO:0016020 Membrane	GO:0000791 euchromatin	Nucleus
U5 small nuclear ribonucleoprotein 200 kDa helicase		GO:0071013 catalytic step 2 spliceosome		GO:0005654 Nucleoplasm	Nucleus
Hemoglobin subunit beta	39	GO:0072562 Blood microparticle	GO:0005829 Cytosol	GO:007168 Endocytic vesicle lumen	Circulating
Alpha-2-macroglobulin	6<br>30	GO:0072562 Blood microparticle	GO:0062023 Collagen-containing extracellular matrix	GO:0031410 Cytoplasmic vesicle<br>GO:0005576 Extracellular region	Circulating
Immunoglobulin heavy constant gamma 1	24	GO:0072562 Blood microparticle	GO:0070062 Extracellular exosome	GO:0031838 haptoglobinhemoglobin complex	Circulating
Hemoglobin subunit delta	37	GO:0072562 Blood microparticle	GO:0005829 Cytosol	GO:0005783 endoplasmic reticulum	Circulating
Serum Albumin Precursor	7	GO:0072562 Blood microparticle	GO:0005737 Cytoplasm	GO:0009986 Cell surface	Circulating
Complement C3	12	GO:0035578 Azurophil granule lumen	GO:0072562 Blood microparticle		Circulating

Hemoglobin subunit alpha	3	GO:0072562 Blood microparticle	GO:0005829 Cytosol	GO:0031838 haptoglobinhemoglobin complex	Circulating
Apolipoprotein A-I	9	GO:0072562 Blood microparticle	GO:0005829 Cytosol	GO:007168 Endocytic vesicle lumen	Circulating
Complement C4-B	8	GO:0030424 axon	GO:0072562 Blood microparticle	GO:0005601 Classicalcomplement-pathway C3/C5 convertase complex	Circulating
Serotransferrin		GO:0016324 apical plasma membrane	GO:0045178 basal part of cell	GO:0016323 Basolateral plasma membrane	Circulating (secreted GO keyword)
Immunoglobulin heavy constant alpha 2	2	GO:0005576 Extracellular space	GO:0005615 Extracellular receptor complex region	GO:0071744 IgE B cell	Circulating
Heat shock cognate 71 kDa protein	11	GO:0005776 autophagosome	GO:0072562 Blood microparticle	GO:0061202 clathrinsculpted gammaaminobutyric acid transport vesicle membrane	Multiple compartments
Pyruvate kinase PKM	5	GO:0005929 Cilium	GO:0062023 Collagen-containing extracellular matrix	GO:0005737 Cytoplasm	Multiple compartments
Heat shock protein HSP 90-beta	13	GO:0034751 aryl hydrocarbon receptor complex	GO:0044295 axonal growth cone	GO:0009986 Cell surface	Multiple compartments
Heat shock 70 kDa protein 1A	7	GO:0016235 aggresome	GO:0072562 Blood microparticle	GO:0005814 Centriole	Multiple compartments
Transitional endoplasmic reticulum ATPase	3	GO:1904949 ATPase complex	GO:0035578 Azurophil granule lumen	GO:0005737 Cytoplasm	Multiple compartments
Protein disulfide-isomerase	2	GO:0005856 Cytoskeleton	GO:0005829 Cytosol	GO:0005783 endoplasmic reticulum	Multiple compartments
ATP-dependent RNA helicase A	2	GO:0015629 Actin cytoskeleton	GO:0005813 centrosome	GO:0070937 CRDmediated mRNA stability complex	Multiple compartments
UDP-glucose 6-dehydrogenase	8	GO:0005829 Cytosol	GO:0070062 Extracellular exosome	GO:0005654 Nucleoplasm	Multiple compartments
Annexin A5	5	GO:0062023 Collagen-containing extracellular matrix	GO:0005737 Cytoplasm	GO:0005829 Cytosol	Multiple compartments
Polyubiquitin-B	2<br>2	GO:0005829 Cytosol	GO:0030666 endocytic vesicle membrane	GO:0005789 endoplasmic reticulum membrane	Multiple compartments
Probable ATP-dependent RNA helicase DDX17	2	GO:0005737 Cytoplasm	GO:0005829 Cytosol	GO:0016020 membrane	Multiple compartments
ADP-ribosylation factor 3	2	GO:0070062 Extracellular exosome	GO:0000139 Golgi membrane	GO:0048471 perinuclear region of cytoplasm	Multiple compartments
Prohibitin-2	3	GO:0030424 axon	GO:0071944 cell periphery	GO:0009986 Cell surface	Multiple compartments
Adenosylhomocysteinase	3	GO:0005829 Cytosol	GO:0005783 endoplasmic reticulum	GO:0070062 Extracellular exosome	Multiple compartments
Adipocyte enhancer-binding protein 1	2	GO:0062023 Collagen-containing extracellular matrix	GO:0005737 Cytoplasm	GO:0070062 Extracellular exosome	Multiple compartments
Ras-related protein Rab-11B	2	GO:0005829 Cytosol		GO:0005794 Golgi apparatus	Multiple compartments
Dolichyl-diphosphooligosaccharide-protein glycosyltransferase subunit STT3A	2			GO:0008250 oligosaccharyltransferase complex	Multiple compartments

High mobility group protein B1		GO:0005789 endoplasmic reticulum membrane; GO:0035868 alphav-beta3 integrin-HMGB1 complex	GO:0070062 Extracellular exosome; GO:0016020 Membrane; GO:0009986 Cell surface	GO:0000793 condensed chromosome	Multiple compartments
Endoplasmin	7	GO:0062023 Collagen-containing extracellular matrix	GO:0005829 Cytosol	GO:0071682 endocytic vesicle lumen	Keyword ER
Protein disulfide-isomerase A3	7	GO:0009986 Cell surface	GO:0005783 endoplasmic reticulum	GO:0005788 endoplasmic reticulum lumen	Keyword ER
Neutral alpha-glucosidase AB	3	GO:0005783 endoplasmic reticulum	GO:0070062 Extracellular exosome	GO:0017177 glucosidase II complex (part of ER term)	ER
Serpin H1	2	GO:0062023 Collagen-containing extracellular matrix	GO:0005783 endoplasmic reticulum	GO:0005788 endoplasmic reticulum lumen	ER
Dolichyl-diphosphooligosaccharide-protein glycosyltransferase subunit 1	3	GO:0005829 Cytosol	GO:0005783 endoplasmic reticulum	GO:0005789 endoplasmic reticulum membrane	ER
Moesin	4	GO:0005912 Adherens junction	GO:0045177 Apical part of cell	GO:0044297 Cell body	Membrane
Annexin A1	3	GO:0005912 Adherens junction	GO:0016324 apical plasma membrane	GO:0016323 Basolateral plasma membrane	Membrane
Desmoplakin	7	GO:0005912 Adherens junction	GO:0016323 Basolateral plasma membrane	GO:0001533 Cornified envelope	Membrane
Epiplakin	5	GO:0016327 apicolateral plasma membrane	GO:0016323 Basolateral bicellular plasma membrane	GO:0005923 tight junction	Membrane

Supplemental Table S7. Tryptic Peptide Peak Identifications. Mass spectrometry imaging peaks were matched to LC-MS/MS peaks within 5 ppm. HYP denotes hydroxylated proline sequences while Ox delineates oxidized residues. Numerical values following proline residues indicate probability of hydroxylation at the site. If two sequences were within 5 ppm of imaging peak, both peptide sequences and their associated proteins are provided. If a semicolon separates the gene names, then peptide sequence mapped to multiple proteins. Certain m/z values were identified as different peptide sequences.

Gene names	MSI m/z	Leading razor protein	Sequence	MS/MS m/z	Charge	MS/MS count	MS/MS scan number	Score	Mass error [ppm]	Delta score	MS/MS count	MS/MS scan number	Intensity	Best MS/MS
DYNC2H1	756.473	Q8NCM8	ALGGLLGR	378.74	2	1	28333	85.31	-0.1485	17.93	1	28333	20505000	2241
RPS4X	772.468	P62701	IGVITNR	386.739	2	2	24072	120.9	0.13904	56.622	2	24072	75911000	32830
PKP1	787.468	Q13835	EAVSLLR	394.237	2	1	38061	128.8	0.42695	51.007	1	38061	10655000	8976
COL6A3	787.468	P12111	EVITAVR	394.237	2	2	24882	148.3	0.31735	37.534	2	24882	5E+08	11734
UBA1	787.468	P22314	GGIVSQVK	394.237	2	1	19077	79.12	0.33236	0.5005	1	19077	6601500	21113
TKT	787.468	P29401	LILDSAR	394.236	2	1	33306	114.5	-0.29213	0	1	33306	10563000	38220
G6PD	787.468	P11413	LTVADIR	394.237	2	1	40693	105.2	-0.64758	11.607	1	40693	12461000	41410
USP9X	798.52	Q93008	AVLNLLR	399.763	2	1	54965	88.63	0.39309	24.064	1	54965	6928300	4290
CBR1	798.52	P16152	GIGLAIVR	399.763	2	1	51537	116.9	-0.33858	38.928	1	51537	9608900	21406
RPS3	798.52	P23396	LLGGLAVR	399.763	2	1	47606	111.8	0.49651	35.466	1	47606	75990000	38732
TGFBI	800.488	Q15582	ELANILK	400.747	2	1	44810	82.26	-0.89016	19.924	1	44810	31656000	10334
IGJ	800.488	P01591	IVLVDNK	400.747	2	1	28895	95.79	0.70419	18.419	1	28895	8710200	35087
LMNB1	800.488	P20700	LQIELGK	400.747	2	2	42586	106.4	0.090912	0	2	42586	13591000	40412
COL12A1	802.442	Q99715	GQEITVR	401.724	2	1	15044	88.03	-0.86379	18.645	1	15044	12031000	28314
TPM3;TPM4	802.442	P67936	NVTNNLK	401.724	2	1	6849	81.38	-0.64619	27.325	1	6849	30539000	45448
ALDH18A1	802.479	P54886	LGSAVVTR	401.742	2	1	18197	96.49	0.76423	46.372	1	18197	4918500	37869

PC	802.483	P11498	LFLQGPK	401.745	2	1	42901	72.02	#NUM!	50.474	1	42901		37388
KRT9	812.499	P35527	KGPAAIQK	406.752	2	1	4435	149.4	-1.2588	76.781	1	4435	5222100	35591
C1QB	812.499	P02746	TINVPLR	406.754	2	1	39658	99.92	0.73409	51.159	1	39658	9821200	54050
EPRS	814.515	P07814	LLSVNIR	407.761	2	1	46357	111.7	#NUM!	0	1	46357		39217
HIST1H2BL;HIST1H2BM;HIST1H2BN;HIST1H2BH;HIST2H2BF;HIST1H2BC;HIST1H2BD;H2BFS;HIST1H2BK;HIST1H2BA;HIST2H2BE;HIST1H2BB;HIST1H2BO;HIST1H2BJ	816.458	Q16778	EIQTAVR	408.876	2	4	11294	133.5	0.16534	45.483	4	11294	3.34E+08	10185
CKAP4	857.51	Q07065	LQNEILK	429.257	2	1	30446	129.4	-1.9292	30.253	1	30446	19231000	40462
POSTN	857.51	Q15063	QVIELAGK	429.258	2	1	32983	75.19	#NUM!	5.9557	1	32983		47727
TMED9	858.505	Q9BVK6	LSELQLR	429.756	2	1	40370	144.7	0.74975	37.811	1	40370	30138000	40809
EPHX1	871.5	P07099	QVEILNR	436.253	2	1	32709	85.89	-0.12354	6.7443	1	32709	7936200	47703
SHMT2	871.5	P34897	SAITPGGLR	436.852	2	2	30400	130	0.62638	65.282	2	30400	20779000	48677
APOD	871.5	P05090	VLNQELR	436.252	2	1	24080	148.3	-0.45628	53.613	1	24080	1.91E+08	59034
LAMB2	871.5	P55268	VVQDLAAR	436.252	2	1	20758	91.91	-2.071	37.381	1	20758	6011200	60757
NAMPT	871.541	P43490	LLPPYLR	436.274	2	1	54161	88.63	0.050148	34.09	1	54161	13901000	39055
C3	886.525	P01024	ISLPESLK	443.765	2	1	49226	104.8	1.0594	0.38094	1	49226	21232000	34525
SYNM	913.547	O15061	AALEALLGR	457.276	2	2	62050	113.4	0.088656	62.78	2	62050	12050000	195
DDX21	913.547	Q9NR30	AAVIGDVIR	457.278	2	1	45718	75.21	-0.66052	39.027	1	45718	5582300	434
VPS36	913.547	Q86VN1	AVGIVGIER	457.273	2	1	59714	89.05	#NUM!	11.989	1	59714		4252

PGM1	913.547	P36871	EAIQLIAR	457.276	2	1	45034	111.3	-1.77	40.366	1	45034	10710000	8813
FUBP3	913.547	Q96I24	LLGQIVDR	457.276	2	1	44145	77.92	0.56774	18.282	1	44145	10664000	38766
ADH1B	915.53	P00325	AAVLWEVK	458.269	2	1	55194	75.11	-0.40152	33.607	1	55194	54184000	442
ACTB;ACTG1;ACTA2;ACTG2;ACTC1;ACTA1;ACTBL2	923.568	P60709	IIAPPERK	462.287	2	1	9219	114.8	-0.36221	71.659	1	9219	25736000	32970
COL18A1	924.588	P39060	AAVPIVNLK	462.798	2	1	48730	94.41	-0.52014	54.304	1	48730	10160000	452
IGHA1	931.546	P01876	TPLTATLSK	466.276	2	1	35657	76.61	-0.03089	55.34	1	35657	1.08E+08	54785
HNRNPC	943.573	P07910	VPPPPPIAR	472.256	2	8	32372	138.1	0.49742	90.226	8	32372	41895000	59641
HIST1H2AC;HIST3H2A;HIST1H2AB;H2AFV;H2AFZ;HIST1H2AJ;HIST1H2AH;H2AFJ;HIST2H2AC;HIST2H2AA3;HIST1H2AD;HIST1H2AG;HIST1H2AA;H2AFX	944.532	Q99878	AGLQFPVGR	472.77	2	3	51654	114.6	0.035902	57.043	3	51654	3.24E+09	1428
RRBP1	944.567	Q9P2E9	LIEILSEK	472.786	2	1	53424	110.8	-0.53691	10.619	1	53424	19530000	38173
PCYOX1	945.562	Q9UHG3	TLLETLQK	473.284	2	1	47838	92.49	2.0713	37.942	1	47838	30125000	54382
TSTD1	957.548	Q8NFU3	GLQATQLAR	479.276	2	1	30878	75.85	#NUM!	30.136	1	30878		22949
SND1	957.548	Q7KZF4	QINLSNIR	479.277	2	1	43714	138.3	-0.14834	70.021	1	43714	29832000	46930
SNRPD2	957.573	P62316	GDSVIVVLR	479.289	2	1	52167	108.1	0.90832	52.646	1	52167	18901000	16268
FASN	957.573	P49327	GLVQALQTK	479.289	2	1	42596	107.7	-0.25936	58.247	1	42596	15193000	23423
COL6A1	957.573	P12109	IALVITDGR	479.289	2	2	49299	217.6	0.26498	132.28	2	49299	8.11E+08	31894
PRPF8	957.573	Q6P2Q9	IDLTLLNR	479.289	2	1	64256	117.2	#NUM!	48.747	1	64256		32169
LAMA5	957.573	O15230	ILLVTDGAR	479.289	2	1	43587	194.3	-0.50187	55.658	1	43587	1.11E+08	33700
COL12A1	957.573	Q99715	VLVVVTDGR	479.289	2	1	39129	130.2	0.32407	17.085	1	39129	16783000	59258
NAPRT	958.569	Q6XQN6	ALAQLSLSR	479.788	2	1	46709	119.3	-0.59431	69.932	1	46709	5381700	2032

SPTBN1	969.585	Q01082	VAVVNQIAR	485.294	2	1	33255	154.1	-0.31472	60.832	1	33255	27758000	56390
RBP1	970.569	P09455	ALDVNVALR	485.788	2	1	51097	126.7	-0.074	61.864	1	51097	19162000	2071
ALDH3A2	970.569	P51648	LQQLEALR	485.788	2	1	42624	83.36	#NUM!	17.56	1	42624		40563
PLS3	970.569	P13797	NEALAALLR	485.787	2	1	63468	116.3	0.31032	52.949	1	63468	27775000	43620
SEC24A	970.569	O95486	VVNLLQER	485.787	2	1	45296	81.3	2.391	51.111	1	45296	7549900	60741
FLNA;FLNB;FLNC	971.568	P21333	LLGWIQNK	486.287	2	1	57468	127.7	0.10954	127.66	1	57468	1.04E+08	38774
DHX30	981.61	Q7L2E3	IPQLLLER	491.308	2	1	60845	73.25	-1.3008	20.532	1	60845	1592600	34135
TUBA4A	985.568	P68366	DVNAAIAAIK	493.286	2	1	52094	81.3	-0.93363	46.001	1	52094	9127100	8246
DDX5	985.568	P17844	LLQLVEDR	493.287	2	1	49746	84.57	#NUM!	15.653	1	49746		39147
LDHB	1027.561	P07195	IHPVSTMVK	514.283	2	1	7150	73.67	-1.3462	44.327	1	7150	7219900	32917
SOD2	1028.61	P04179	GELLEAIKR	514.808	2	1	39534	122	-0.42106	56.737	1	39534	25125000	16742
ADRBK2;ADRBK1	1028.61	P35626	SLLEGLLQR	514.807	2	1	75764	93.56	-1.1269	50.495	1	75764	4007600	50355
PAFAH1B3	1041.606	Q15102	AIVQLVNER	521.306	2	1	44206	103.8	0.67403	16.51	1	44206	24384000	1930
KRT19;KRT18	1041.606	P08727	IVLQIDNAR	521.306	2	1	45428	206.5	0.032023	116.95	1	45428	1.84E+08	35065
RAB10;RAB15;RAB1A;RAB1C;RAB1B;RAB13;RAB8A;RAB8B	1071.641	P62820	LLLIGDSGVGK	536.324	2	1	58500	115.7	0.090683	0	1	58500	26615000	38931
CNN1	1091.574	P51911	GPAYGLSAEVK	546.29	2	1	41251	107.2	0.54119	84.111	1	41251	59783000	24053
SACM1L	1114.658	Q9NTJ5	TNVIQSLLAR	557.832	2	1	75198	90.91	-0.25012	45.226	1	75198	7583900	54699
MYH9	1155.664	P35579	RGDLPFVVPR	578.334	2	2	51330	192.3	-0.32793	154.7	2	51330	1.29E+08	48063
MAP7	1236.659	Q14244	ENVLFLTSGTR	618.828	2	1	60256	76.47	-3.6842	52.615	1	60256	9759400	11019
IQGAP1	1236.659	P46940	LQQTYAALNSK	619.328	2	2	33488	90.11	-2.1564	65.289	2	33488	8498800	40572
YWHAG	1236.659	P61981	YLAEVATGEKR	618.832	2	1	25341	101.7	0.92539	60.659	1	25341	2569400	61813
HIST1H4A	1325.754	P62805	DNIQGITKPAIR	442.589	3	3	37052	108.6	0.18051	92.986	3	37052	1.12E+09	7409
LGALS7	1481.844	P47929	SSLPEGIRPGTVLR	494.952	3	3	47505	104.6	-0.53706	91.613	3	47505	78648000	51365
ECHDC1	1506.744	Q9NTX5	ELYLEEALQNER	753.878	2	2	74124	177.1	2.2253	126.46	2	74124	12169000	10836
YBX1	1695.867	P67809	GAEAANVTGPGGVPVQGSK	848.434	2	1	36024	101.2	-0.8817	85.065	1	36024	5895800	14560
TUBAL3	1719.801	A6NHL2	SFGGGTGSGFTSLLMER	860.908	2	2	76076	136.3	-1.25	117.06	2	76076	14525000	49208

COL1A1	1764.878	P02452	GFP(1)GERGVQGP(0.006)P(0.947)GP(0.046)AGPR	588.965	3	2	33609	102.4	-0.50721	90.698	2	33609	60463000	19928

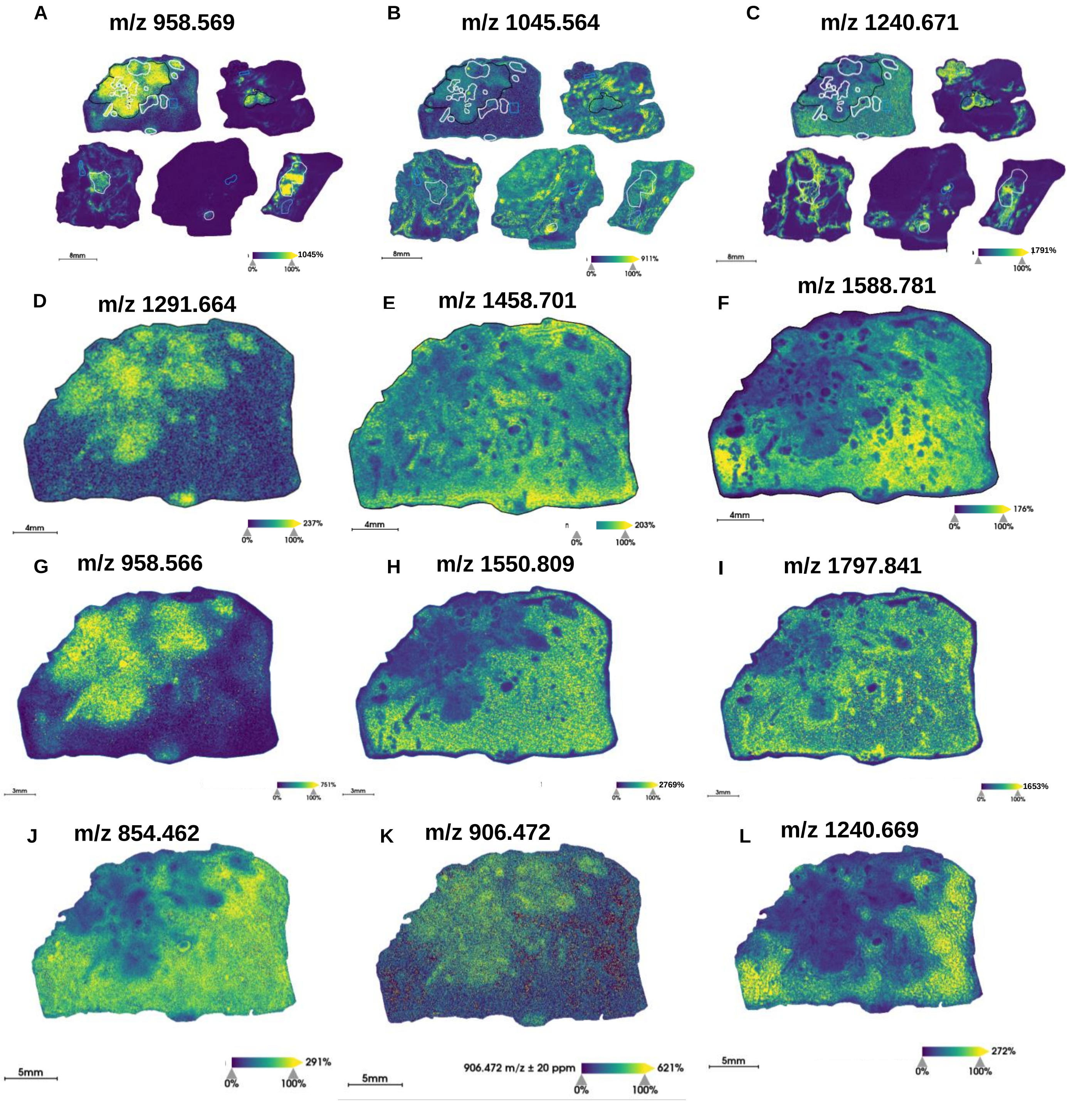

Supplemental Figure S6. Single Ion Images Demonstrate Spatial Distribution of Tryptic Peptides. (A-C) Single ion images of m/z 958.569, 1045.564, and 1240.671. Light blue annotations indicate normal adjacent tissue, white denotes DCIS lesions, and black delineates IDC region. **(C-D)** Single ion images of collagenase digested peptides (m/z 1291.664, 1458.701, 1588.781) normalized to total ion current. **(G-I)** Single ion images of tryptic peptides (m/z 958.566, 1550.809, and 1797) normalized to internal peptide standard. **(J-L)** Single ion images of elastase digested peptides (m/z 854.462, 906.472, and 1240.669) normalized to total ion count.

Supplemental Table S8. Elastase Proteomic Hits. Table summarizing elastase digested identified proteins with total spectrum counts and corresponding protein classifications derived from gene ontology terms.

Digest Identified Proteins (198)	Total Elastase Spectrum counts	GO TERM 1	GO TERM 2	GO TERM 3	Protein classification
Collagen alpha-1(I) chain	2481				Collagen
Collagen alpha-2(I) chain	1591				Collagen
Collagen alpha-1(III) chain	1219				Collagen
Collagen alpha-3(VI) chain	141				Collagen
Collagen alpha-1(II) chain	136				Collagen
Collagen alpha-1(VI) chain	68				Collagen
Collagen alpha-1(XIV) chain	58				Collagen
Collagen alpha-2(V) chain	58		Organized by protein group		
Collagen alpha-1(V) chain	30				Collagen
Collagen alpha-2(VI) chain	27				Collagen
Collagen alpha-1(X) chain	5				Collagen
Collagen alpha-1(VIII) chain	4				Collagen
Collagen alpha-6(VI) chain	10				Collagen
Collagen alpha-1(XII) chain	8				Collagen
					Collagen
Elastin	819		Organized by protein group		Elastin
Alpha-1-antitrypsin	15<br>11	GO:0062023 Collagen-containing extracellular matrix	GO:0030134 COPII-coated ER to Golgi transport vesicle	GO:0005783 Endoplasmic reticulum	Extracellular matrix
Galectin-1	2	GO:0062023 Collagen-containing extracellular matrix	GO:0005737 Cytoplasm	GO:0005788 Endoplasmic reticulum lumen	Extracellular matrix
Transforming growth factor-beta-ind	31	GO:0005604 Basement membrane	GO:0062023 Collagen-containing extracellular matrix	GO:0070062 Extracellular exosome	Extracellular matrix
Mimecan	37	GO:0062023 Collagen-containing extracellular matrix	GO:0070062 Extracellular exosome	GO:0031012 Extracellular matrix	Extracellular matrix
Periostin	24<br>15	GO:0062023 Collagen-containing extracellular matrix	GO:0031012 Extracellular matrix	GO:0005615 Extracellular space	Extracellular matrix
Decorin	11	GO:0062023 Collagen-containing extracellular matrix	GO:0005576 Extracellular region	GO:0005615 Extracellular space	Extracellular matrix
Fibronectin	18	GO:0016324 Apical plasma membrane	GO:0005604 Basement membrane (part of Collagen-containing extracellular matrix)	GO:0072562 Blood microparticle	Extracellular matrix
Lumican	10<br>6	GO:0062023 Collagen-containing extracellular matrix	GO:0070062 Extracellular exosome	GO:0031012 Extracellular matrix	Extracellular matrix
Tryptase beta-2	4	GO:0062023 Collagen-containing extracellular matrix	GO:0005615 Extracellular space	GO:1905368 peptidase complex	Extracellular matrix
Prolargin	3	GO:0062023 Collagen-containing extracellular matrix	GO:0005615 Extracellular space	GO:0031012 Extracellular matrix	Extracellular matrix

Biglycan	5	GO:0009986 Cell surface	GO:0062023 Collagen-containing extracellular matrix	GO:0070062 Extracellular exosome	Extracellular matrix
Microfibril-associated glycoprotein 4	3	GO:0062023 Collagen-containing extracellular matrix	GO:0071953 Elastic fiber	GO:0005576 Extracellular region	Extracellular matrix
Basement membrane-specific hepa	4	GO:0005604 Basement Collagenmembrane (part of Collagen-containing matrix extracellular matrix)	GO:0062023 containing extracellular	GO:0070062 Extracellular exosome<br>GO:0005788 endoplasmic reticulum lumen	Extracellular matrix
Olfactomedin-like protein 1		GO:0031012 Extracellular matrix	GO:0005615 Extracellular space	GO:0005615 Extracellular space	Extracellular matrix
Tenascin		GO:0005604 Basement Collagenmembrane (part of Collagen-containing matrix extracellular matrix)	GO:0062023 containing extracellular		Extracellular matrix
Kallistatin		GO:0070062 Extracellular exosome	GO:0005576 Extracellular region		Extracellular matrix

Pigment epithelium-derived factor	3	GO:0043203 axon hillock	GO:0005604 Basement containing extracellular Collagen-containing matrix extracellular matrix)	GO:0062023 Collagenmembrane (part of	Extracellular matrix
Cathepsin D	8	GO:0062023 Collagen-containing extracellular matrix	GO:0031904 endosome lumen	GO:0010008 endosome membrane	Extracellular matrix
Versican core protein	6	GO:0062023 Collagen-containing extracellular matrix	GO:0005788 endoplasmic reticulum lumen	GO:0031012 Extracellular matrix	Extracellular matrix
Fatty acid synthase	83	GO:0005737 Cytoplasm	GO:0005829 Cytosol	GO:0070062 Extracellular exosome	Cytosol
Peroxiredoxin-1	12	GO:0005737 Cytoplasm	GO:0005829 Cytosol	GO:0070062 Extracellular exosome	Cytosol
Triphosphate isomerase	9	GO:0005829 Cytosol	GO:0070062 Extracellular exosome	GO:0005615 Extracellular space	Cytosol
Annexin A6	6	GO:0062023 Collagen-containing extracellular matrix	GO:0005737 Cytoplasm	GO:0070062 Extracellular exosome<br>GO:0005615 Extracellular space	Cytosol
Phosphoglycerate kinase 1	7	GO:0005737 Cytoplasm	GO:0070062 Extracellular exosome	GO:0022626 Cytoplasmic ribosome	Cytosol
Large ribomal subunit protein uL4	4	GO:0005829 Cytosol	GO:0005737 Cytoplasm		Cytosol
Purine nucleide phosphorylase	5	GO:0005737 Cytoplasm	GO:0005829 Cytosol	GO:0070062 Extracellular exosome	Cytosol
4-trimethylaminobutyraldehyde deh	3	GO:0005737 Cytoplasm	GO:0005829 Cytosol	GO:0070062 Extracellular exosome	Cytosol
Asparagine--tRNA ligase, cytoplasm	5	GO:0005737 Cytoplasm	GO:0005829 Cytosol	GO:0070062 Extracellular exosome	Cytosol
Peroxiredoxin-6	3	GO:0035578 Azurophil granule lumen	GO:0005737 Cytoplasm	GO:0005829 Cytosol	Cytosol
Rho GDP-dissociation inhibitor 2	4	GO:0005737 Cytoplasm	GO:0031410 Cytoplasmic vesicle	GO:0005856 Cytoskeleton	Cytosol

Myosin regulatory light chain 12B	3 2	GO:0045177 Apical part of cell	GO:0005903 Brush border	GO:0005938 Cell cortex (part of cytoplasm)	Cytosol
Large ribomal subunit protein eL30		GO:0005829 Cytosol	GO:0005737 Cytoplasm	GO:0022626 Cytoplasmic ribosome	Cytosol
UTP--gluce-1-phphate uridylyltransf	2	GO:0005737 Cytoplasm	GO:0005829 Cytosol	GO:0070062 Extracellular exosome	Cytosol
Acylamino-acid-releasing enzyme	2	GO:0005829 Cytosol	GO:0070062 Extracellular exosome	GO:0005576 Extracellular region	Cytosol
L-lactate dehydrogenase A chain	2	GO:0005829 Cytosol	GO:0070062 Extracellular exosome	GO:0016020 Membrane	Cytosol
Ubiquitin thioesterase OTUB1	2	GO:0005829 Cytosol	GO:0070062 Extracellular exosome	GO:0005654 Nucleoplasm	Cytosol
Heat shock protein HSP 90-alpha	9	GO:0005829 Cytosol	GO:0070062 Extracellular exosome	GO:0048471 Perinuclear region of cytoplasm	Cytosol
Puromycin-sensitive aminopeptidas	4	GO:0005737 Cytoplasm	GO:0005829 Cytosol	GO:0070062 Extracellular exosome	Cytosol
DNA-dependent protein kinase cata	5	GO:0005737 Cytoplasm	GO:0005829 Cytosol	GO:0022626 Cytoplasmic ribosome	Cytosol
Proteasome subunit alpha type-5 (p	2	GO:0005737 Cytoplasm	GO:0005829 Cytosol	GO:0070062 Extracellular exosome	Cytosol
Glyceraldehyde-3-phosphate dehyd		GO:190411 axon cytoplasm	GO:0005829 Cytosol	GO:0070062 Extracellular exosome	Cytosol
Alpha-enolase	24 17	GO:0005938 Cell cortex (part of cytoplasm)	GO:0009986 Cell surface	GO:0005737 Cytoplasm	Cytosol
Annexin A5	14	GO:0062023 Collagen-containing extracellular matrix	GO:0005737 Cytoplasm	GO:0005829 Cytosol	Cytosol
Peptidyl-prolyl cis-trans isomerase A	14	GO:0005737 Cytoplasm	GO:0005829 Cytosol	GO:0070062 Extracellular exosome	Cytosol
Heat shock protein beta-1	9 8	GO:190411 axon cytoplasm	GO:0001533 Cornified envelope	GO:0005737 Cytoplasm	Cytosol
Ubiquitin-like modifier-activating enz		GO:0005829 Cytosol	GO:0005829 Cytosol	GO:0070062 Extracellular exosome	Cytosol
Peroxiredoxin-2	3	GO:0005737 Cytoplasm	GO:0005829 Cytosol		Cytosol
Myosin light polypeptide 6	7	GO:0005903 Brush border	GO:0005829 Cytosol	GO:0070062 Extracellular exosome	Cytosol
Glucose-6-phosphate isomerase	5	GO:0060170 Ciliary membrane	GO:0005829 Cytosol	GO:0070062 Extracellular exosome	Cytosol

Heterogeneous nuclear ribonucleop	9 2	GO:0071013 catalytic step spliceosome	GO:0005737 Cytoplasm	GO:0005829 Cytosol	Cytosol
Elongation factor 2	4	GO:0016235 aggresome	GO:0005737 Cytoplasm	GO:0005829 Cytosol	Cytosol
Rab GDP dissociation inhibitor beta	5	GO:0035578 Azurophil granule lumen	GO:0005737 Cytoplasm	GO:0005829 Cytosol	Cytosol
Nucleoside diphosphate kinase B	3	GO:0071944 Cell	GO:0005737 Cytoplasm	GO:0005829 Cytosol	Cytosol

	5	periphery			
Putative protein-lysine deacylase A	4	GO:0005737 Cytoplasm	GO:0005829 Cytosol	GO:0070062 Extracellular exosome	Cytosol
Cullin-associated NEDD8-dissociate	6	GO:0031461 cullin-RING ubiquitin ligase complex	GO:0005737 Cytoplasm	GO:0005829 Cytosol	Cytosol
UMP-CMP kinase	3	GO:0005737 Cytoplasm	GO:0005829 Cytosol	GO:0070062 Extracellular exosome	Cytosol
Prohibitin 1	3	GO:0009986 Cell surface	GO:0005737 Cytoplasm	GO:0005769 early endosome	Cytosol
Small ribomal subunit protein uS8	3	GO:0005737 Cytoplasm	GO:0005829 Cytosol	GO:0022626 Cytoplasmic ribosome	Cytosol
Calpain-1 catalytic subunit	3 3	GO:0110158 Calpain complex (a part of cytoplasm)	GO:0001533 Cornified envelope	GO:0005737 Cytoplasm	Cytosol
Small ribosomal subunit protein uS3		GO:0005737 Cytoplasm	GO:0005829 Cytosol	GO:0022626 Cytoplasmic ribosome	Cytosol
Small ribomal subunit protein uS4	2	GO:0005737 Cytoplasm	GO:0005829 Cytosol	GO:0022626 Cytoplasmic ribosome	Cytosol
Small ribosomal subunit protein eS8	3	GO:0005737 Cytoplasm	GO:0005829 Cytosol	GO:0022626 Cytoplasmic ribosome	Cytosol
Small ribosomal subunit protein eS2	3	GO:0005737 Cytoplasm	GO:0098556 cytoplasmic side of rough endoplasmic reticulum membrane	GO:0005829 Cytosol	Cytosol
RuvB-like 2	2	GO:0005829 Cytosol	GO:0120293 dynein axonemal particle (part of cytoplasm)	GO:0070062 Extracellular exosome	Cytosol
Small ribomal subunit protein uS5	2	GO:0005737 Cytoplasm	GO:0005829 Cytosol	GO:0022626 Cytoplasmic ribosome	Cytosol
Large ribomal subunit protein uL5	2	GO:0005737 Cytoplasm	GO:0005829 Cytosol	GO:0022626 Cytoplasmic ribosome	Cytosol
Cytoplasmic dynein 1 heavy chain 1	3	GO:190411 axon cytoplasm	GO:0035578 Azurophil granule lumen	GO:0005938 Cell cortex (part of cytoplasm)	Cytosol
Transgelin-2	17	GO:0015629 Actin cytoskeleton	GO:0005829 Cytosol	GO:0070062 Extracellular exosome	Cytoskeleton
Fructose-bisphosphate aldolase A		GO:0015629 Actin cytoskeleton	GO:0005829 Cytosol	GO:0070062 Extracellular exosome	Cytoskeleton
T-complex protein 1 subunit gamma	9 3	GO:0044297 Cell body	GO:0005832 Chaperone-containing T complex	GO:0005856 Cytoskeleton	Cytoskeleton
Adenylyl cyclase-associated protein	9	GO:0035578 Azurophil granule lumen	GO:0030864 Cortical actin cytoskeleton	GO:0005737 Cytoplasm	Cytoskeleton
Ras GTPase-activating-like protein	6 3	GO:0005884 Actin filament (in cytoskeletal GO term)	GO:0016324 apical plasma membrane	GO:0030424 axon	Cytoskeleton
Prelamin-A/C	3	GO:0005829 Cytosol	GO:0005882 Intermediate filament (in cytoskeletal GO term)	GO:0005638 Lamin filament	Cytoskeleton
Plastin-2	50	GO:0015629 Actin cytoskeleton	GO:0005829 Cytosol	GO:0005886 Plasma membrane	Cytoskeleton
Actin, cytoplasmic 1		GO:0015629 Actin cytoskeleton	GO:0005884 Actin filament (in cytoskeletal	GO:0005912 Adherens junction	Cytoskeleton

Filamin-A	36	GO:0015629 Actin cytoskeleton	GO term) GO:0005884 Actin filament (in cytoskeletal GO term)	GO:0032432 actin filament bundle	Cytoskeleton
Tubulin beta chain	41	GO:0005879 Axonemal microtubule (within cytoskeletal GO term)	GO:0035578 Azurophil granule lumen	GO:0005737 Cytoplasm	Cytoskeleton
Vimentin	35	GO:0030424 axon	GO:0031252 cell leading edge	GO:0005737 Cytoplasm	Cytoskeleton (next GO term)
Tubulin alpha-1A chain	24	GO:0005879 Axonemal microtubule (within cytoskeletal GO term)	GO:0000793 Condensed chromosome	GO:0005737 Cytoplasm	Cytoskeleton
Neuroblast differentiation-associated	22	GO:0015629 Actin cytoskeleton	GO:0044291 Cell to cell contact zone	GO:0043034 costamere	Cytoskeleton

Myosin-9		GO:0015629 Actin cytoskeleton	GO:0042641 Actomyosin	GO:0005826 actomyosin contractile ring	Cytoskeleton
Filamin-B	18	GO:0015629 Actin cytoskeleton	GO:0005903 Brush border	GO:0005938 Cell cortex (part of cytoplasm)	Cytoskeleton
Alpha-actinin-1	13	GO:0005903 Brush border	GO:0030054 Cell junction	GO:0042995 Cell projection	Cytoskeleton (next GO term)
Alpha-actinin-4	15	GO:0015629 Actin cytoskeleton	GO:0030054 Cell junction	GO:0042995 Cell projection	Cytoskeleton
Tropomyosin alpha-3 chain	14 14 6	GO:0015629 Actin Cytoskeleton	GO:0005884 Actin filament (in cytoskeletal GO term)	GO:0005856 Cytoskeleton	cytoskeleton
Gelsolin	11	GO:0030478 Actin cap (in cytoskeleton term)	GO:0015629 Actin actin cytoskeleton	GO:0072562 Blood microparticle	Cytoskeleton
Tropomyosin alpha-4 chain	8 6	GO:0005884 Actin GO:0030863 cortical GO term)	GO:0005856 filament (in cytoskeletal cytoskeleton	Cytoskeleton	Cytoskeleton
Profilin-1	5 7 2	GO:0072562 Blood microparticle	GO:0005938 Cell cortex (part of cytoplasm)	GO:0005737 Cytoplasm	Cytoskeleton (keyterm)
Elongation factor 1-alpha 1	6	GO:0030864 Cortical actin cytoskeleton		GO:0098574 cytoplasmic side of lysosomal membrane	Cytoskeleton
Dihydropyrimidinase-related protein	3	GO:0005856 Cytoskeleton	GO:0005829 Cytosol	GO:0070062 Extracellular exosome	Cytoskeleton
Actin-related protein 3	2	GO:0015629 Actin cytoskeleton	GO:0005885 Arp2/3 protein complex	GO:0005903 Brush border	Cytoskeleton
Cofilin-1		GO:0015629 Actin cytoskeleton	GO:0005737 Cytoplasm	GO:0005829 Cytosol	Cytoskeleton
Major vault protein		GO:0005737 Cytoplasm	GO:0005856 Cytoskeleton	GO:0005829 Cytosol	Cytoskeleton

Unconventional myosin-VI		GO:0015629 Actin filament cytoskeleton	GO:0005884 Actin (in cytoskeletal GO term)	GO:0045177 Apical part of cell	Cytoskeleton
Clusterin		GO:0005813 centrosome (part of cytoskeleton)	GO:0097546 ciliary base	GO:0097542 ciliary tip	Cytoskeleton
Coronin-1A		GO:0005884 Actin filament (in cytoskeletal GO term)	GO:0030424 axon	GO:0005911 cell-cell junction	Cytoskeleton
F-actin-capping protein subunit alph	2<br>2	GO:0015629 Actin cytoskeleton	GO:0005903 Brush border	GO:0030863 cortical cytoskeleton	Cytoskeleton
ATP synthase subunit alpha, mitoch	11	GO:0005743 Mitochondrial inner	GO:0005759 Mitochondrial matrix	GO:0005753 Mitochondrial proton-transporting ATP synthase membrane complex	Mitochondria
ATP synthase subunit beta, mitocho	10<br>4	GO:0005743 Mitochondrial inner	GO:0005759 Mitochondrial matrix	GO:0005753 Mitochondrial proton-transporting ATP synthase membrane complex	Mitochondria
Adenylate kinase 2, mitochondrial		GO:0005737 Cytoplasm	GO:0070062 Extracellular exosome	GO:0005758 Mitochondrial inner space	Mitochondria membrane
Aldehyde dehydrogenase, mitochon	6	GO:0005737 Cytoplasm	GO:0070062 Extracellular exosome	GO:0005758 Mitochondrial inner space	Mitochondria membrane
60 kDa heat shock protein, mitocho	4	GO:0009986 Cell surface	GO:0005905 Clathrincoated vesicle	GO:0030135 Coated vesicle	Mitochondria (annotated as mitochondrial)
Histone H2B type 1-K		GO:0005829 Cytosol	GO:0005615 Extracellular space	GO:0005654 Nucleoplasm	Nucleus
Histone H2A type 1	12<br>3	GO:0070062 Extracellular exosome	GO:0000786 Nucleosome	GO:0005634 Nucleus	Nucleus
Histone H4	6	GO:0043505 CENP-A containing nucleosome			Nucleus
X-ray repair crs-complementing prot	2	GO:0000781 chromosome, telomeric region	GO:0005829 Cytosol	GO:0070418 DNA-dependent protein kinase complex	Nucleus
Heterogeneous nuclear ribonucleop	11<br>6	GO:0005829 Cytosol	GO:0016020 membrane	GO:0005654 Nucleoplasm	Nucleus (keyword)
Heterogeneous nuclear ribonucleop	4	GO:0015030 Cajal body (part of nucleoplasm)	GO:0071013 catalytic step 2 spliceosome<br>GO:0016020 Membrane	GO:0000781 chromosome, telomeric	Nucleus region
Heterogeneous nuclear ribonucleop		GO:0005829 Cytosol		GO:0005654	

				Nucleoplasm	Nucleus
Histone H3.1	2	GO:0000786 Nucleosome	GO:0005634 Nucleus		Nucleus
Nucleolin	3	GO:0005938 Cell cortex (part of cytoplasm)	GO:0005694 Chromosome	GO:0001533 Cornified envelope	Nucleus
Immunoglobulin kappa constant	118	GO:0072562 Blood microparticle	GO:0070062 Extracellular exosome	GO:0005576 Extracellular region	Circulating
Albumin	84	GO:0072562 Blood microparticle	GO:0005737 Cytoplasm	GO:0005783 Endoplasmic reticulum	Circulating
Immunoglobulin heavy constant alp	8	GO:0072562 Blood microparticle	GO:0070062 Extracellular exosome	GO:0005576 Extracellular region	Circulating
Fibrinogen gamma chain	6	GO:0072562 Blood microparticle	GO:0009986 Cell surface	GO:0062023 Collagen-containing extracellular matrix	Circulating
Hemoglobin subunit alpha	5	GO:0072562 Blood microparticle	GO:0009986 Cell surface	GO:0062023 Collagen-containing extracellular matrix	Circulating
Apolipoprotein A-I	9	GO:0072562 Blood microparticle	GO:0005829 Cytosol	GO:007168 Endocytic vesicle lumen	Circulating
Alpha-2-macroglobulin	7	GO:0072562 Blood microparticle	GO:0062023 Collagen-containing extracellular matrix	GO:0031410 Cytoplasmic vesicle	Circulating
Putative stereocilin-like protein	8	GO:0072562 Blood microparticle	GO:0062023 Collagen-containing extracellular matrix	GO:0070062 Extracellular exosome	Circulating
Fibrinogen beta chain	5	GO:0072562 Blood microparticle	GO:0005938 Cell cortex (part of cytoplasm)	GO:0009986 Cell surface<br>GO:0005576 Extracellular region	Circulating
Immunoglobulin heavy constant ga	47	GO:0072562 Blood microparticle	GO:0070062 Extracellular exosome	GO:007168 Endocytic vesicle lumen	Circulating
Hemoglobin subunit beta	36	GO:0072562 Blood microparticle	GO:0005829 Cytosol	GO:0005601 Classicalcomplement-pathway C3/C5 convertase complex	Circulating
Complement C4-A	24	GO:0030424 Axon	GO:0072562 Blood microparticle	GO:0071682 Endocytic vesicle lumen	Circulating
Hemopexin	18<br>14	GO:0072562 Blood microparticle	GO:0062023 Collagen-containing extracellular matrix	GO:0009986 Cell surface<br>GO:0070062 Extracellular exosome	Circulating
Complement C3	16	GO:0035578 Azurophil granule lumen	GO:0072562 Blood microparticle		Circulating
Immunoglobulin heavy constant mu	6	GO:0072562 Blood microparticle	GO:0009986 Cell surface		Circulating
Alpha-1-antichymotrypsin	2<br>5	GO:0035578 Azurophil granule lumen	GO:0072562 Blood microparticle	GO:0062023 Collagen-containing extracellular matrix	Circulating
Ceruloplasmin	2	GO:0072562 Blood microparticle	GO:0005788 Endoplasmic reticulum lumen	GO:0070062 Extracellular exosome	Circulating
Alpha-1B-glycoprotein		GO:0072562 Blood microparticle	GO:0062023 Collagen-containing extracellular matrix	GO:0070062 Extracellular exosome	Circulating

Complement factor B		GO:0072562 Blood microparticle	GO:0005601 Classicalcomplement-pathway C3/C5 convertase complex	GO:0070062 Extracellular exosome	Circulating
Immunoglobulin kappa variable 3-2	3	GO:0072562 Blood microparticle	GO:0070062 Extracellular exosome	GO:0005576 Extracellular region	Circulating
Inter-alpha-trypsin inhibitor heavy ch	3	GO:0072562 Blood microparticle	GO:0070062 Extracellular exosome	GO:0005576 Extracellular region	Circulating
Heat shock 70 kDa protein 1A	16	GO:0016235 aggresome	GO:0072562 Blood microparticle	GO:0005814 Centriole	Multiple compartments
Heat shock protein HSP 90-beta	5	GO:0034751 aryl hydrocarbon receptor complex	GO:0044295 axonal growth cone	GO:0009986 Cell surface	Multiple compartments
Heat shock cognate 71 kDa protein	14	GO:0005776 autophagosome	GO:0072562 Blood microparticle	GO:0061202 clathrinsculpted gammaaminobutyric acid transport vesicle membrane	Multiple compartments
Pyruvate kinase PKM	8	GO:0005929 Cilium	GO:0062023 Collagen-containing extracellular matrix	GO:0005737 Cytoplasm	Multiple compartments
Transketolase	7	GO:0005829 Cytosol	GO:0005789 endoplasmic reticulum membrane	GO:0070062 Extracellular exosome	Multiple compartments
Protein disulfide-isomerase	5	GO:0005856 Cytoskeleton	GO:0005829 Cytosol	GO:0005783 endoplasmic reticulum	Multiple compartments
UDP-glucose 6-dehydrogenase	6<br>5	GO:0005829 Cytosol	GO:0070062 Extracellular exosome	GO:0005654 Nucleoplasm	Multiple compartments
Protein SET	5	GO:0000785 Chromatin	GO:0005737 Cytoplasm	GO:0005829 Cytosol	Multiple compartments
Transitional endoplasmic reticulum	5	GO:1904949 ATPase complex	GO:0035578 Azurophil granule lumen	GO:0005737 Cytoplasm	Multiple compartments
ATP-dependent RNA helicase A	4<br>5	GO:0015629 Actin cytoskeleton	GO:0005813 centrosome	GO:0070937 CRD-mediated mRNA stability complex	Multiple compartments
GTP-binding nuclear protein Ran	3	GO:0005814 Centriole	GO:0005737 Cytoplasm	GO:0005829 Cytosol	Multiple compartments
SERPINE1 mRNA-binding protein 1	2	GO:0005829 Cytosol	GO:0005634 Nucleus		Multiple compartments
Small ribomal subunit protein uS2B		GO:0022627 cytosolic small ribosomal subunit	GO:0005634 Nucleus	GO:0005886 Plasma membrane	Multiple compartments
Alpha-aminoadipic semialdehyde de		GO:0005829 Cytosol	GO:0070062 Extracellular exosome	GO:0005759 mitochondrial matrix	Multiple compartments
Proteasome activator complex subu	5	GO:0005829 Cytosol	GO:0016607 nuclear speck	GO:0005654 Nucleoplasm	Multiple compartments
Fumarate hydratase, mitochondrial	3	GO:0005694 Chromosome	GO:0005737 Cytoplasm	GO:0005829 Cytosol	Multiple compartments
26S proteasome non-ATPase regul	3	GO:0005829 Cytosol	GO:0070062 Extracellular exosome	GO:0005576 Extracellular region	Multiple compartments
Chymotrypsin-like elastase family m	4	GO:0005829 Cytosol	GO:0005576 Extracellular region	GO:0005615 Extracellular space	Multiple compartments
Endoplasmin (ER)	7	GO:0062023 Collagen-containing extracellular matrix	GO:0005829 Cytosol	GO:0071682 endocytic vesicle lumen	Keyword ER
Protein disulfide-isomerase A3	7	GO:0009986 Cell surface	GO:0005783 endoplasmic reticulum	GO:0005788 endoplasmic reticulum lumen	Keyword ER

Hypoxia up-regulated protein 1 (ER	2	GO:007168 Endocytic GO:0005783 endoplasmic<br>reticulum chaperone vesicle lumen reticulum		GO:0034663 endoplasmic<br>complex	Keyword ER
Calreticulin	16	GO:0005788 endoplasmic GO:0005789 endoplasmic GO:0005789 Nuclear<br>reticulum lumen reticulum membrane envelope			ER
Calnexin	3	GO:0005783 endoplasmic reticulum	GO:0005788 endoplasmic reticulum lumen	GO:0005789 endoplasmic reticulum membrane	ER
Acyl-CoA-binding protein	3	GO:0005783 endoplasmic reticulum	GO:0005788 endoplasmic reticulum lumen	GO:0070062 Extracellular exosome	ER
Desmoplakin	9	GO:0005912 Adherens junction	GO:0016323 Basolateral plasma membrane	GO:0001533 Cornified envelope	Membrane
Integrin alpha-V	2	GO:0009986 Cell surface	GO:0005829 Cytosol	GO:0009897 external side of plasma membrane	Membrane
Annexin A2	6	GO:0005912 Adherens junction	GO:0016324 Apical plasma membrane	GO:0016323 Basolateral plasma membrane	Membrane
Moesin	5	GO:0005912 Adherens junction	GO:0045177 Apical part of cell	GO:0044297 Cell body	Membrane
Vinculin	2	GO:0005912 Adherens junction	GO:0005903 Brush border	GO:0042995 Cell projection	Membrane
Chloride intracellular channel protei<br>n	3	GO:0034707 chloride channel complex (part of membrane term)	GO:0005737 Cytoplasm	GO:0070062 Extracellular exosome	Membrane
Chloride intracellular channel protei<br>n	2	GO:0072562 Blood microparticle	GO:0005903 Brush border	GO:0034707 chloride channel complex (part of membrane term)	Membrane
Tripeptidyl-peptidase 1 (lysosome)	3	GO:0005764 Lysosome			Other compartments
Alpha-galactosidase A (lysosome)	3	GO:0035578 Azurophil<br>granule lumenexosome	GO:0005737 Cytoplasm	GO:0070062 Extracellular	Other compartments
ADP-ribylation factor 3	5	GO:0070062 Extracellular exosome	GO:0000139 Golgi membrane	GO:0048471 Perinuclear region of cytoplasm	Other compartments
Clathrin heavy chain 1	4	GO:0030118 clathrin coat	GO:0030132 clathrin coat of coated pit vesicle	GO:0030130 clathrin coat of trans-Golgi network	Other compartments
Carboxypeptidase A1	12	GO:0005615 Extracellular space			Other compartments
Immunoglobulin lambda constant 7	15	GO:0005576 Extracellular GO:0005615 Extracellular GO:0071745 IgA			Other compartments

		region	space	immunoglobulin complex	
Immunoglobulin delta heavy chain	2	GO:0005615 Extracellular space	GO:0019814 Immunoglobulin complex	GO:0005886 Plasma membrane	Other compartments
Myoferlin	10	GO:0005901 Caveola	GO:0031410 Cytoplasmic vesicle	GO:0070062 Extracellular exosome	Other compartments
Plectin	5	GO:0030424 axon	GO:0005903 Brush border	GO:0043034 costamere	Other compartments
Synaptic vesicle membrane protein	3	GO:0035578 Azurophil granule lumen	GO:0005576 Extracellular region	GO:0070062 Extracellular exosome	Other compartments
Polymeric immunoglobulin receptor	2	GO:0035578 Azurophil granule lumen	GO:0070062 Extracellular exosome	GO:0005615 Extracellular space	Other compartments

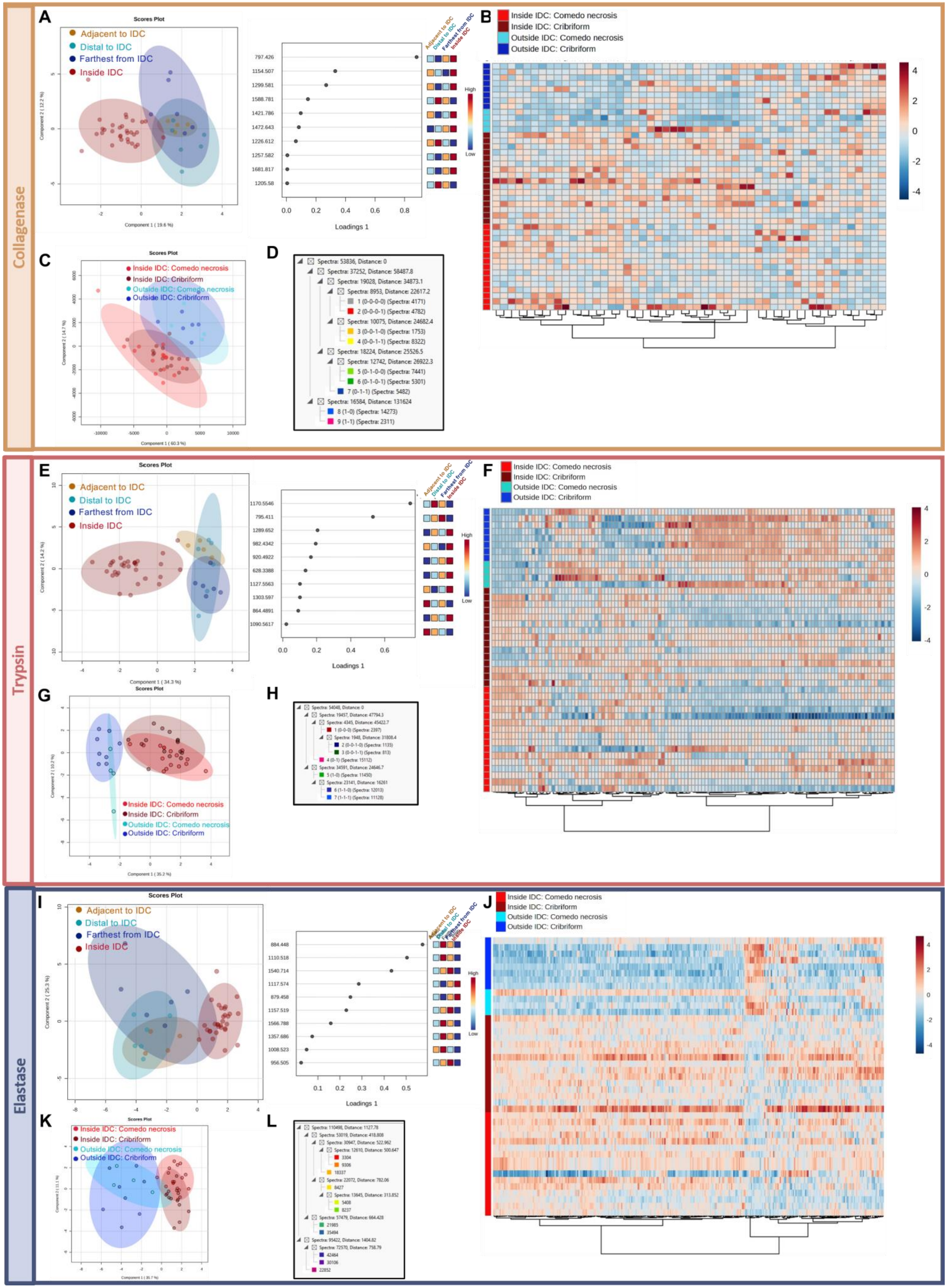
Collagenase
Trypsin
Elastase
A
Scores Plot
Adjacent to IDC
Distal to IDC
Farthest from IDC
Inside IDC
Component 1 (19.6 %)
Component 2 (12.2 %)
Loadings 1
High
Low
B
Inside IDC: Comedo necrosis
Inside IDC: Cribriform
Outside IDC: Comedo necrosis
Outside IDC: Cribriform
C
D
Spectra: 53836, Distance: 0
Spectra: 37252, Distance: 58487.8
Spectra: 19028, Distance: 34873.1
Spectra: 8953, Distance: 22617.2
1 (0-0-0-0) (Spectra: 4171)
2 (0-0-0-1) (Spectra: 4782)
Spectra: 10075, Distance: 24682.4
3 (0-0-1-0) (Spectra: 1753)
4 (0-0-1-1) (Spectra: 8322)
Spectra: 18224, Distance: 25526.5
Spectra: 12742, Distance: 26922.3
5 (0-1-0-0) (Spectra: 7441)
6 (0-1-0-1) (Spectra: 5301)
7 (0-1-1) (Spectra: 5482)
Spectra: 16584, Distance: 131624
8 (1-0) (Spectra: 14273)
9 (1-1) (Spectra: 2311)
E
F
G
H
I
J
K
L
Component 1 (34.3 %)
Component 1 (35.2 %)
Component 1 (60.3 %)

Supplemental Figure S7. Multiplexed Enzymatic Digest of DCIS076 Reports Distinct Proteomic Signature of IDC Lesions in Invasive Field. (A, E, I) Pathologist-defined lesions are represented in dark red for DCIS regions within the IDC region, in orange for DCIS lesions adjacent to the IDC region, light blue for lesions distal to IDC region within 1.0 mm of invasive border, and in dark blue for the farthest DCIS lesion from the invasive border (beyond 1.0 mm from the invasive region). Different architectural patterns denoted in different colors with cribriform represented in bright blue, comedo necrosis in cyan, solid in bright green, and IDC in bright red. **(A)** PLS-DA analysis of collagenase peptide peaks demonstrates minimal overlap between DCIS lesions inside the IDC regions and lesions of other distance classifications. **(B)** Heatmap of collagenase peptide peaks reports heterogeneity of ECM profile between comedo necrosis and cribriform architectural patterns. **(C)** PLS-DA analysis of collagenase peptide peaks shows overlap between DCIS lesions inside the IDC region regardless of architectural pattern. **(D)** Segmentation legend for 53 LC-MS/MS identified peptides from collagenase digest. Segmentation analysis was performed using bisecting K-means method with Manhattan metric. **(E)** PLS-DA analysis of tryptic peptide peaks demonstrates minimal overlap between DCIS lesions inside the IDC regions and lesions of other distance classifications. **(F)** Heatmap of tryptic peptide peaks reports heterogeneity of ECM profile between comedo necrosis and cribriform architectural patterns. **(G)** PLS-DA analysis of tryptic peptide peaks shows overlap between DCIS lesions inside the IDC region regardless of architectural pattern. **(H)** Segmentation legend for 128 putatively identified peptides from tryptic digest. Segmentation analysis was performed using bisecting K-means method with Manhattan metric. **(I)** PLS-DA analysis of elastase peptide peaks demonstrates minimal overlap between DCIS lesions inside the IDC regions and lesions of other distance classifications. **(J)** Heatmap of elastase peptide peaks reports heterogeneity of ECM profile between comedo necrosis and cribriform architectural patterns. **(K)** PLS-DA analysis of elastase peptide peaks shows overlap between DCIS lesions inside the IDC region regardless of architectural pattern. Metabolanalyst 5.0 was used to generate these PLS-DA analysis. **(L)** Segmentation legend for 112 LC-MS/MS identified peptides from elastase digest. Segmentation analysis was performed using bisecting K-means method with Manhattan metric.

A

1084.5018 m/z

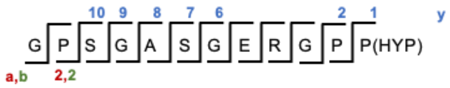

a^{+2}-ions	a-ions	b^{+2}-ions	b-ions				y-ions	y^{+2}-ions
-	-	-	-	1	G	12	-	-
-	127.0866	-	155.0815	2	P	11	1027.4844	514.2458
-	214.1186	-	242.1135	3	S	10	930.4316	465.7195
-	271.1401	-	299.1350	4	G	9	843.3996	422.2034
-	342.1772	-	370.1721	5	A	8	786.3781	393.6927
-	419.2092	-	457.2041	6	S	7	715.3410	358.1742
-	486.2307	-	514.2256	7	G	6	628.3090	314.6581
-	486.2092	-	643.2682	8	E	5	571.2875	286.1474
386.1908	615.2733	400.1883	799.3693	9	R	4	442.2449	221.6261
414.7016	771.3744	428.6990	856.3908	10	G	3	286.1438	-
463.2279	828.3959	477.2254	953.4435	11	P	2	229.1224	-
-	925.4486	-	-	12	P (HYP)	1	132.0696	-

08182022_Angel_Taylor_10F_273A6_DCIS # 3987 RT: 8.52 AV: 1 NL: 2.52E4
T: FTMS + c NSI d Full ms2 542.7592@hcd33.00 [112.9269-1129.2688]

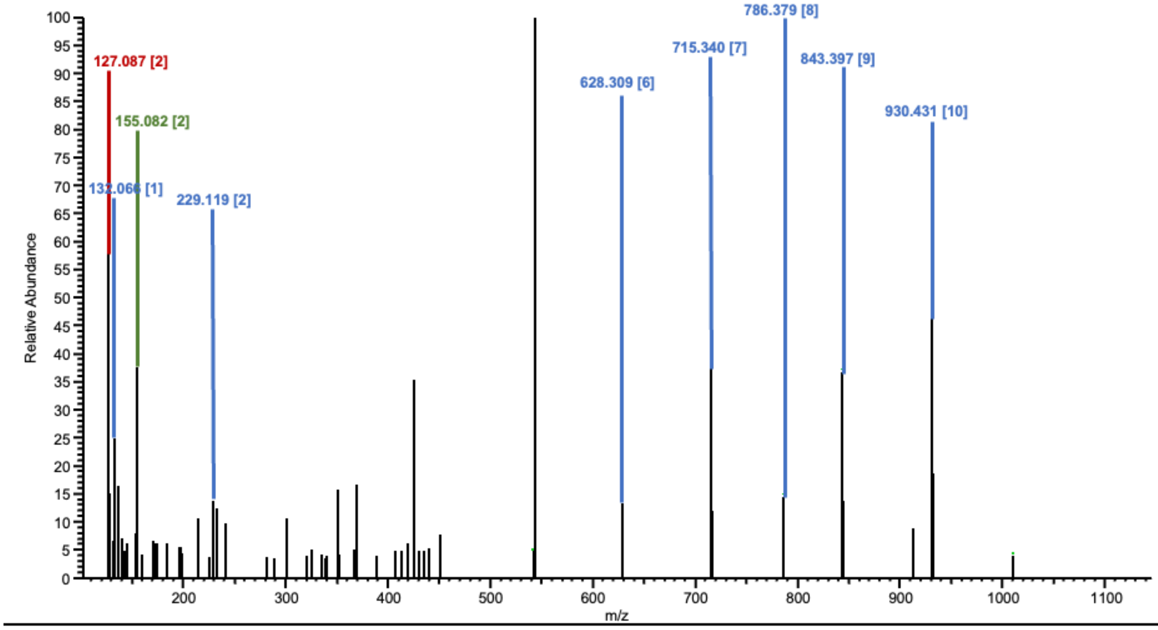

B

1089.4847 m/z

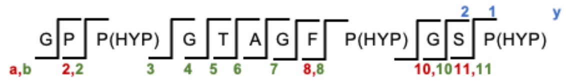

a-ions	b-ions				y-ions
-	-	1	G	12	-
127.0866	155.0815	2	P	11	1032.4755
240.1384	268.1333	3	P (HYP)	10	935.4228
297.1598	325.1547	4	G	9	822.3710
398.2075	426.2024	5	T	8	765.3495
469.2446	497.2395	6	A	7	664.3019
526.2661	554.2610	7	G	6	593.2647
673.3345	701.3294	8	F	5	536.2433
786.3863	814.3812	9	P (HYP)	4	389.1749
843.4077	871.4026	10	G	3	276.1231
930.4397	958.4347	11	S	2	219.1016
-	-	12	P (HYP)	1	132.0696

08182022_Angel_Taylor_4F_186C2_DCIS # 27066 RT: 56.77 AV: 1 NL: 4.23E5
T: FTMS + c NSI d Full ms2 545.2465@hcd33.00 [113.4343-1134.3428]

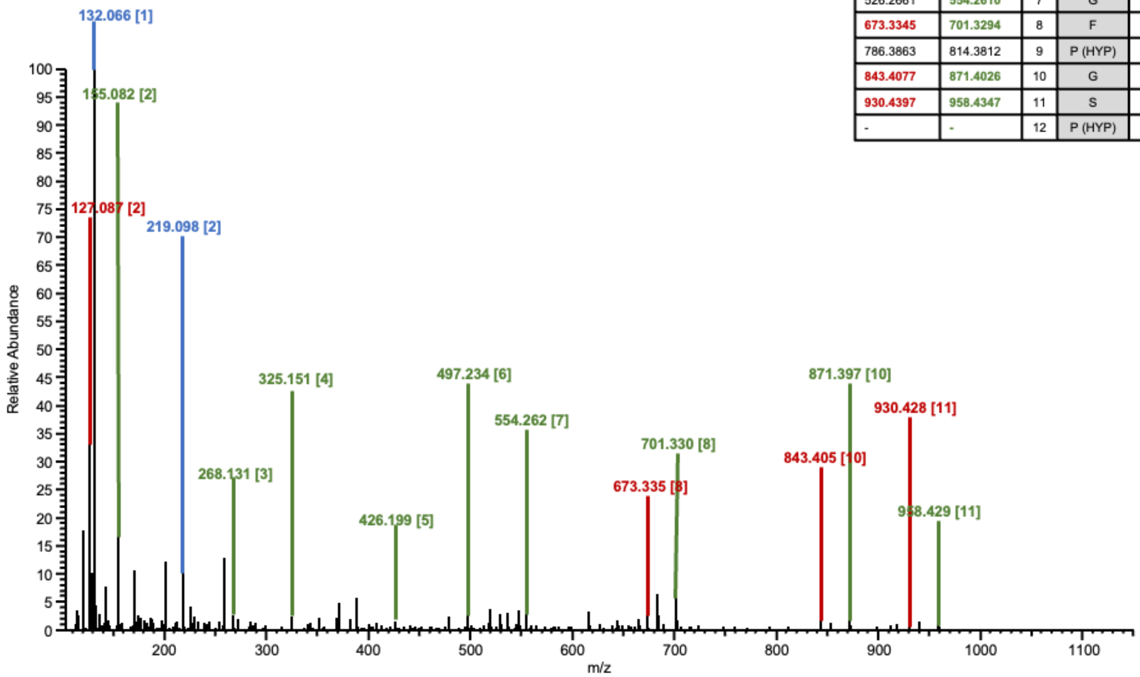

C

1283.5862 m/z

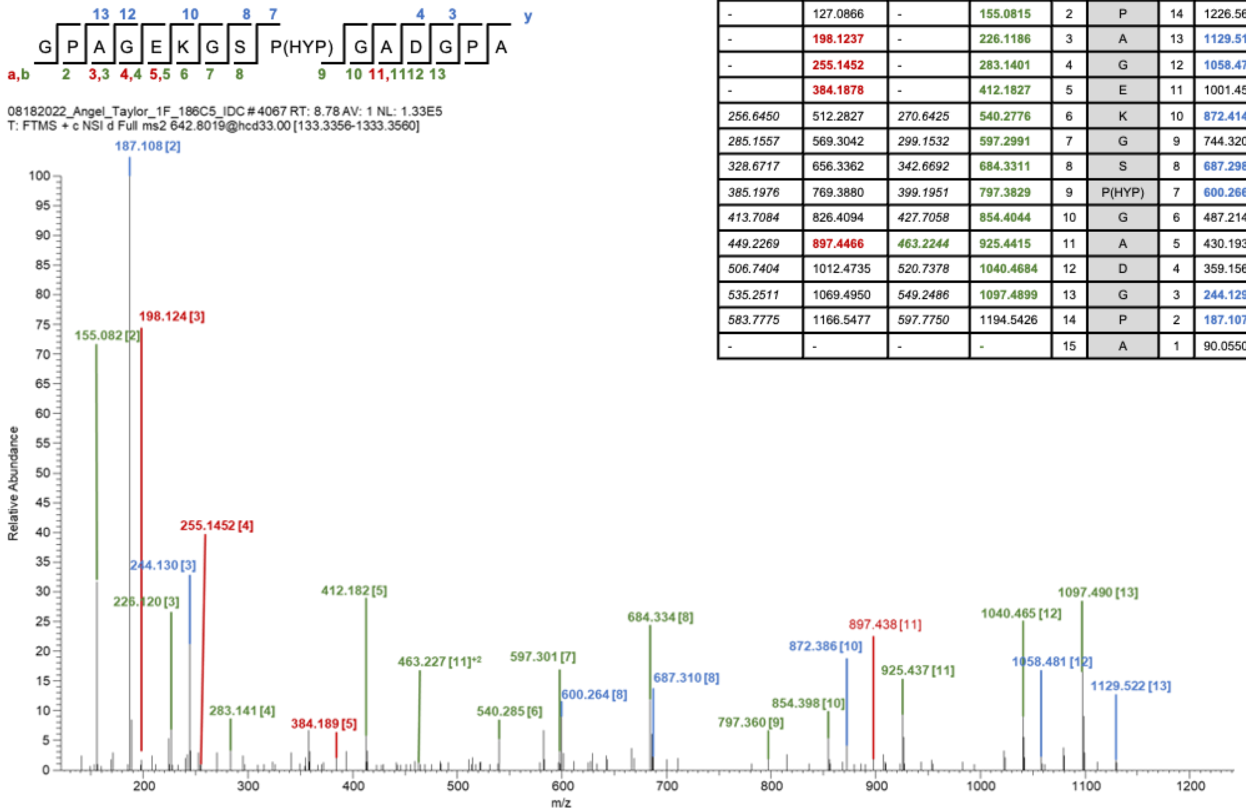

a⁺²-ions	a-ions	b⁺²-ions	b-ions				y-ions	y⁺²-ions
-	-	-	-	1	G	15	-	-
-	127.0866	-	155.0815	2	P	14	1226.5689	613.7881
-	198.1237	-	226.1186	3	A	13	1129.5161	565.2617
-	255.1452	-	283.1401	4	G	12	1058.4790	529.7431
-	384.1878	-	412.1827	5	E	11	1001.4575	501.2324
256.6450	512.2827	270.6425	540.2776	6	K	10	872.4149	436.7111
285.1557	569.3042	299.1532	597.2991	7	G	9	744.3200	-
328.6717	656.3362	342.6692	684.3311	8	S	8	687.2985	-
385.1976	769.3880	399.1951	797.3829	9	P(HYP)	7	600.2665	-
413.7084	826.4094	427.7058	854.4044	10	G	6	487.2147	-
449.2269	897.4466	463.2244	925.4415	11	A	5	430.1932	-
506.7404	1012.4735	520.7378	1040.4684	12	D	4	359.1561	-
535.2511	1069.4950	549.2486	1097.4899	13	G	3	244.1292	-
583.7775	1166.5477	597.7750	1194.5426	14	P	2	187.1077	-
-	-	-	-	15	A	1	90.0550	-

D

1060.527 m/z

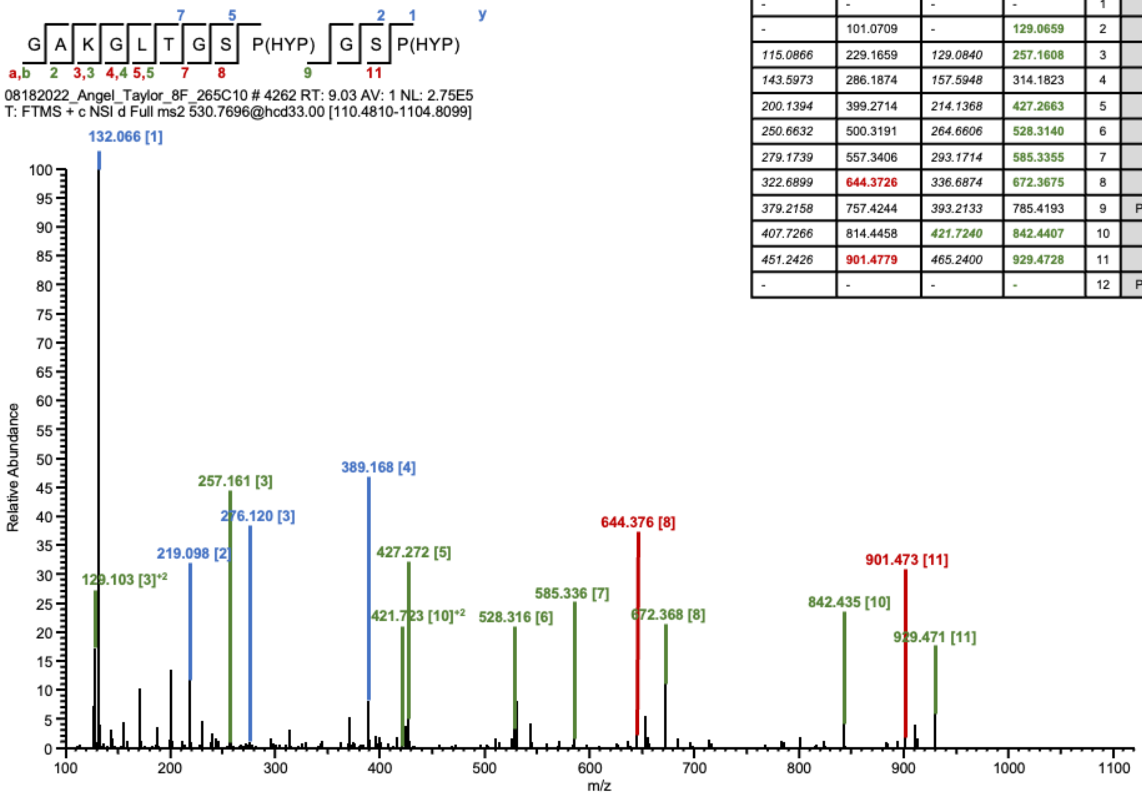

a⁺²-ions	a-ions	b⁺²-ions	b-ions				y-ions	y⁺²-ions
-	-	-	-	1	G	12	-	-
-	101.0709	-	129.0659	2	A	11	1003.5136	502.2605
115.0866	229.1659	129.0840	257.1608	3	K	10	932.4765	466.7419
143.5973	286.1874	157.5948	314.1823	4	G	9	804.3816	-
200.1394	399.2714	214.1368	427.2663	5	L	8	747.3601	-
250.6632	500.3191	264.6606	528.3140	6	T	7	634.2760	-
279.1739	557.3406	293.1714	585.3355	7	G	6	533.2284	-
322.6899	644.3726	336.6874	672.3675	8	S	5	476.2069	-
379.2158	757.4244	393.2133	785.4193	9	P(HYP)	4	389.1749	-
407.7266	814.4458	421.7240	842.4407	10	G	3	276.1231	-
451.2426	901.4779	465.2400	929.4728	11	S	2	219.1016	-
-	-	-	-	12	P(HYP)	1	132.0696	-

E

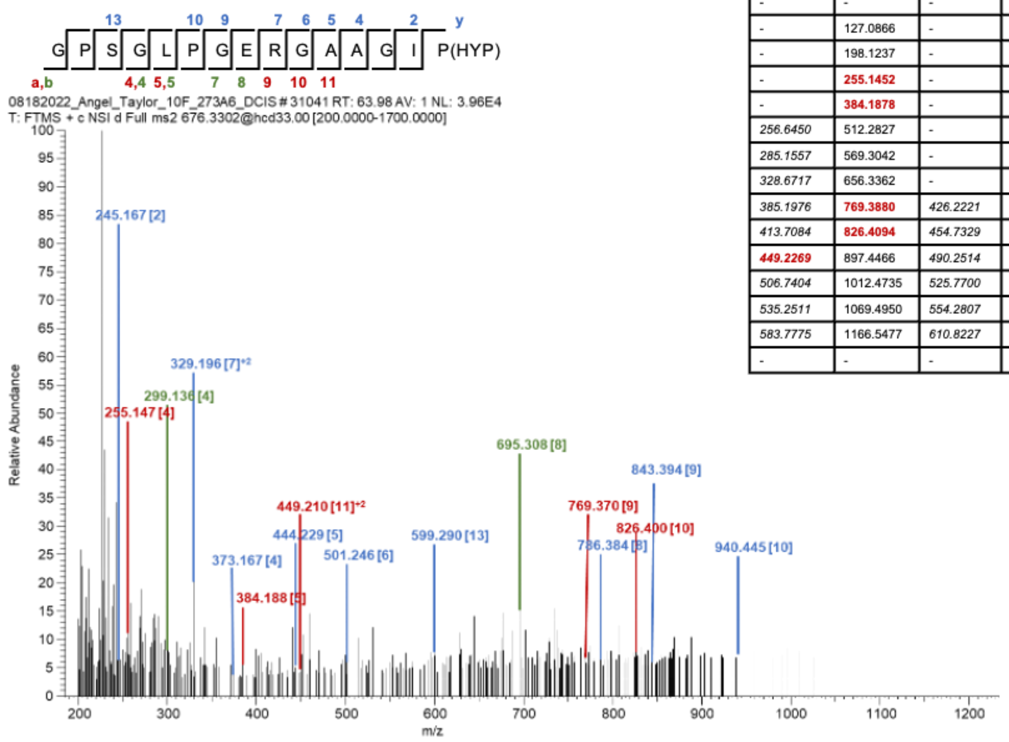

a^{+2}-ions	a-ions	b^{+2}-ions	b-ions				y-ions	y^{+2}-ions
-	-	-	-	1	G	15	-	-
-	127.0866	-	155.0815	2	P	14	1294.6791	647.8432
-	198.1237	-	242.1135	3	S	13	1197.6263	599.3168
-	255.1452	-	299.1350	4	G	12	1110.5943	555.8008
-	384.1878	-	412.2191	5	L	11	1053.5743	527.2900
256.6450	512.2827	-	509.2718	6	P	10	940.4888	470.7480
285.1557	569.3042	-	566.2933	7	G	9	843.4360	422.2216
328.6717	656.3362	-	695.3359	8	E	8	786.4145	393.7109
385.1976	769.3880	426.2221	851.4370	9	R	7	657.3719	329.1896
413.7084	826.4094	454.7329	908.4585	10	G	6	501.2708	-
449.2269	897.4466	490.2514	979.4956	11	A	5	444.2494	-
506.7404	1012.4735	525.7700	1050.5327	12	A	4	373.2122	-
535.2511	1069.4950	554.2807	1107.5541	13	G	3	302.1751	-
583.7775	1166.5477	610.8227	1220.6382	14	I	2	245.1537	-
-	-	-	-	15	P(HYP)	1	132.0696	-

F

1458.7006 m/z

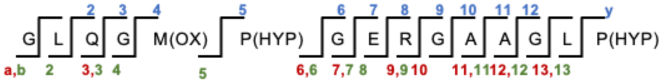

a^{+2}-ions	a-ions	b^{+2}-ions	b-ions				y-ions	y^{+2}-ions
-	-	-	-	1	G	15	-	-
-	143.1179	-	171.1128	2	L	14	1401.6873	701.3473
-	271.1765	-	299.1714	3	Q	13	1288.6032	644.8052
-	328.1979	-	356.1928	4	G	12	1160.5446	580.7759
-	475.2333	-	503.2282	5	M(OX)	11	1103.5232	552.2652
-	588.2851	-	616.2800	6	P(HYP)	10	956.4878	478.7475
-	645.3066	-	673.3015	7	G	9	843.4360	422.2216
-	774.3492	-	802.3441	8	E	8	786.4145	393.7109
465.7288	930.4503	479.7262	958.4452	9	R	7	657.3719	329.1896
494.2395	987.4717	508.2370	1015.4666	10	G	6	501.2708	-
529.7581	1058.5088	543.7555	1086.5038	11	A	5	444.2494	-
565.2766	1129.5460	579.2741	1157.5409	12	A	4	373.2122	-
593.7873	1186.5674	607.7848	1214.5623	13	G	3	302.1751	-
650.3294	1299.6515	664.3268	1327.6464	14	L	2	245.1537	-
-	-	-	-	15	P(HYP)	1	132.0696	-

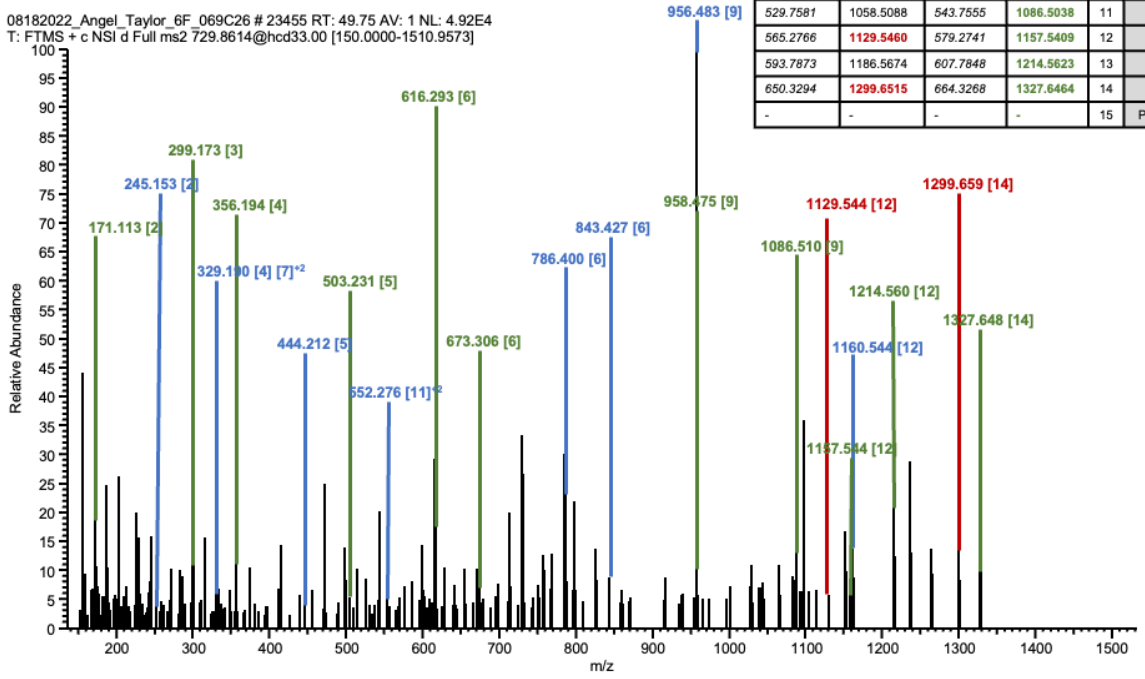

G 976.452 m/z

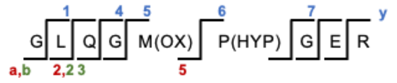

a-ions	b-ions				y-ions	y^{+2}-ions
-	-	1	G	9	-	-
143.1179	171.1128	2	L	8	919.4343	460.2208
271.1765	299.1714	3	Q	7	806.3502	403.6787
328.1979	356.1928	4	G	6	678.2916	339.6495
475.2333	503.2282	5	M(OX)	5	621.2702	311.1387
588.2851	616.2800	6	P(HYP)	4	474.2348	237.6210
645.3066	673.3015	7	G	3	361.1830	181.0951
774.3492	802.3441	8	E	2	304.1615	152.5844
-	-	9	R	1	175.1190	88.0631

08182022_Angel_Taylor_8F_265C10 # 4054 RT: 8.58 AV: 1 NL: 6.11E4
T: FTMS + c NSI d Full ms2 488.7317@hcd33.00 [101.9053-1019.0527]

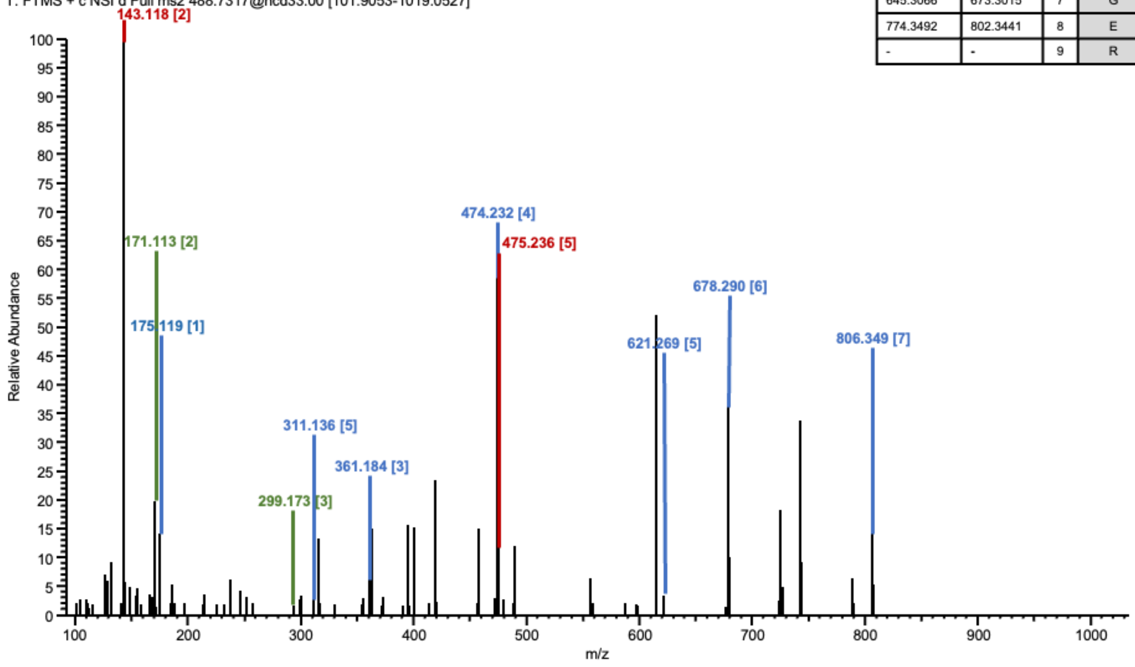

H 1125.5283 m/z

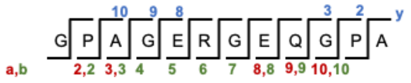

a-ions	b-ions				y-ions
-	-	1	G	12	-
127.0866	155.0815	2	P	11	1068.5069
198.1237	226.1186	3	A	10	971.4541
255.1452	283.1401	4	G	9	900.4170
384.1878	412.1827	5	E	8	843.3955
540.2889	568.2838	6	R	7	714.3529
597.3103	625.3052	7	G	6	558.2518
726.3529	754.3478	8	E	5	501.2304
854.4115	882.4064	9	Q	4	372.1878
911.4330	939.4279	10	G	3	244.1292
1008.4857	1036.4806	11	P	2	187.1077
-	-	12	A	1	90.0550

08182022_Angel_Taylor_1F_186C5_IDC # 4086 RT: 8.82 AV: 1 NL: 1.02E5
T: FTMS + c NSI d Full ms2 563.2692@hcd33.00 [117.1109-1171.1093]

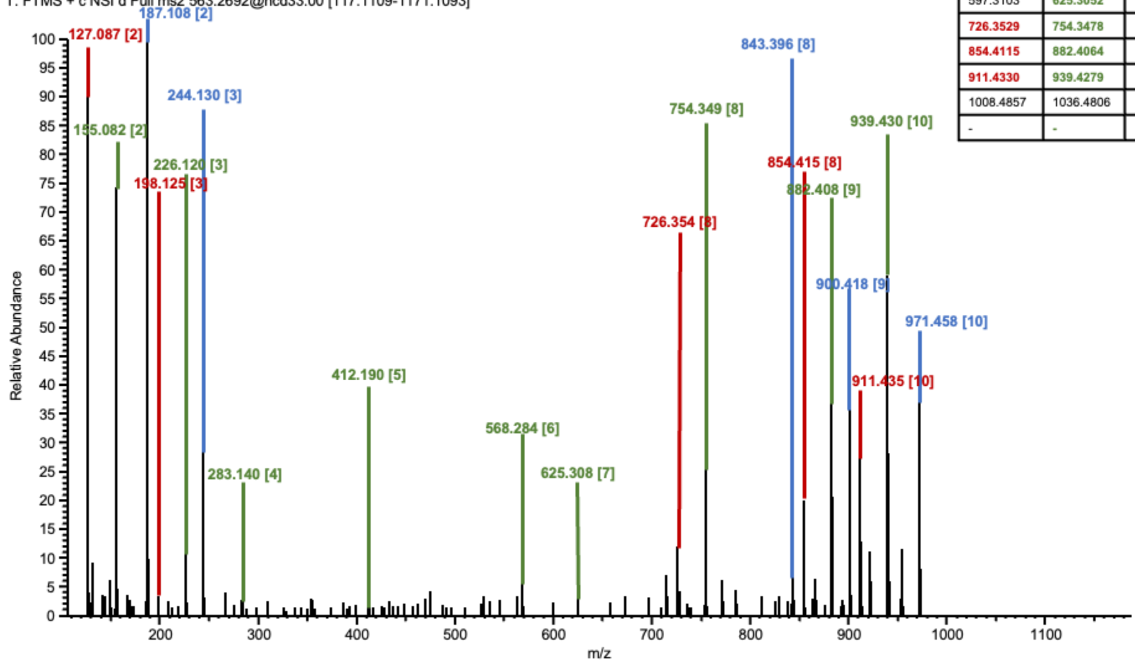

H

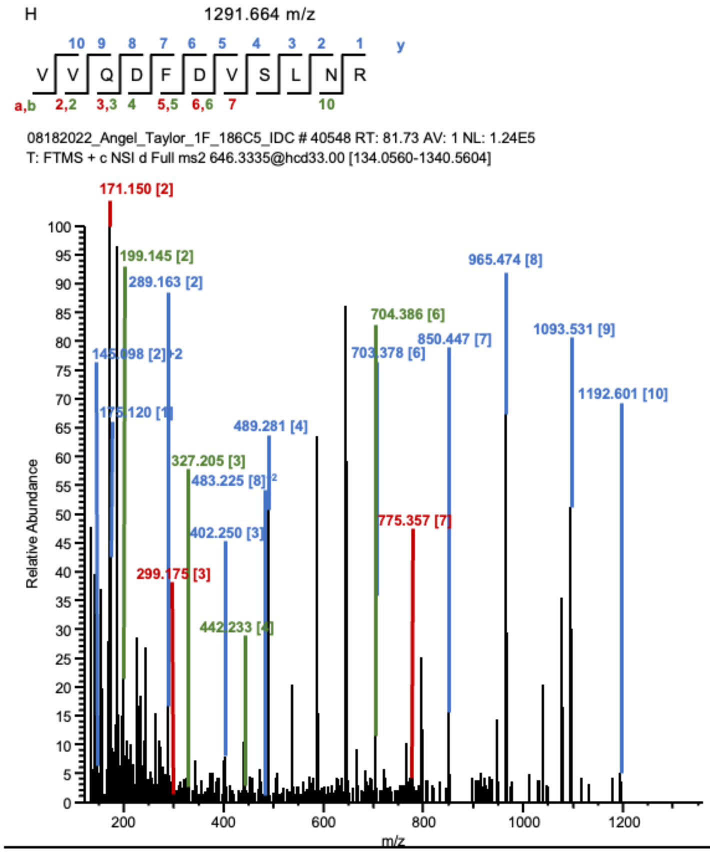

a-ions	b-ions				y-ions	y^{+2}-ions
-	-	1	V	11	-	-
171.1492	199.1441	2	V	10	1192.5957	596.8015
299.2078	327.2027	3	Q	9	1093.5273	547.2673
414.2347	442.2296	4	D	8	965.4687	483.2380
561.3031	589.2980	5	F	7	850.4417	425.7245
676.3301	704.3250	6	D	6	703.3733	352.1903
775.3985	803.3934	7	V	5	588.3464	294.6768
862.4305	890.4254	8	S	4	489.2780	245.1426
975.5146	1003.5095	9	L	3	402.2459	201.6266
1089.5575	1117.5524	10	N	2	289.1619	145.0846
-	-	11	R	1	175.1190	88.0631

I

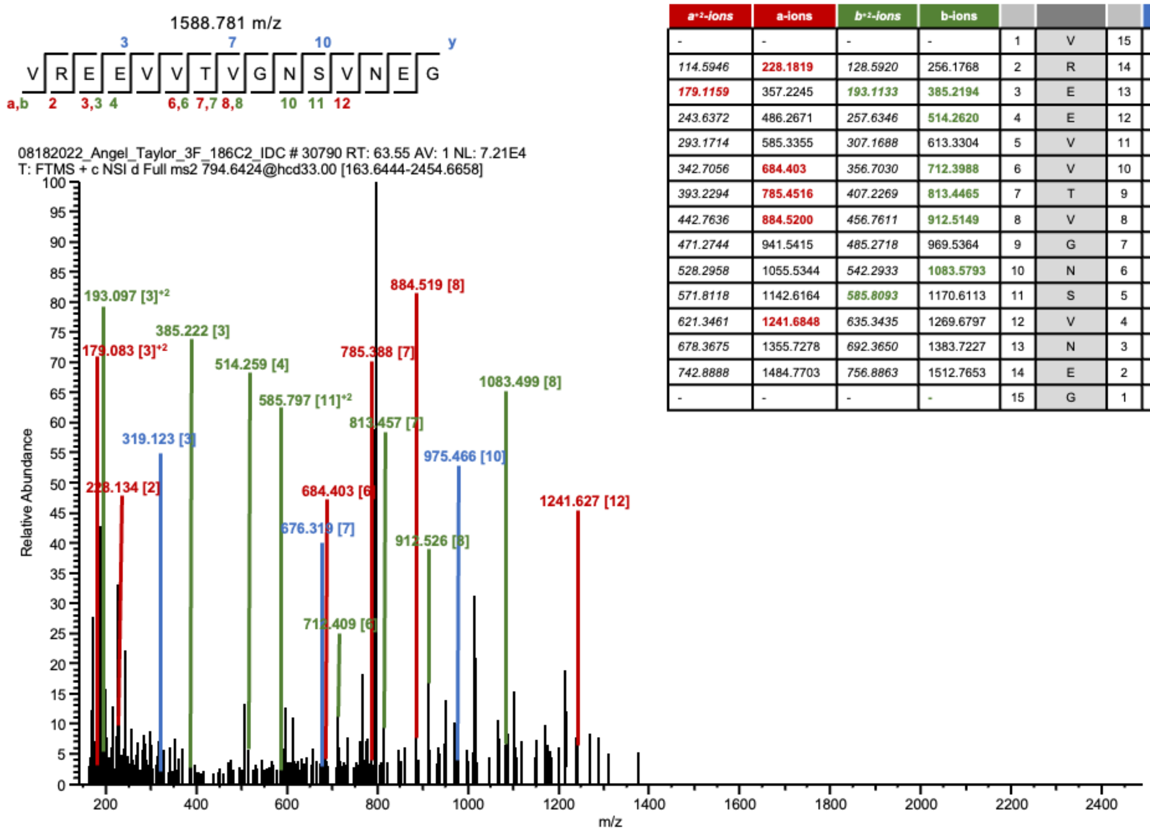

a^{+2}-ions	a-ions	b^{+2}-ions	b-ions				y-ions	y^{+2}-ions
-	-	-	-	1	V	15	-	-
114.5946	228.1819	128.5920	256.1768	2	R	14	1488.7289	744.8681
179.1159	357.2245	193.1133	385.2194	3	E	13	1332.6278	-
243.6372	486.2671	257.6346	514.2620	4	E	12	1203.5852	-
293.1714	585.3355	307.1688	613.3304	5	V	11	1074.5426	-
342.7056	684.403	356.7030	712.3988	6	V	10	975.4742	-
393.2294	785.4516	407.2269	813.4465	7	T	9	876.4058	-
442.7636	884.5200	456.7611	912.5149	8	V	8	775.3581	-
471.2744	941.5415	485.2718	969.5364	9	G	7	676.2897	-
528.2958	1055.5344	542.2933	1083.5793	10	N	6	619.2682	-
571.8118	1142.6164	585.8093	1170.6113	11	S	5	505.2253	-
621.3461	1241.6848	635.3435	1269.6797	12	V	4	418.1932	-
678.3675	1355.7278	692.3650	1383.7227	13	N	3	319.1248	-
742.8888	1484.7703	756.8863	1512.7653	14	E	2	205.0819	-
-	-	-	-	15	G	1	76.0393	-

Supplementary Figure S8. Annotated MS2 Spectrum Confirms Peptide Sequences from Collagenase Digest. (A-I) Annotated MS2 spectra of identified peptides from collagenase digest with matching theoretical a- (red), b- (green), and y-ions (blue) confirm sequences of interest from MSI data. Annotations were applied using the FreeStyle application and theoretical fragmentation pattern was acquired from ProteinProspector. Red denotes a-ions, green indicates green ions, and blue delineates y-ions.

A

958.569 m/z

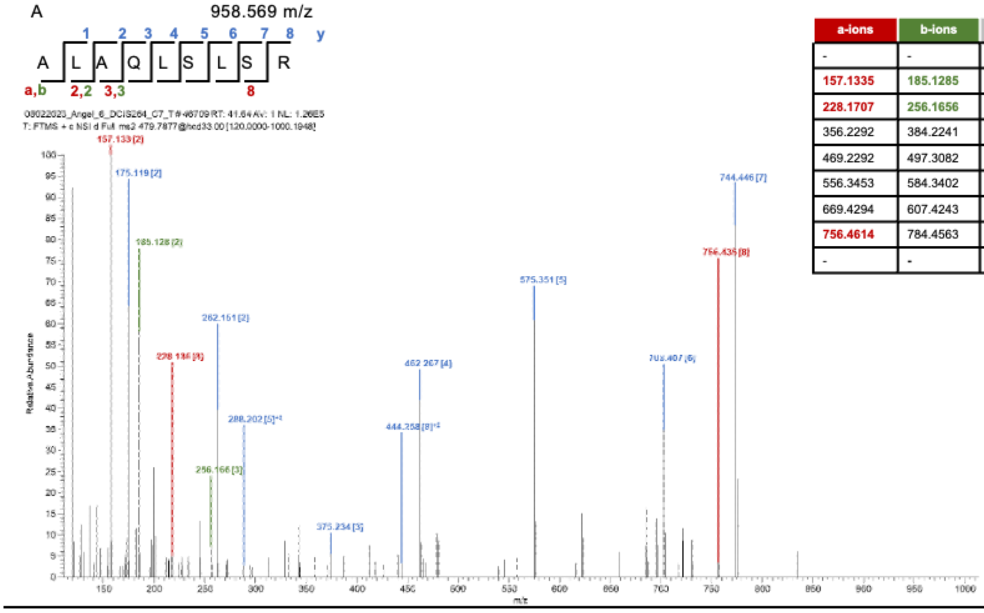

a-ions	b-ions				y-ions	y^{+2}-ions
-	-	1	A	9	-	-
157.1335	185.1285	2	L	8	887.5309	444.2692
228.1707	256.1656	3	A	7	774.4468	387.7271
356.2292	384.2241	4	Q	6	703.4097	352.2085
469.2292	497.3082	5	L	5	575.3511	288.1792
556.3453	584.3402	6	S	4	462.2671	231.6372
669.4294	607.4243	7	L	3	375.2350	188.1212
756.4614	784.4563	8	S	2	262.1510	131.5791
-	-	9	R	1	175.1190	88.0631

B

1045.564 m/z

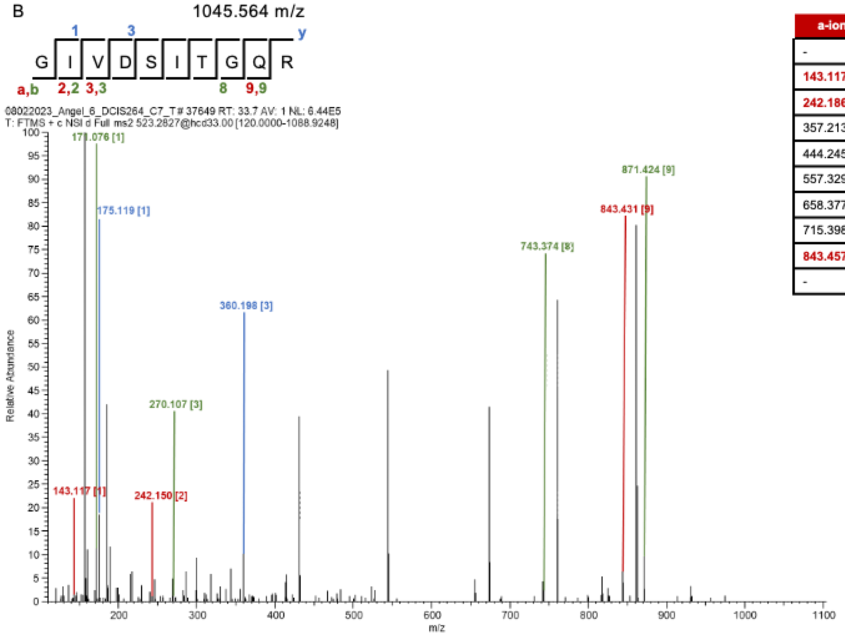

a-ions	b-ions				y-ions	y^{+2}-ions
-	-	1	G	10	-	-
143.1179	171.1128	2	I	9	988.5422	494.7747
242.1863	270.1812	3	V	8	875.4581	438.2327
357.2132	385.2082	4	D	7	776.3897	388.6985
444.2453	472.2402	5	S	6	661.3628	331.1850
557.3293	585.3243	6	I	5	574.3307	287.6690
658.3770	686.3719	7	T	4	461.2467	231.1270
715.3985	743.3934	8	G	3	360.1990	180.6031
843.4571	871.4520	9	Q	2	303.1775	152.0924
-	-	10	R	1	175.1190	88.0631

C

1240.671 m/z

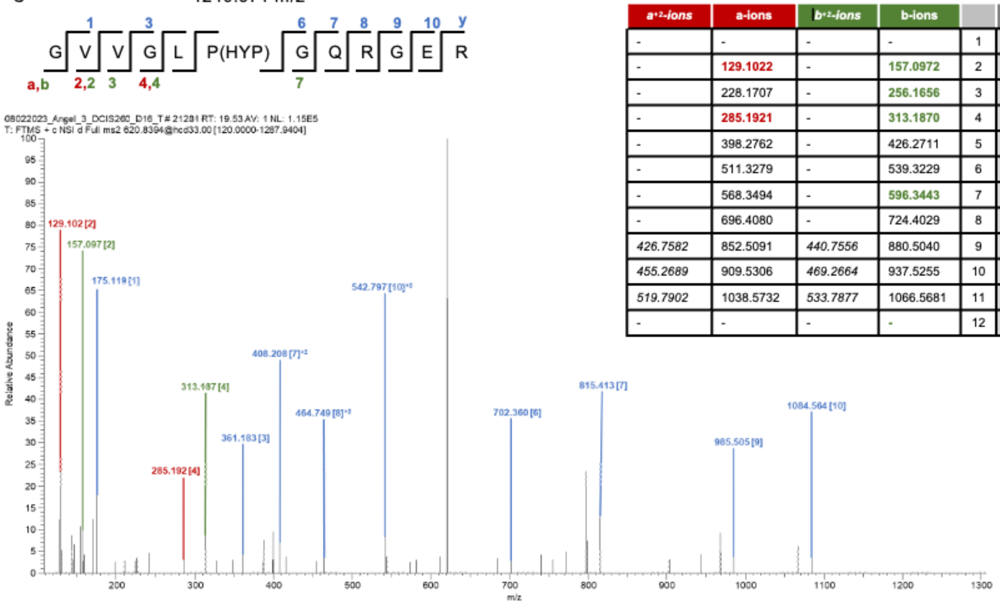

a^{+2}-ions	a-ions	b^{+2}-ions	b-ions				y-ions	y^{+2}-ions
-	-	-	-	1	G	12	-	-
-	129.1022	-	157.0972	2	V	11	1183.6583	592.3328
-	228.1707	-	256.1656	3	V	10	1084.5899	542.7986
-	285.1921	-	313.1870	4	G	9	985.5215	493.2644
-	398.2762	-	426.2711	5	L	8	928.5000	464.7536
-	511.3279	-	539.3229	6	P(HYP)	7	815.4159	408.2116
-	568.3494	-	596.3443	7	G	6	702.3642	351.6857
-	696.4080	-	724.4029	8	Q	5	645.3427	323.1750
426.7582	852.5091	440.7556	880.5040	9	R	4	517.2841	259.1457
455.2689	909.5306	469.2664	937.5255	10	G	3	361.1830	181.0951
519.7902	1038.5732	533.7877	1066.5681	11	E	2	304.1615	152.5844
-	-	-	-	12	R	1	175.1190	88.0631

D 1550.809 m/z

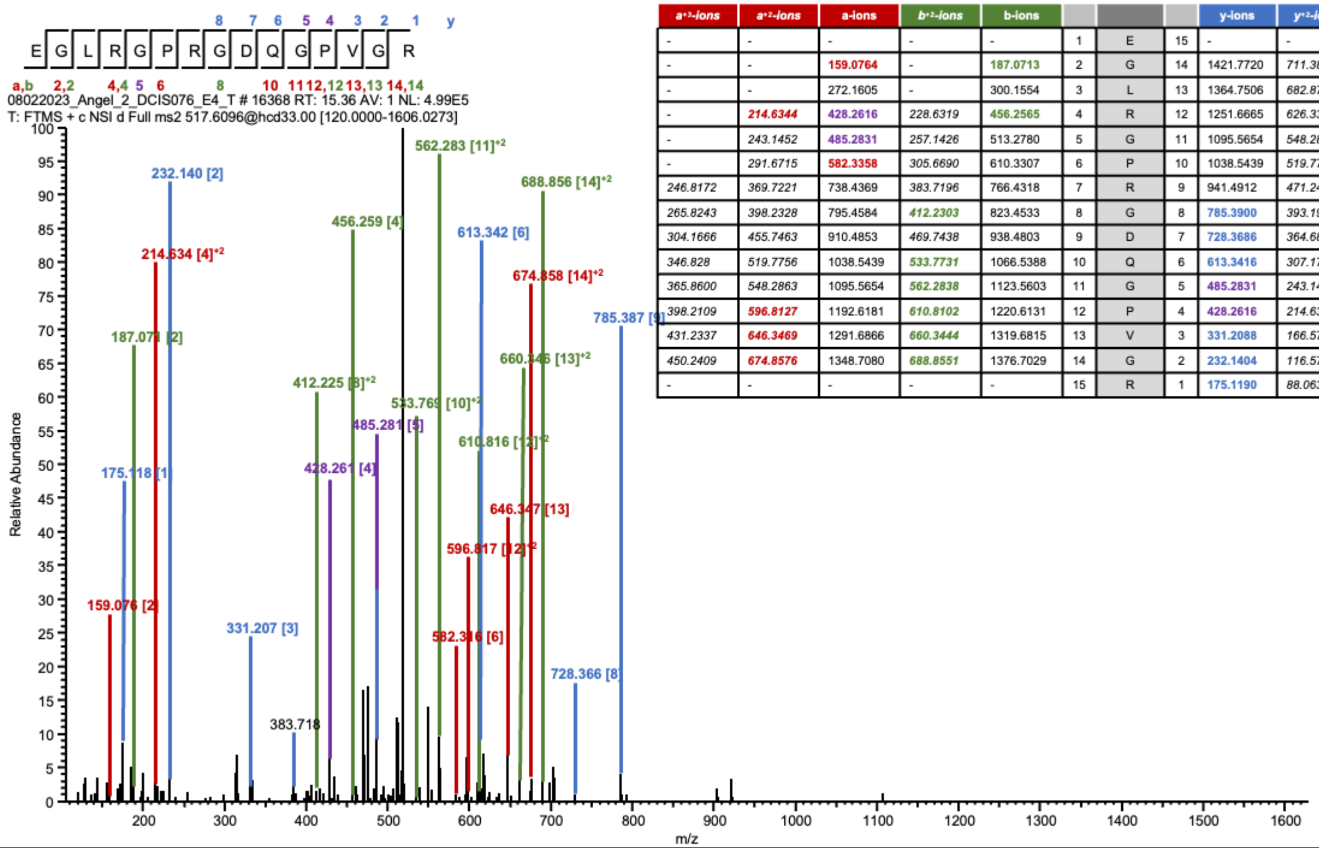

a+3-ions	a+2-ions	a-ions	b+2-ions	b-ions				y-ions	y+2-ions
-	-	-	-	-	1	E	15	-	-
-	-	159.0764	-	187.0713	2	G	14	1421.7720	711.3896
-	-	272.1605	-	300.1554	3	L	13	1364.7506	682.8782
-	214.6344	428.2616	228.6319	456.2565	4	R	12	1251.6665	626.3369
-	243.1452	485.2831	257.1426	513.2780	5	G	11	1095.5654	548.2863
-	291.6715	582.3358	305.6690	610.3307	6	P	10	1038.5439	519.7756
246.8172	369.7221	738.4369	383.7196	766.4318	7	R	9	941.4912	471.2492
265.8243	398.2328	795.4584	412.2303	823.4533	8	G	8	785.3900	393.1987
304.1666	455.7463	910.4853	469.7438	938.4803	9	D	7	728.3686	364.6879
346.828	519.7756	1038.5439	533.7731	1066.5388	10	Q	6	613.3416	307.1745
365.8600	548.2863	1095.5654	562.2838	1123.5603	11	G	5	485.2831	243.1452
398.2109	596.8127	1192.6181	610.8102	1220.6131	12	P	4	428.2616	214.6344
431.2337	646.3469	1291.6866	660.3444	1319.6815	13	V	3	331.2088	166.5738
450.2409	674.8576	1348.7080	688.8551	1376.7029	14	G	2	232.1404	116.5738
-	-	-	-	-	15	R	1	175.1190	88.0631

E 1797.841 m/z

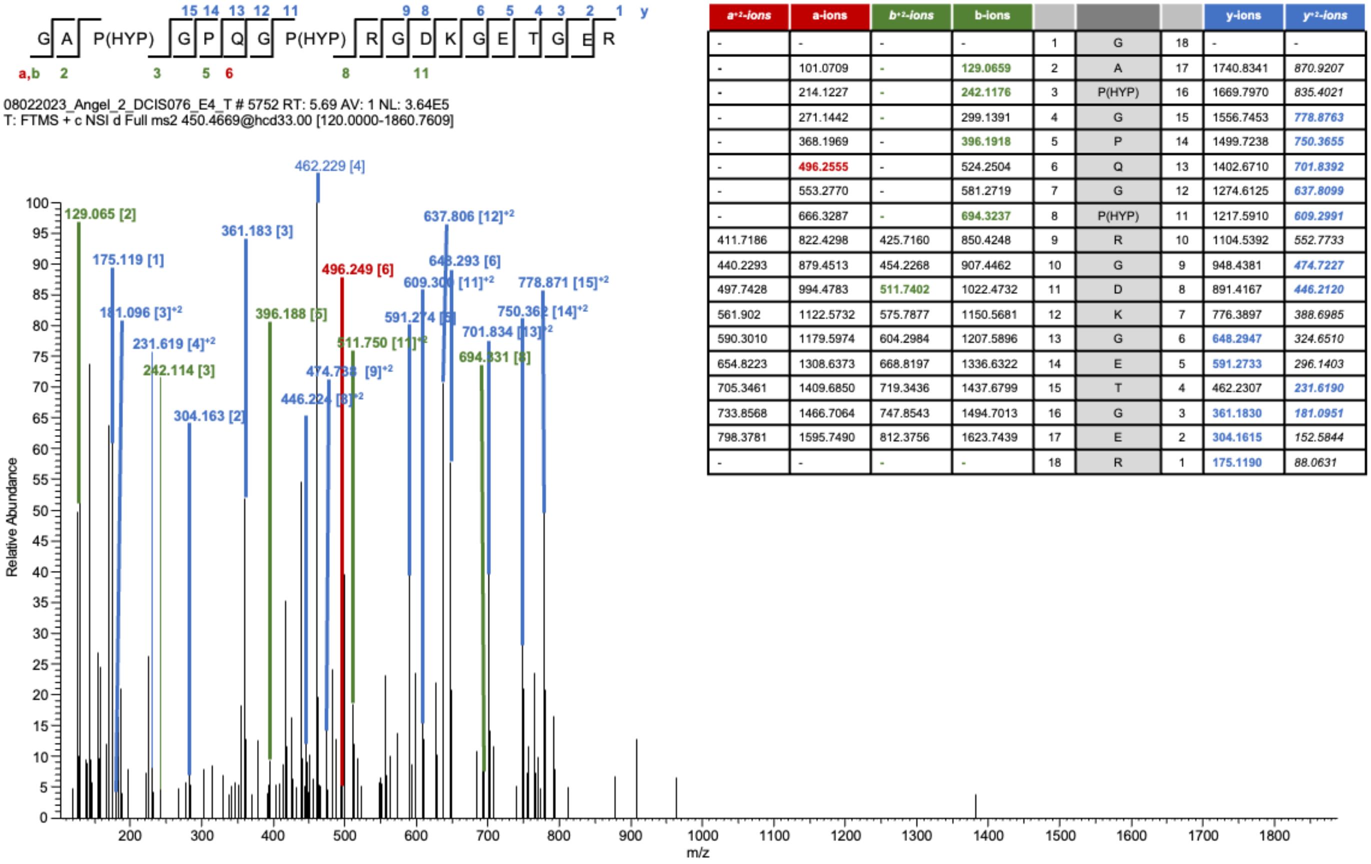

a+2-ions	a-ions	b+2-ions	b-ions				y-ions	y+2-ions
-	-	-	-	1	G	18	-	-
-	101.0709	-	129.0659	2	A	17	1740.8341	870.9207
-	214.1227	-	242.1176	3	P(HYP)	16	1669.7970	835.4021
-	271.1442	-	299.1391	4	G	15	1556.7453	778.8763
-	368.1969	-	396.1918	5	P	14	1499.7238	750.3655
-	496.2555	-	524.2504	6	Q	13	1402.6710	701.8392
-	553.2770	-	581.2719	7	G	12	1274.6125	637.8099
-	666.3287	-	694.3237	8	P(HYP)	11	1217.5910	609.2991
411.7186	822.4298	425.7160	850.4248	9	R	10	1104.5392	552.7733
440.2293	879.4513	454.2268	907.4462	10	G	9	948.4381	474.7227
497.7428	994.4783	511.7402	1022.4732	11	D	8	891.4167	446.2120
561.902	1122.5732	575.7877	1150.5681	12	K	7	776.3897	388.6985
590.3010	1179.5974	604.2984	1207.5896	13	G	6	648.2947	324.6510
654.8223	1308.6373	668.8197	1336.6322	14	E	5	591.2733	296.1403
705.3461	1409.6850	719.3436	1437.6799	15	T	4	462.2307	231.6190
733.8568	1466.7064	747.8543	1494.7013	16	G	3	361.1830	181.0951
798.3781	1595.7490	812.3756	1623.7439	17	E	2	304.1615	152.5844
-	-	-	-	18	R	1	175.1190	88.0631

Supplementary Figure S9. Annotated MS2 Spectrum Confirms Peptide Sequences from Tryptic Digest. (A-E) Annotated MS2 spectra of identified peptides from tryptic digest with matching theoretical a- (red), b- (green), and y-ions (blue) confirm sequences of interest from MSI data. Purple denotes ions that were defined as both a- and y-ions. Annotations were applied using the FreeStyle application and theoretical fragmentation patterns were acquired from ProteinProspector.
